# Structure and Dynamics in the Magnetotails of Unmagnetized and Weakly Magnetized Bodies

**DOI:** 10.1007/s11214-026-01307-5

**Published:** 2026-07-03

**Authors:** Katerina Stergiopoulou, David J. Andrews, Shannon M. Curry, Niklas J. T. Edberg, Mark Lester, Moa Persson, Norberto Romanelli, Shaosui Xu, Sae Aizawa, Christopher M. Fowler, Konstantin Kim, Yingjuan Ma, Robin Ramstad, Beatriz Sánchez-Cano

**Affiliations:** 1 Department of Physics, Umeå University, Umeå, Sweden; 2 School of Physics and Astronomy, University of Leicester, Leicester, UK; 3 Swedish Institute of Space Physics, Uppsala, Sweden; 4 Laboratory for Atmospheric and Space Physics, University of Colorado, Boulder, CO USA; 5 Department of Astronomy, University of Maryland, College Park, MD USA; 6 Planetary Magnetospheres Laboratory, NASA Goddard Space Flight Center, Greenbelt, MD USA; 7 Space Sciences Laboratory, University of California, Berkeley, CA USA; 8 Laboratoire de Physique des Plasmas (LPP), CNRS, Observatoire de Paris, Sorbonne Université, Université Paris Saclay, Ecole polytechnique, Institut Polytechnique de Paris, 91120 Palaiseau, France; 9 Department of Physics and Astronomy, West Virginia University, Morgantown, WV USA; 10 Department of Physics and Astronomy, Uppsala University, Uppsala, Sweden; 11 Department of Earth, Planetary and Space Sciences, University of California, Los Angeles, CA USA

**Keywords:** Mars, Venus, Induced magnetotails, Tail structure, Ion escape, Mars-Venus comparison

## Abstract

The plasma environment of our neighboring planets, Venus and Mars, differs significantly from Earth’s. Although neither of them possesses a dominant intrinsic dipolar magnetic field, there are still induced magnetospheres forming around the two planets, due to the interaction of the solar wind and the interplanetary magnetic field (IMF) with their conductive ionospheres, exospheres, and the localized crustal magnetic fields in the case of Mars. Induced magnetospheres, their associated plasma environments, and the physical processes within them are particularly susceptible to the changing upstream conditions. The increasing number of successful and long-lived missions during the last few decades has been key for describing the fundamental structures and processes comprising the induced magnetospheres of the two planets. Nevertheless, their induced magnetotails have been more challenging to probe, due to the restrictions of the orbital geometry of planetary missions. Here, we present the latest discoveries and a comprehensive comparison between the Venusian and Martian induced magnetotails, and we highlight the need for further exploration of these regions. Atmospheric escape and energy transfer processes and paths are inextricably linked with the climate history and the disappearance of water at Mars, though there are many unknowns still in the case of Venus. Past and current missions utilizing particle and fields instruments have explored a great part of the plasma environments of Venus and Mars. Several plasma boundaries, shaped by both internal and external factors, divide the planetary environments and magnetospheres into different plasma regimes and have been described by observations and models. Simulations and observations have also been utilized to investigate the magnetotail structure of the two planets, which appears to be governed mainly by the IMF, the solar wind dynamic pressure, and the crustal magnetic fields in the case of Mars. At Mars, the presence of the crustal magnetic fields, which are regions of crustal magnetization on the surface of the planet clustered mostly in the southern hemisphere, further complicates the interaction of the solar wind and the IMF with the planet’s plasma environment, thus justifying the term ‘hybrid’ – instead of induced – that is often used to describe the Martian magnetosphere. The existence of a magnetotail twist, as well as a first approach on mapping the structure of the current systems, has been reported at Mars, whereas different types of magnetotail current sheet flapping motion have been observed at both Mars and Venus. The magnetic topology, which describes the morphology of closed, open, and draped magnetic field lines over a planet, has also been inferred and explained for both planets. Escape processes, escape rates and their response to space weather have been reported, and we now have a better idea of the differences between the two planets. Escaping structures, contributing with a bulk removal of plasma, have also been observed in their magnetotails. Nevertheless, much still remains unknown, for example the specifics of how individual processes respond to solar drivers. Mars and Venus are not the only solar system bodies with no global intrinsic magnetic field. Induced magnetotails are formed around Saturn’s moon Titan and comets too. A comparison between those bodies and Venus and Mars will provide a broader and general picture of induced and hybrid magnetotails, which could help future investigations of the plasma environments and tails of exoplanets. Lastly, in this review paper, we also summarize the questions that remain unanswered, emphasizing the need for future missions.

## Introduction

Mars and Venus are the only planets in our Solar System without a dominant intrinsic dipolar magnetic field. Despite that, due to their conductive ionospheres that act as the effective obstacles to the incoming solar wind and the frozen-in interplanetary magnetic field (IMF), induced magnetospheres are formed around them (Schunk and Nagy 2009; Russell et al. 2016). Although the fundamental mechanism of formation of their induced magnetospheres is the same -which is true not only for Mars and Venus, but for other unmagnetized or weakly magnetized bodies with an atmosphere/ionosphere, such as comets and Saturn’s moon Titan- there are still features, namely their different sizes and distances from the Sun, as well as the presence of remnant patches of magnetic field on the Martian surface, influencing their morphology and creating a unique plasma environment in each case.

The magnetotail of an unmagnetized body is that part of the induced magnetosphere in the antisunward region of plasma and magnetic field, beyond the terminator, where the IMF drapes around the planet and forms a structured wake containing plasma originating from both the solar wind and the planetary environment. While the physical aspects of magnetotails are largely the same for intrinsic and induced magnetospheres (as discussed in Vasyliūnas (2015)), their detailed structures, dynamics, and roles in planetary plasma environments, such as their contribution to atmospheric loss, differ significantly. Unravelling the structure and dynamics of induced magnetotails is essential for the understanding of the physical pathways of atmospheric escape and energy transfer, both of which have had significant impacts on climate history. The first planetary flybys and missions to Mars and Venus, such as the Mariner, Mars, Viking, and Venera series paved the way for the successful and long-lived planetary missions that were launched during the last decades, such as the Mars Global Surveyor (MGS, Acuña et al. (1998)), Mars Express (MEX, Chicarro et al. (2004)), Mars Atmosphere and Volatile EvolutioN (MAVEN, Jakosky et al. (2015b)), Pioneer Venus Orbiter (PVO, Colin and Hunten (1977)), and Venus Express (VEX, Svedhem et al. (2007)) missions, as well as for the tail flybys from missions such as Solar Orbiter (SolO, Müller et al. (2020)), Parker Solar Probe (PSP, Raouafi et al. (2023)), BepiColombo (Benkhoff et al. 2021), and Rosetta (Taylor et al. 2017), which have contributed to a better understanding of induced magnetotails. Nonetheless, the dynamics and processes in the magnetotails of Mars and Venus are far from being thoroughly described and there are still aspects that need further investigation.

Geological evidence from Mars suggests a very different picture from the arid planet we see today, indicating that liquid water once flowed across the surface of the ancient planet (Jakosky 2021, 2022). Mars has almost certainly lost most of its atmosphere, as it is so thin today, yet we know that it must have been thicker in the past to support liquid water on the surface. (Jakosky 2019, 2022). Where did the thick atmosphere and the liquid water of ancient Mars go? At Venus, the current high Deuterium-to-Hydrogen (D/H) ratio is thought to point toward a historically wetter atmosphere, compared to the arid and hot one the planet has today (Donahue et al. 1982; Way et al. 2016), although this may have also been caused by a fractionated loss of hydrogen and an influx through volcanic sources and impact deliveries (Grinspoon 1993). It is unclear how much of Venus’s atmosphere has been lost since there still exists a thick atmosphere. Evidently, there are still questions remaining to be answered on how the evolution of the Venusian atmosphere has led to the extremely dense, arid, and hot place we see today (Gillmann et al. 2022).

In general, one may consider two pathways for the loss of atmospheric constituents from both Mars and Venus; one is through diffusion into the subsurface and the other is escaping to space (Jakosky 2019, 2022; Gronoff et al. 2020). Probing the nightsides and induced magnetotails of Mars and Venus, we can measure the loss rates of different atoms, molecules, and ions, extrapolate said rates into the past, and obtain the total loss of specific atmospheric constituents, as well as examine specific escape processes, their contribution to the total atmospheric escape, and their response to solar drivers (Jakosky 2021, 2022; Gillmann et al. 2022). Even though progress has been made on that topic, we are still a long way from a complete description of the history of water and atmospheric loss on both Mars and Venus. Venus has large gravity and a cold thermosphere, so its heavy atmospheric constituents, like oxygen, cannot escape Venus via thermal escape (e.g. Jeans escape) and non-thermal mechanisms are needed. This requires electromagnetic forces to act on charged particles to accelerate them, which in turn are governed by the magnetosphere and interaction with the solar wind. Additionally, to fully comprehend the structure and dynamics of the tail, besides capturing the escaping atmosphere with particle observations, mapping and understanding the magnetic topology and the tail response to solar (solar wind conditions, extreme ultraviolet -EUV- radiation, space weather) and planetary drivers (crustal magnetic fields) is necessary.

**Fig. 1:**
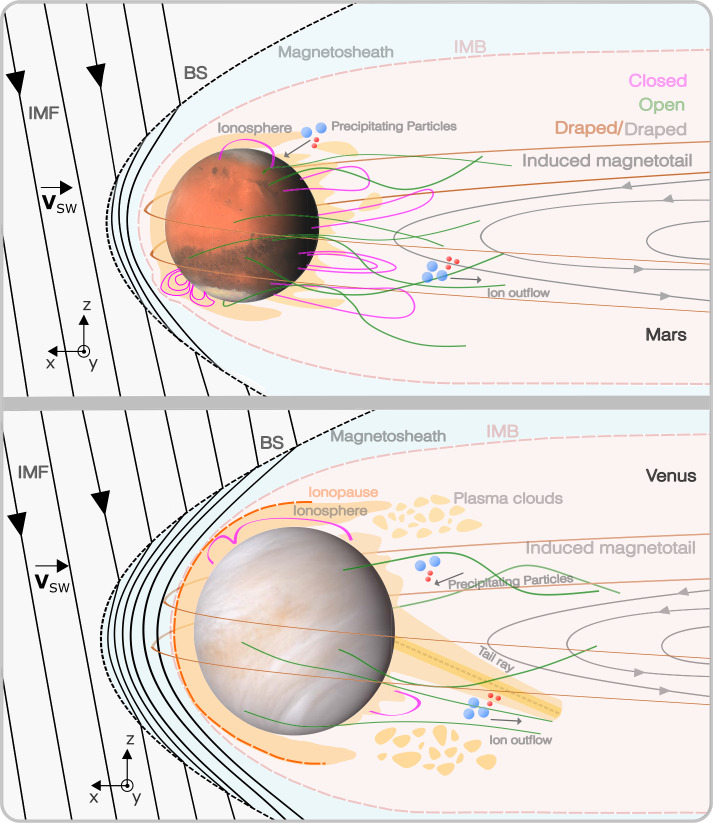
The induced magnetospheres and magnetotails of Mars (top) and Venus (bottom). Mars photo credit: ESA & MPS for OSIRIS Team MPS/UPD/LAM/IAA/RSSD/INTA/UPM/DASP/IDA. Content is used under the license: https://creativecommons.org/licenses/by-sa/3.0/igo/. Venus photo credit: Venus from Mariner 10, NASA/JPL-Caltech

The induced magnetospheres and magnetotails of Mars and Venus are illustrated in Fig. 1. As the incoming solar wind and the embedded IMF approach and interact with the conductive ionospheres of the planets, a bow shock (BS) is formed (BS in Fig. 1), at a greater distance in the case of Venus compared to the bow shock at Mars, and the frozen-in IMF drapes around the planets forming their magnetotails (Russell et al. 2016; Schunk and Nagy 2009). For additional descriptions and schematics of intrinsic and induced magnetotails see Vasyliūnas (2015) and Saunders and Russell (1986), respectively. The Martian bow shock is located at a distance of ${\sim} 1.58~\mathrm{R}_{M}$ at the subsolar point as both observations and models have shown (e.g. Vignes et al. 2000; Trotignon et al. 2006; Edberg et al. 2008), whereas the bow shock at Venus, at the subsolar point, appears to be fluctuating between $1.363~\mathrm{R}_{V}$ and $1.459~\mathrm{R}_{V}$ at solar minimum and maximum respectively (Shan et al. 2015; Signoles et al. 2023). The bow shock is then followed -as we move toward the planets- by the magnetosheath, a region characterized by slowed-down, heated, and turbulent solar wind (Fig. 1 - light blue) (Nagy et al. 2004). The induced magnetosphere boundary (IMB) also known as magnetic pile-up boundary (MPB), separates the magnetosheath from the induced magnetosphere or also known as the magnetic pile-up region (MPR), where planetary plasma dominates (Bertucci et al. 2011). As the IMF approaches the planets, it piles-up on the dayside and drapes around them forming their induced magnetotails on the nightside (Bertucci et al. 2011). In the case of Mars the term ‘hybrid’ is also used -instead of induced- to describe its magnetosphere and magnetotail, justified by the presence of the crustal magnetic fields, which complicate its magnetic topology and plasma environment. The term comes from the ‘combined’ magnetosphere first used by Dubinin et al. (1980). After that, authors gradually started using the term hybrid for the Martian magnetosphere (e.g Nagy et al. 2004; Dubinin et al. 2023). Besides the obvious differences in their radii and masses, the distinguishing feature between the two planets, in terms of their electromagnetic environments, is the presence of crustal magnetic fields on Mars, which influence and further complicate its escape processes and magnetic topology. The magnetic topology appears more complex at Mars (Xu et al. 2019) although unexpected closed magnetic field lines (Fig. 1 - magenta) have been reported to exist at Venus too (Xu et al. 2021, 2024). Bulk ion escape, observed as escaping structures on the nightsides of the two planets, also shows notable differences. The nightside ionospheres are highly dynamic at both planets and, as shown in Fig. 1, there is a main tail ray extending for thousands of kilometers at Venus with plasma clouds possibly escaping from the sides (Brecht and Ledvina 2021; Collinson et al. 2021; Brace et al. 1982, 1987). While there is no tail ray found at Mars there are still escaping plasma structures reported at high altitudes beyond the terminator, on the nightside part of the planet (Brecht and Ledvina 2021; Brain et al. 2010b; Halekas et al. 2016; Stergiopoulou et al. 2020).

The aim of this article is to review the broad aspects of the hybrid and induced magnetotails of Mars and Venus and summarize the results of the last few decades. This includes a wide range of physical processes, which often connect to the overall plasma environment (the dayside, terminator, and ionosphere region), making it necessary to also mention processes ongoing in those regions, while the main focus is still on the tail. A shorter comparison to other bodies is also included toward the end for broader context. In Sect. 2, previous Mars and Venus missions, their instrumentation, and their coverage over the planets are described to demonstrate which regions and processes have been investigated and what remains to be explored. In Sect. 3, the boundaries that are formed around non- or weakly magnetized planets and the drivers of their variability are explained. The induced magnetotails of Mars and Venus are compared in Sect. 4, which is a detailed summary of their tail structure from observations and simulations thus far. In Sect. 5, ion escape processes and escaping structures in the tails, as well as the factors influencing them are reported. In Sect. 6, we review what we know about the induced magnetotails of other bodies such as Saturn’s moon Titan, comets and exoplanets, and how discoveries on the Martian and Venusian tails could contribute to broaden our understanding of induced magnetotails. Lastly, in Sect. 7, open questions about induced magnetotails are discussed, and the future needs in the planetary exploration of induced magnetospheres and magnetotails are addressed.

## Previous Missions and Measurements in the Magnetotails of Mars and Venus

**Fig. 2:**
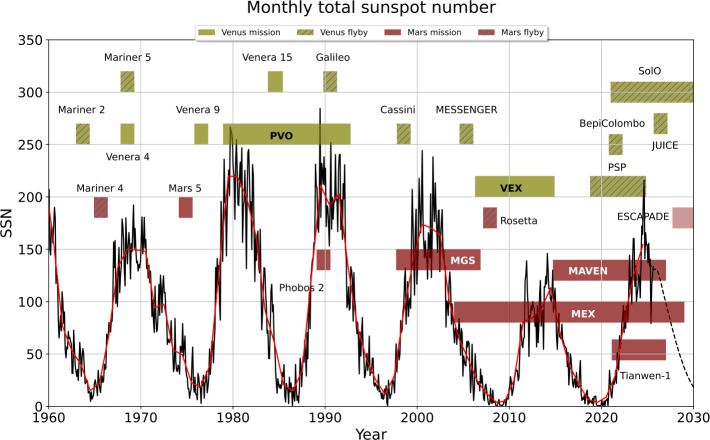
A timeline of plasma related missions at Mars and Venus over time with respect to the sunspot number (SSN). Solid colors represent designated missions at Mars (red) and Venus (green) while stripes denote a flyby (gravity assist). The data for the SSN over the past years were taken from WDC-SILSO, Royal Observatory of Belgium, Brussels (Clette and Lefèvre 2015) and they are used under the license: https://creativecommons.org/licenses/by-nc/4.0/. The predicted SSN until 2030 were taken from the Space Weather Prediction Center, National Oceanic and Atmospheric Administration (NOAA)

Previous missions with plasma instrumentation at Mars and Venus have covered more than 4 solar cycles, beginning with the Venera series in 1967 (Reese and Swan 1968) and the dedicated Venus orbiter PVO in 1978 at Venus, and the Mars series orbiters in 1971 (Dolginov et al. 1976; Ainbund et al. 1973; Gringauz et al. 1974), and Phobos 2 in 1989 (Sagdeev and Zakharov 1989) at Mars. Figure 2 illustrates the missions with plasma and/or fields instrumentation at Mars (red) and Venus (green) from 1960, roughly 4 years before the start of solar cycle 20, until 2030. We note that there are complementary measurements from other missions relevant to solar, atmospheric or ionospheric research such as the Radiation Assessment Detector (RAD, Hassler et al. (2012)) on Mars Science Laboratory (MSL, Grotzinger et al. (2012)) or the SHAllow RADar (SHARAD, Seu et al. (2007)) aboard Mars Reconnaissance Orbiter (MRO, Zurek and Smrekar (2007)) that are not shown in the Figure. Since the launch of MGS in 1997, there has been a continuous presence of orbiters around Mars. Furthermore, from 2015 onward there have been at least two spacecraft with plasma instrumentation simultaneously orbiting Mars, providing better coverage, multi-point measurements, and improving the reliability of measurements. In contrast, as shown in Fig. 2, there was a large gap between PVO and VEX, with no dedicated Venus missions, and it has been now more than 10 years since the end of the VEX mission.

**Fig. 3:**
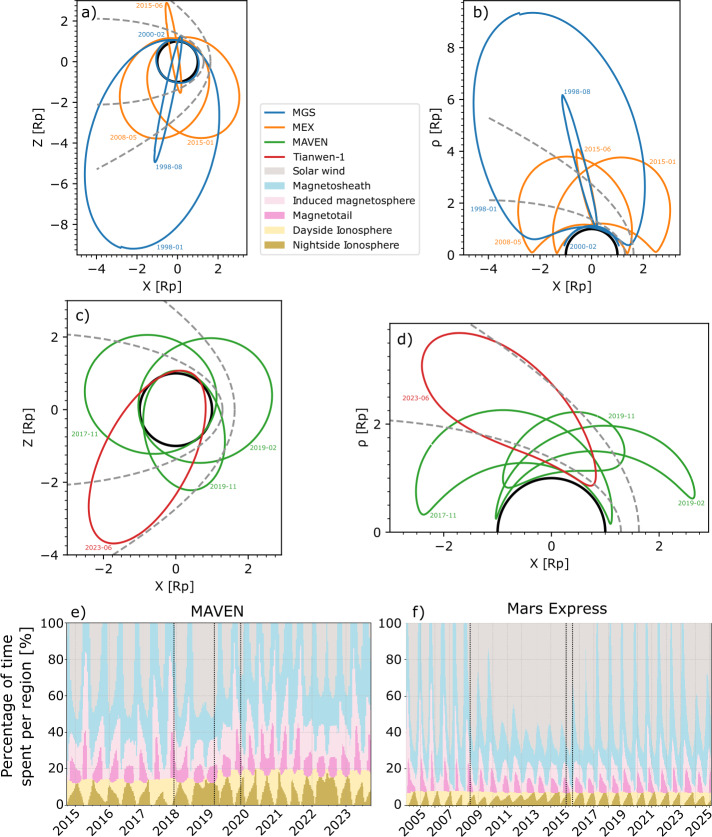
An overview of the orbit configurations for the main plasma missions to Mars. Panels (a) and (c) show the spacecraft trajectories in MSO X-Z coordinates, while panels (b) and (d) display the MSO X-ρ coordinates, where $\rho = \sqrt{Y^{2} + Z^{2}}$. Panels (a) and (b) illustrate the orbits of MGS and MEX, whereas (c) and (d) depict MAVEN and Tianwen-1. To highlight orbit precession, three example orbits are shown for all missions except Tianwen-1. Panels (e) and (f) present the percentage of time MAVEN and MEX spend in each plasma region over a 10-day interval, using a color scheme consistent with the schematic in Fig. 1. The ionosphere is defined as an altitude below 1000 km, and the magnetotail region is restricted to the nightside region of the magnetotail, within $\rho < 1~\mathrm{R}_{P}$ (planetary radius)

**Fig. 4:**
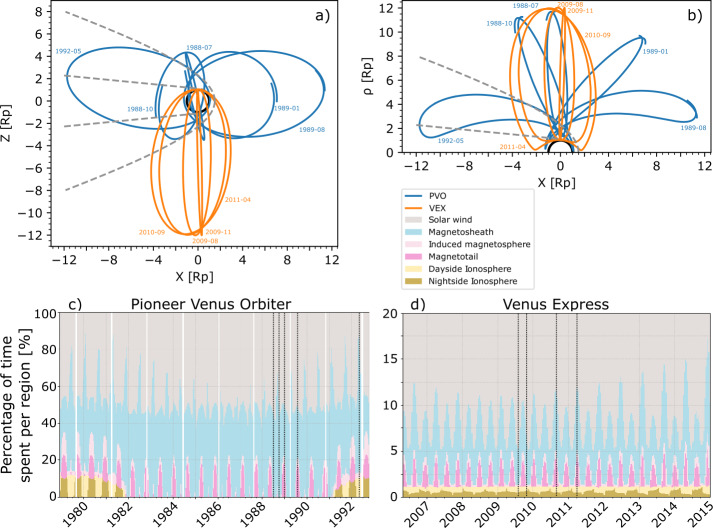
An overview of the orbit configurations for the main plasma missions to Venus: PVO and VEX. The Figure follows the same structure as Fig. 3, with five representative orbits from each mission shown in the Venus Solar Orbital (VSO) coordinate system to illustrate orbit precession over the Venusian year. Venus Express spent more than 80% of its time in the solar wind; therefore, for clarity, panel (d) is scaled to a maximum of 20%

In Figs. 3 and 4, we illustrate the orbit coverage and time spent in various plasma regions for recent missions to Mars and Venus that carried plasma instrumentation. The orbits are plotted in the Mars Solar Orbital (MSO) and the Venus Solar Orbital (VSO) coordinate systems, for Mars and Venus respectively, where the X axis points from the planet to the Sun, the Y axis points opposite to the planet’s orbital motion, and the Z axis completes the right-handed system. For Mars, the orbits of MAVEN, Mars Express, Tianwen-1 (Zou et al. 2021), and MGS are shown. At Venus, the focus is on PVO and VEX, the two primary plasma missions. The bottom two panels of both figures highlight the percentage of time spent in key plasma regions over a 10-day period, defined by the bow shock and boundary parameters from prior studies (e.g. Edberg et al. 2008; Signoles et al. 2023; Martinecz et al. 2009). The black vertical lines in Figs. 3e, 3f and 4c, 4d depict the time of the plotted orbits of MAVEN, MEX, PVO, and VEX, respectively.

From Figs. 3 and 4, several key insights regarding orbit coverage emerge. MAVEN’s orbit, with a periapsis well below 250 km, allows detailed measurements of the ionosphere and thermosphere, the key source region for planetary plasma in its induced magnetosphere. In contrast, Mars Express has a higher periapsis (above 250 km), accommodating remote sensing for ionospheric studies. MAVEN’s shorter orbital period and lower periapsis result in a periodic lack of solar wind observations, while Mars Express, with its slightly longer orbit with higher apoapsis, consistently transitions into the solar wind. However, none of the missions shown in Fig. 3 extends beyond about $4~\mathrm{R}_{M}$ into the induced magnetotail, leaving the magnetotail beyond that distance largely unexamined. Near-Mars plasma regions, including the nightside ionosphere and near magnetotail, are well-covered by both Mars Express and MAVEN. Overall, the missions offered complementary perspectives, but the orbit similarities among them resulted in gaps in the far tail and in the continuous upstream solar wind coverage.

At Venus, PVO and VEX exhibited distinct orbit characteristics. Both followed elliptical polar orbits with apoapses near $10~\mathrm{R}_{V}$, but PVO’s periapsis was in the equatorial plane, resulting in apoapses near the ecliptic plane. This enabled PVO to sample the far magnetotail during certain periods of the Venusian year and the near magnetotail and nightside ionosphere during others. VEX, in contrast, had a periapsis near Venus’s north pole and consistently sampled the near magnetotail (around $2~\mathrm{R}_{V}$). PVO’s low periapsis during its early mission phase allowed extensive ionospheric measurements, but later orbit adjustments raised it above 250 km. VEX maintained a nominal periapsis near 250 km, with additional campaigns lowering it for detailed atmospheric studies. These orbital differences, combined with different plasma instrumentation, make direct comparisons challenging, but highlight complementary strengths in Venus plasma studies.

**Fig. 5:**
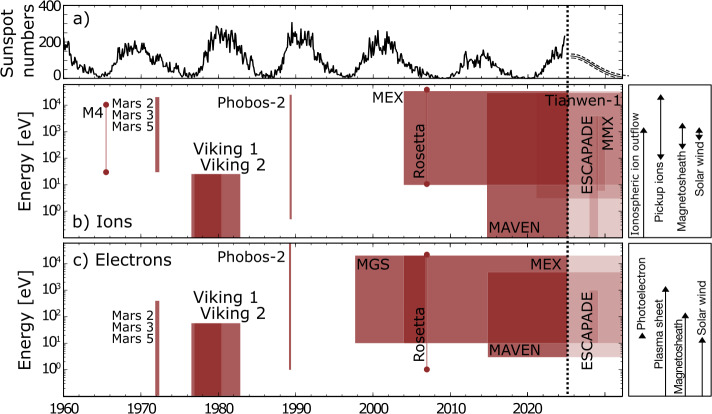
Overview of the ion and electron instruments onboard missions that have visited Mars, inspired by Fig. 2 of Futaana et al. (2017). a) Sunspot numbers, b) total energy ranges covered from all the instruments of each mission for ions and c) electrons. *Note*: The references for each instrument are provided in Table 1

**Fig. 6:**
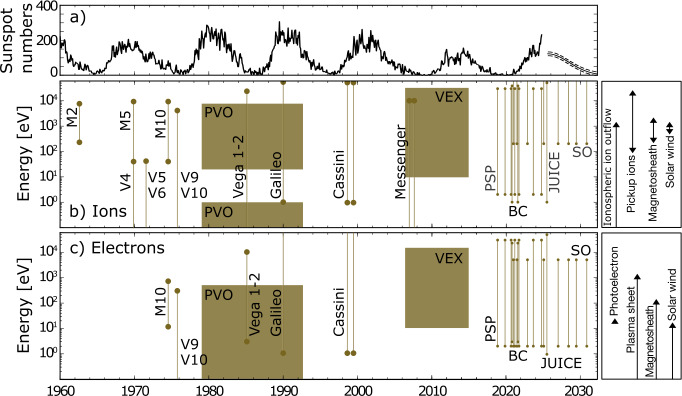
Similar to Fig. 5, now for instruments onboard missions to Venus. *Note*: The reference for each instrument is provided in Table 1 of Futaana et al. (2017) or in Table 2

Figures 5 and 6 provide an overview of the ion and electron instrumentation used on missions to Mars and Venus, respectively. In Fig. 5, the future extension periods of the currently active missions are shown to the right of the dotted vertical line in a lighter colour, based on the planning valid at the time of writing this article. These plans may be altered in the future. A detailed summary of plasma instruments is presented in Tables 1 and 2, inspired by Fig. 2 and Table 1 in Futaana et al. (2017). These resources have been extended to include recent missions with past and future planned flybys of Venus and ongoing missions at Mars. Several key insights into the evolution of plasma instrumentation and its contributions to our understanding of planetary environments emerge from these Tables and Figures.

**Table 1:** An overview of Mars missions with plasma instrumentation, including references to instrument papers. The Table structure is adapted from Table 1 of Futaana et al. (2017)

Mission	Measurement period	Type	Magnetic field	Ions	Electrons	Waves	Langmuir probe	Energetic particles	Energetic neutral atoms
Mariner 4	1965-07-15	F	1	2					
Mars 2	1971-11 to 1972-03	OP	3	4, 5	5				
Mars 3	1971-12 to 1972-03	OP	3	4, 5	5				
Mars 5	1974-02 to 1974-02	OP	3	4, 5	5				
Viking 1	1976-06-19 to 1982-11-11	OL		6	7				
Viking 2	1976-08-07 to 1980-04-12	OL		6					
Phobos 2	1989-01 to 1989-03-27	O	8, 9	10, 11, 12	10, 12	13	13	14	
Mars Global Surveyor	1997-09-12 to 2006-11-02	O	15		16				
Mars Odyssey	2001-10-24 to present	O						17	
Mars Express	2003-12-25 to present	O	18	19	18, 19				19
ROSETTA	2007-02-25	F	20	21, 22, 23	22, 24	24	25		
MAVEN	2014-09-21 to 2025-12-06	O	26	27, 28, 29	30	31	31	32	
Tianwen-1	2021-02-10 to present	OR	33	34				35	34

Types: F: Flyby, O: Orbiter, P: Probe, L: Lander, R: Rover. The numbers specify the references for the instrument description.

1: Coleman et al. (1966) 2: Bridge et al. (1965) 3: Dolginov et al. (1976) 4: Ainbund et al. (1973) 5: Gringauz et al. (1974) 6: Hanson et al. (1977) 7: Johnson and Hanson (1991) 8: Riedler et al. (1989) 9: Riedler et al. (1991) 10: Lundin et al. (1989) 11: Rosenbauer et al. (1989) 12: Kiraly et al. (1991) 13: Grard et al. (1989) 14: McKenna-Lawlor et al. (1992) 15: Acuña et al. (1998) 16: Mitchell et al. (2001) 17: Badhwar (2004) 18: Jordan et al. (2009) 19: Barabash et al. (2006) 20: Glassmeier et al. (2007) 21: Nilsson et al. (2007) 22: Burch et al. (2007) 23: Balsiger et al. (2007) 24: Trotignon et al. (2007) 25: Eriksson et al. (2007) 26: Connerney et al. (2015) 27: Halekas et al. (2015) 28: McFadden et al. (2015) 29: Mahaffy et al. (2015) 30: Mitchell et al. (2016) 31: Andersson et al. (2015) 32: Larson et al. (2015) 33: Liu et al. (2020) 34: Kong et al. (2020) 35: Tang et al. (2020)

**Table 2:** An overview of Venus missions with plasma instrumentation, including references to instrument papers. The Table structure is similar to Table 1. The portion up to Venus Express is adapted from Table 1 of Futaana et al. (2017), excluding missions without plasma instrumentation, and has been extended to include recent missions with Venus flybys

Mission	Measurement period	Type	Magnetic field	Ions	Electrons	Waves	Langmuir probe	Energetic particles	Energetic neutral atoms
Mariner 2	1962-12-14	F	1	1				1	
Venera 4	1967-10-18	P	2	3					
Mariner 5	1967-10-19	F	4	5				5	
Venera 5, 6	1969-05-16, 17	P		6					
Mariner 10	1974-02-05	F	7	7	7			7	
Venera 9, 10	1975-10-22, 25	OL	8	9, 10	9			11	
Pioneer Venus	1978-12-04 to 1992-10	OP	12	13–15	13, 14	16	17		
Vega 1,2	1985-06-11	FLB	18	19	19	20,21	22, 23	24	
Galileo	1990-02-10	F	25	26	26	27		28	
Cassini	1998-04-26, 1999-06-24	F	29	30, 31	31	32	32	33	33
MESSENGER	2006-10-24, 2007-06-05	F	34	35				35	
Venus Express	2006-04-16 to 2014-11	O	36	37	37				37
Solar Orbiter	2020-12-27 to 2030-09-02 (planned)	F(8)	38	39	39	40		41	
Parker Solar Probe	2018-10-03 to 2024-11-06	F(7)	42	43	43	42		44	
BepiColombo	2020-10-15, 2021-08-10	F(2)	45, 46	47, 48	47	49		47	47

Types: F: Flyby, P: Probe, L: Lander, O: Orbiter, B: Balloon. Numbers in parentheses indicate number of (planned) flybys, and numbers in each category specify the references for the instrument description.

1: NASA (1965) 2: https://nssdc.gsfc.nasa.gov/nmc/experiment/display.action?id=1967-058A-01 3: Verigin et al. (1978) 4: Connor (1968) 5: https://nssdc.gsfc.nasa.gov/nmc/experiment/display.action?id=1967-060A-04 6: https://nssdc.gsfc.nasa.gov/nmc/spacecraft/display.action?id=1969-001A# 7: Bridge et al. (1976); https://nssdc.gsfc.nasa.gov/nmc/experiment/display.action?id=1973-085A-03 8: Keldysh (1977) 9: Gringauz et al. (1976) 10: Vaisberg et al. (1976) 11: https://pds.mcp.nasa.gov/portal/instruments/urn--nasa--pds--context--instrument--v9---ep/overview 12: Russell et al. (1980b) 13: Intriligator et al. (1980) 14: Knudsen et al. (1980) 15: Taylor et al. (1980) 16: Scarf et al. (1980b) 17: Krehbiel et al. (1980) 18: Reidler et al. (1986) 19: Gringauz et al. (1986) 20: https://nssdc.gsfc.nasa.gov/nmc/experiment/display.action?id=1984-125A-10 21: https://nssdc.gsfc.nasa.gov/nmc/experiment/display.action?id=1984-128A-10 22: https://nssdc.gsfc.nasa.gov/nmc/experiment/display.action?id=1984-125A-11 23: https://nssdc.gsfc.nasa.gov/nmc/experiment/display.action?id=1984-128A-11 24: Somogyi et al. (1986) 25: Kivelson et al. (1992) 26: Frank et al. (1992) 27: Gurnett et al. (1992) 28: Williams et al. (1992) 29: Dougherty et al. (2004) 30: Waite et al. (2004) 31: Young et al. (2004) 32: Gurnett et al. (2004) 33: Krimigis et al. (2004) 34: Anderson et al. (2007) 35: Andrews et al. (2007) 36: Zhang et al. (2007) 37: Barabash et al. (2007b) 38: Horbury et al. (2020) 39: Owen et al. (2020) 40: Maksimovic et al. (2020) 41: Rodríguez-Pacheco et al. (2020) 42: Bale et al. (2016) 43: Kasper et al. (2016) 44: McComas et al. (2016) 45: Heyner et al. (2021) 46: Baumjohann et al. (2020) 47: Saito et al. (2021) 48: Orsini et al. (2021) 49: Kasaba et al. (2020)

The MGS mission, though not equipped with a comprehensive plasma suite, provided groundbreaking insights into the crustal magnetic fields of Mars using its magnetometer and electron spectrometer. These measurements laid the foundation for understanding the interaction between the solar wind and localized Martian magnetic anomalies. Mars Express, with its well-equipped set of plasma particle instruments, has made extensive contributions to studies of the ionosphere, ion escape, and plasma boundaries -just a few examples of its broader impact on Mars plasma environment. However, the lack of an onboard magnetometer created a significant gap, limiting the ability to correlate particle data with magnetic field measurements. This shortcoming hindered a complete understanding of the underlying plasma processes and their spatial and temporal variations. MAVEN, on the other hand, with its payload combining particle instruments with a magnetometer, has advanced our understanding of -among other things- the tail magnetic structure, magnetic topology, the influence of the crustal fields on the ionosphere, and to some extent the response of the Martian hybrid magnetosphere to solar wind and space weather drivers. MAVEN stands out as the only mission capable of measuring ions at the lowest energy levels close to escape energy, and also the full ion distribution function (energy, direction, mass) at a relatively high cadence (4 s, whereas MEX’s ion mass analyzer-IMA makes ion distribution measurements at a 192-s cadence), which allows us to resolve a significant portion of the kinetic physics at play. The outflow of the low-energy ions plays a significant role in the Martian atmospheric evolution, particularly in the context of long-term escape to space.

At Venus, the PVO and VEX missions provided valuable but distinct perspectives, owing to their vastly different instrumentation. These differences make direct comparisons challenging. For instance, the energy ranges and sensitivities of the instruments on PVO and VEX were not directly compatible, and the low time resolution of VEX’s plasma particle instruments has left many questions unanswered regarding the detailed plasma characteristics. Despite these challenges, both missions significantly advanced our understanding of Venus’s plasma environment by addressing distinct observational niches. PVO characterized the structure of the Venusian induced magnetosphere, identified its plasma boundaries, and provided early evidence for ion escape. It also contributed to defining the plasma boundaries around the planet and provided early evidence of ion escape. VEX provided plasma observations of better spatial and temporal resolution. Quantitative estimates of the ion escape rates were made possible, as well as the investigation of ion escape drivers. A hot oxygen corona was detected and new insights were gained into wave activity around the planet.

By integrating insights from these missions and leveraging the complementary strengths of their instrumentation, we now better understand the plasma environments of Mars and Venus. However, the gaps and limitations in past measurements highlight the need for future missions that will probe i) unexplored regions, such as low latitudes and altitudes at Venus, and the mid and far magnetotails of Venus and Mars and ii) unresolved processes in regions already explored. To address unresolved questions, comprehensive, multi-instrument payloads and multi-point measurements are needed.

## Boundaries

In this section we discuss the overall structure of the induced magnetospheres of Mars and Venus to give a complete picture of their planetary plasma environment, part of which are the magnetotails. We describe the main boundaries determined by the solar wind interaction with the planetary atmospheres, while detailed discussion of the tail structure is left for the later sections. Schematics of these induced magnetospheres are given in Fig. 1.

### Bow Shock

The bow shock is the outermost boundary of any magnetosphere, since it represents the location where solar wind plasma is suddenly slowed. This shock then extends downstream of the planet and at Venus has been seen to distances of 60 R_V_ (Edberg et al. 2024a; Stergiopoulou et al. 2023), while at Mars it has been measured downstream of the planet at a distance of 20 R_M_ (Edberg et al. 2009b). As the bow shock location depends on the magnetosonic Mach number, it can only be detected at distances where the magnetosonic waves are damped to a level which is comparable to the average Alfvénic wave amplitude in the solar wind. Note that this does not mean that the bow shock extends further at Venus, simply, thanks to recent flybys of Venus, we have observations at larger distances downtail.

The average position of the bow shock and models of the overall shape have been established through a number of studies at both Mars (Edberg et al. 2009b; Trotignon et al. 2007; Hall et al. 2016; Gruesbeck et al. 2018), and Venus (e.g. Russell et al. 1988; Martinecz et al. 2009; Shan et al. 2015). Hall et al. (2019) demonstrated that, at Mars, the average position of the bow shock at the terminator, $R_{TD}$, increased from a minimum of $2.336 \pm 6 \cdot 10^{-3}~\mathrm{R}_{M}$ in Martian Year (MY) 28 (solar minimum) to $2.507 \pm 8 \cdot 10^{-3}~\mathrm{R}_{M}$ in MY 32 (solar maximium), with the average position of $R_{TD}$ being $2.48~\mathrm{R}_{M}$. Furthermore, an earlier study by Hall et al. (2016) showed that the average position increased by a similar amount from Mars aphelion, $2.39 \pm 10^{-2}~\mathrm{R}_{M}$, to Mars perihelion, $2.65 \pm 2 \cdot 10^{-2}~\mathrm{R}_{M}$. These authors suggested that this was due to the increased solar EUV radiation. Simon Wedlund et al. (2022) developed an algorithm to automatically locate the bow shock position in spacecraft orbits, which also allowed the authors to investigate the variation of the bow shock with Martian years, Martian seasons and solar EUV flux. The variability of the Martian’s bow shock location was also studied by Garnier et al. (2022), who concluded that the bow shock’s location mainly depends on the EUV flux and the magnetosonic Mach number, while the solar wind dynamic pressure, the crustal fields and the IMF orientation affect its location to a lesser degree.

Similar studies of the variability of the bow shock at Venus indicate similar responses. For example, the distance from the planet increases with increasing solar EUV radiation, (e.g. Shan et al. 2015), while the distance also increases from solar minimum to solar maximum (Signoles et al. 2023). Furthermore, there is clear evidence of the distance from the planet increasing with the IMF magnitude (Signoles et al. 2023), as well as magnetosonic and Alfvén Mach numbers (Zhang et al. 2008; Signoles et al. 2023). The BS is further from the planet above the poles than at the equator, but at Mars the presence of the crustal magnetic fields does result in the BS being further from the planet than at other locations, i.e the BS is not symmetric (Edberg et al. 2008; Gruesbeck et al. 2018).

### Magnetic Pile-up Boundary

On passing across the shock, solar wind plasma with the “frozen-in” IMF decelerates, resulting in the magnetic field being “piled up”, creating a region on the dayside referred to as the magnetic pile-up region (Bertucci et al. 2005). This results in enhanced magnetic field but slower plasma. The slowed solar wind and IMF also create a magnetosheath, the inner boundary of which can be described as the magnetic pile-up boundary, where the magnetic field reaches a maximum, before falling off quickly closer to the planet. A separate boundary, the ion composition boundary, ICB, (Martinecz et al. 2008; Halekas et al. 2018) is also formed as this represents the boundary between solar wind plasma and planetary plasma, based on measurements from ion instruments. Studies at Venus tend to focus on the latter while at Mars both boundaries are often considered, although they are not necessarily co-located. The MPB or ICB can be considered to be the outermost part of the induced magnetosphere and can also be referred to as the induced magnetosphere boundary (Holmberg et al. 2019). We note that in the cases where more than one term is used to refer to either certain boundaries or regions, their definitions depend on the observables that are used in each case (Espley 2018).

The variability of the MPB at Mars largely follows that of the solar wind dynamic pressure, albeit with a smaller level of change. Critically, there is clear evidence of the impact of the crustal fields (Edberg et al. 2008; Crider et al. 2002). Strong crustal fields tend to place the MPB about $0.1~\mathrm{R}_{M}$ further from the planet. At Venus, the distance of the ICB appears to decrease with increasing dynamic pressure and IMF magnitude (Signoles et al. 2023), although some studies have suggested that again the ICB altitude variation with upstream conditions is small, especially at solar minimum (e.g. Luhmann 1991; Angsmann et al. 2011).

### Ionosphere and Ionopause

Below the MPB lies the induced magnetosphere and the plasma on the dayside is now of thermal energies, as the main source of plasma is the ionosphere, which is primarily composed of heavier, planetary ions. The ionosphere is the atmospheric layer that is photoionized by Solar EUV and soft X-ray radiation and is in photochemical equilibrium. The shape of the main ionospheric layers at Mars and Venus can be described in terms of simple Chapman theory (Schunk and Nagy 2009; Sánchez-Cano et al. 2013), which assumes that the global atmosphere is in hydrostatic balance and in photochemical equilibrium, the incoming radiation is monochromatic and each photon produces a single electron, the atmospheric layers are horizontally stratified, electrically neutral, consist of a homogeneous gas formed by a single component, and remain in equilibrium, and it assumes that only one ion species is present (Chapman 1931). However, Chapman theory cannot fully describe the complexity of the entire ionospheres of the two planets. The ionospheres of both Mars and Venus are in general similar, consisting mainly of $O_{2}^{+}$ below the exobase, and $O^{+}$ above. Two main layers exist in each ionosphere, both described by Chapman theory, with peak electron densities of ∼$1.2\times 10^{11}\text{ m} ^{-3}$ at ∼140 km near the subsolar point and at ∼$10^{10}\text{ m}^{-3}$ at ∼110 km. Mars’s low thermal pressure leads to an uncompensated balance with the solar wind, resulting in its ionosphere being magnetized for most of the time. In contrast, Venus’s ionosphere becomes magnetized mainly during periods of low solar activity (Luhmann et al. 1991; Sánchez-Cano et al. 2020), however, it has been shown that the magnetization of the Venusian ionosphere is complex and depends on the IMF (Angsmann et al. 2011; Chang et al. 2020). Additionally, Mars’s elliptical orbit about the Sun plays a significant role in modulating the total electron content of its ionosphere (Sánchez-Cano et al. 2016). This in turn, affects the location of all the plasma boundaries of the system, particularly the bow shock as demonstrated by Hall et al. (2016, 2019). This EUV-driven control of plasma boundaries is markedly different from the solar wind interaction with magnetized bodies such as Earth, where the solar wind dynamic and magnetic pressures control the interaction with minimal influence from the EUV flux.

For unmagnetized planets such as Mars and Venus, the uppermost region of the ionosphere is the ionopause. At Venus during solar maximum, the ionopause is very well-behaved in the sense that there is a clear balance between magnetic and thermal pressures within this boundary and the thermal pressure of the ionosphere holds off the magnetic pressure, which in turn balances the dynamic pressure of the incident solar wind (Luhmann 1986; Luhmann et al. 1987). When the ionospheric pressure is insufficient to withstand the solar wind dynamic pressure, an induced magnetic field is typically present in the ionosphere, and the ionopause then is less well defined and forms at lower altitudes (Elphic et al. 1980; Phillips et al. 1985; Luhmann et al. 1987). At Mars, the ionopause is magnetized most of the time, and there is never a balance between magnetic and thermal pressures within this boundary. Rather, this balance is found much deeper, closer to the peak of the ionosphere. Dubinin et al. (2008) have shown that since the solar wind dynamic pressure converts to thermal pressure in the magnetosheath, the ionopause may be defined by the location of pressure balance between the proton thermal pressure and the sum of the magnetic plus the heavy ion thermal pressure. This can also more simply be interpreted as the pressure balance between the solar wind dynamic pressure and the total pressure of the ionosphere (magnetic plus thermal pressures) (Sánchez-Cano et al. 2020; Chu et al. 2021). The ionopause at Mars is observed roughly half of the time, and when present, it is located over a wide range of altitudes, varies rapidly, and is highly structured (e.g. Chu et al. 2019; Duru et al. 2020; Vogt et al. 2015; Stergiopoulou et al. 2025). Stergiopoulou et al. (2025) have shown that the Martian ionopause is formed at or near the location where the closed magnetic topology switches to either open or draped magnetic field lines. In most of the cases the authors examined, the ionopause was also found to form where there is pressure balance between the thermal pressures below and above the boundary.

Both the Venusian and Martian ionospheres exhibit exceptional variability in their profiles. For example, sporadic layers can appear at different altitudes from the main layers in both ionospheres (e.g. Pätzold et al. 2005; Peter et al. 2024; Crismani et al. 2017; Tripathi et al. 2024), while both respond similarly to the impact of coronal mass ejections (CMEs), with a general compression of the topside ionosphere, a typically lower altitude ionopause, and a larger induced magnetic field present at high altitude. A very important difference between the two ionospheres, however, is the response to solar energetic particle (SEP) events, which are known to create long-lived ionospheric layers between 50 and 100 km altitude at Mars (e.g. Sánchez-Cano et al. 2019; Lester et al. 2022), but as yet has not been fully investigated at Venus, due to lack of observations. Tripathi et al. (2024) have shown that while SEPs may contribute to the formation of lower ionospheric layers at Venus, they are not a main driving force in the Venusian ionosphere, unlike at Mars, where they play a dominant role.

The nightside ionosphere is very patchy and sporadic. The two main layers of the dayside ionosphere disappear as soon as solar photoionization disappears with the night. The ionosphere is mainly maintained on the nightside due to processes such as transport and particle precipitation. While at Venus transport dominates, at Mars, particle precipitation is a major source of ionization over the entire nightside. The main reason is the magnetic reconnection of the crustal fields with the IMF that creates complex magnetic topology on the nightside (e.g. Xu et al. 2019; Sarkar et al. 2025). This gives rise to a number of phenomena such as an ionospheric enhancement over near vertical crustal field lines (Cartacci et al. 2013) or auroral processes that also produce enhancements in the ionosphere (e.g. Harada et al. 2024; Lester et al. 2022; Lillis et al. 2024).

The magnetotail at both Mars and Venus is formed primarily as a result of the draped IMF about the planet. As this forms the basis of the remainder of the paper, we do not discuss this further here. We do note, however, that the presence of crustal magnetic fields at Mars does lead to some level of complexity for the Martian tail. Further we note that the distance downtail may lead to significant differences in tail structure. While we do not precisely define what is meant by “near” and “far” tail, it should be emphasized that the behavior of the tail and its response to either solar wind inputs or solar EUV forcing may differ depending on the distance from the planet.

### Simulations

**Fig. 7:**
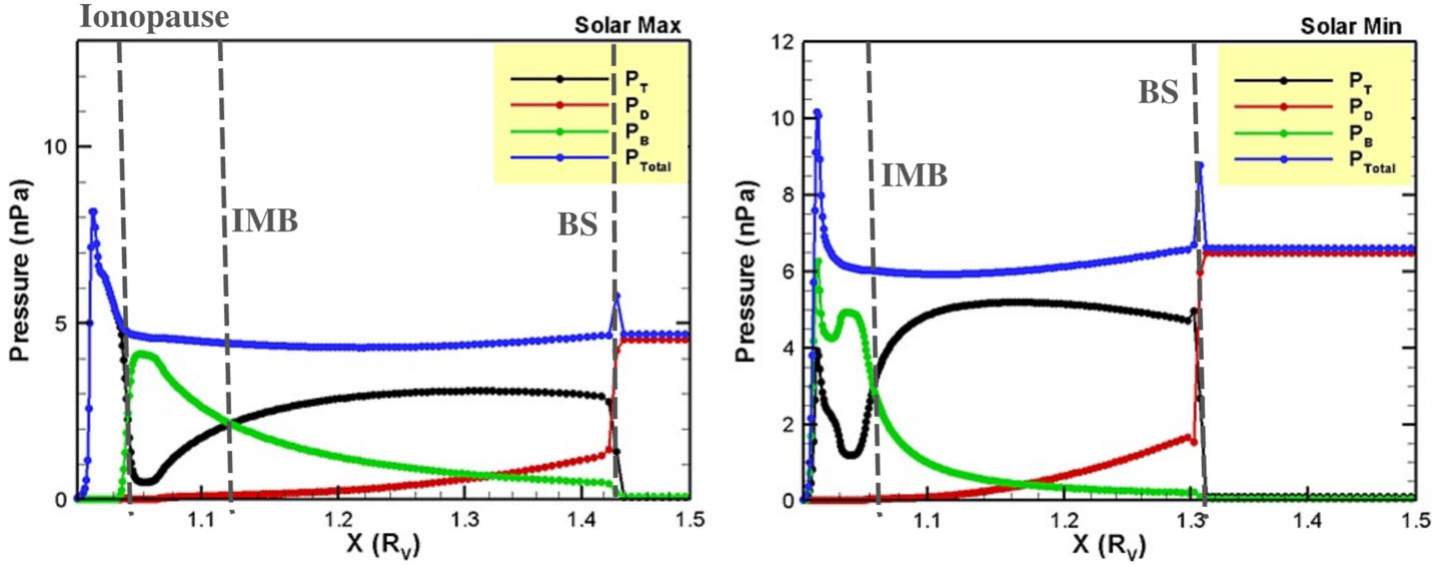
Pressure profiles as functions of distances along the subsolar line for solar maximum and solar minimum cases at Venus. Reproduced from Fig. 5 of (Ma et al. 2013). Copyright 2013 by AGU, reproduced with permission

Observations are often compared with global simulations that describe the large-scale context of the system. Such models are typically based on magnetohydrodynamics (MHD) or hybrid approaches in the outer regions, whereas in the ionosphere, these models focus more on the interaction between plasma and the neutral atmosphere. The boundaries in global simulations are identified using several parameters such as the magnetic field, electric field, plasma density and velocity, and pressure. Technically, this does not differ in different models. For example, in MHD models, the plasma boundaries are determined based on the relative significance of various pressures. Figure 7 illustrates the pressure profiles along the sub-solar line for Venus under solar maximum and solar minimum conditions. In global MHD and multispecies MHD simulations, the bow shock is characterized by a sharp decrease in plasma dynamic pressure together with increases in plasma thermal and magnetic pressures (e.g Bauske et al. 1998; Kallio et al. 1998; Terada et al. 2009; Ma et al. 2013, 2020, 2025; Dang et al. 2023). It is also important to note that MHD models do not resolve the physical shock width; instead, the modeled shock width is determined by the grid resolution.

**Fig. 8:**
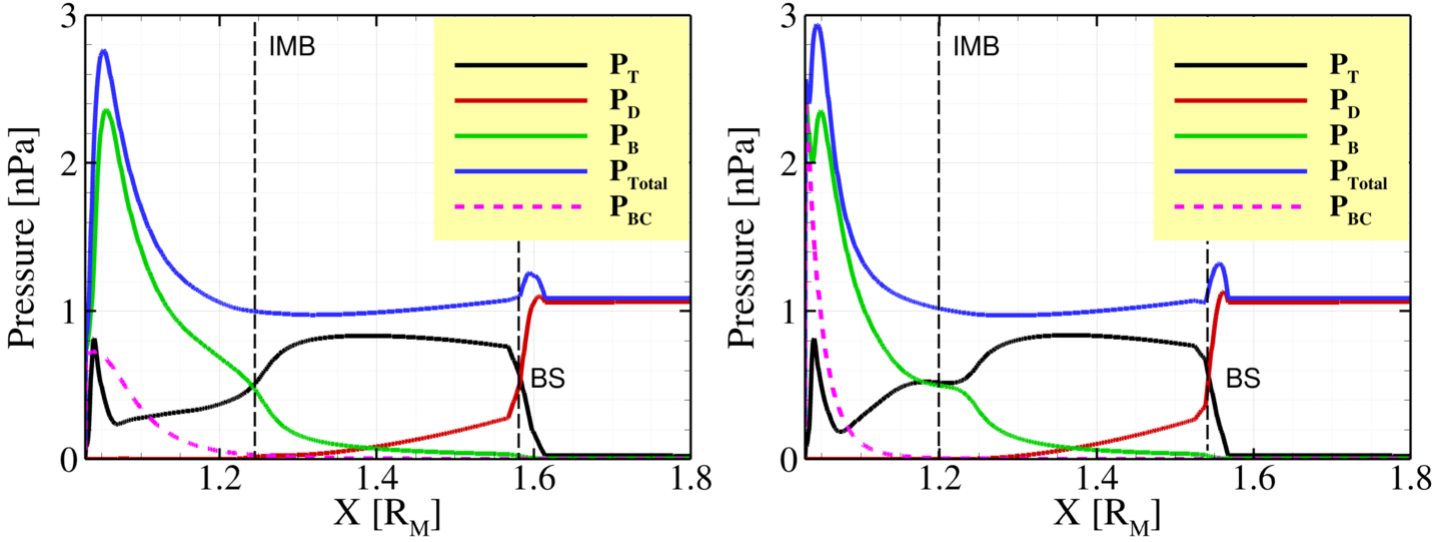
Pressure profiles as functions of distances along the subsolar line at Mars, based on multi-species MHD model results of case 1 (left), where the strong crustal fields face the Sun and case 2 (right), where the strong crustal fields are on the nightside. Based on the results of the study by Ma et al. (2019)

Then, closer to the planet, the IMB, or MPB, is identified as the location where the plasma thermal pressure equals the magnetic pressure. The ICB is the boundary defined where the densities of planetary ions are equal to the densities of protons, which come primarily from the solar wind. In the MHD model, a more precise method is to distinguish solar wind protons from planetary protons which is easier with the multi-fluid/species MHD and hybrid/particle-in-cell (PIC) simulations. The innermost boundary can be the ionopause, which is not always present. It becomes well-defined when the ionospheric thermal pressure exceeds the solar wind dynamic pressure, typical for the solar maximum case (as shown in the left panel of Fig. 7, with a sharp decrease of the magnetic field) for Venus or when the solar wind dynamic pressure is relatively low. The situation is more complicated at Mars because crustal magnetic fields strongly modify boundary locations, topology, and pressure balance, as demonstrated in global MHD and multifluid MHD studies that include crustal-field effects (e.g Ma et al. 2004, 2014, 2019; Najib et al. 2011; Dong et al. 2014, 2015b; Song et al. 2023; Sun et al. 2023; Sakata et al. 2024) and MAVEN observations (e.g Matsunaga et al. 2017; Xu et al. 2016a, 2023c; Fang et al. 2017). Figure 8 illustrates this effect: The left panel corresponds to a case where the strong crustal fields face the Sun (subsolar longitude of 180 degrees), whereas the right panel represents a scenario where the strong crustal field region is on the nightside (subsolar longitude of 0 degrees).

On the other hand, hybrid simulations treat ions as particles and electrons as a charge-neutral mass-less fluid (while in MHD the plasma is treated as a fluid). This difference allows us to distinguish between different ion species and their origins. Several global hybrid simulations incorporate ions from both the solar wind and the planetary ionosphere, (e.g. Terada et al. 2002; Modolo et al. 2012; Jarvinen et al. 2020; Kallio et al. 2006, 2010; Jarvinen et al. 2013, 2018, 2022; Fatemi et al. 2017; Wang et al. 2023c, 2024b), enabling us to better understand, for example, atmospheric escape and the respective contributions of each source.

**Fig. 9:**
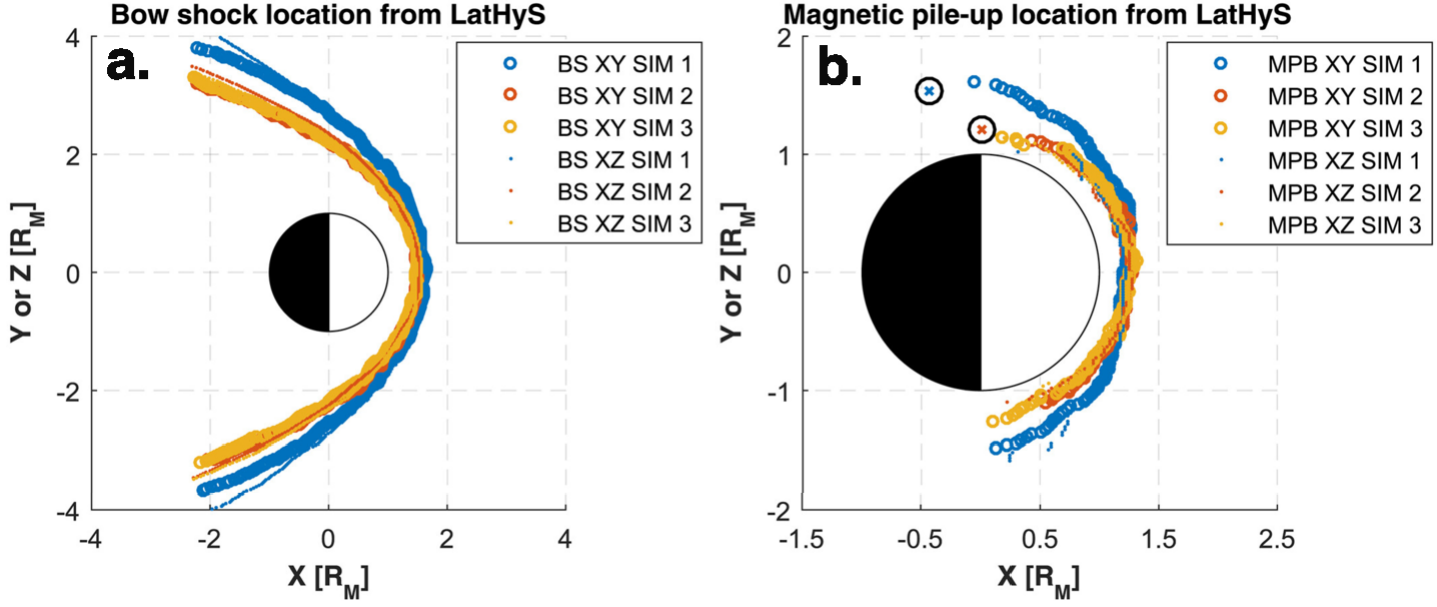
Hybrid simulations (LatHyS) of the Martian magnetosphere’s response to the ICME that arrived at Mars on September 13, 2017, presented on the XY-XZ MSO planes. Reproduced from Fig. 2 of Romanelli et al. (2018b). Copyright 2018 by AGU, reproduced with permission

Similar definitions and identification techniques have also been applied in relation to hybrid model simulation results for both nominal conditions around Mars and Venus, as well as solar extreme events (e.g. Modolo et al. 2012; Romanelli et al. 2018b). Figure 9 displays the results of three hybrid stationary simulations used to analyze the Martian magnetosphere’s response to an interplanetary coronal mass ejection (ICME) that impacted Mars on September 13, 2017 (Romanelli et al. 2018b). In particular, these simulations allow us to characterize how variations in the solar wind magnetosonic Mach number and dynamic pressure influence the locations of the bow shock and magnetic pile-up boundary. Note that all simulations were conducted under the same solar extreme ultraviolet conditions and with an IMF primarily oriented along the Y-MSO axis (see Table 1 of Romanelli et al. (2018b)). The criteria for the identification of the bow shock and magnetic pile-up boundary are based on jump conditions for different variables, such as the magnetic field magnitude and solar wind plasma velocity, and pressure balance considerations. The identified bow shock and magnetic pile up position in the XY and XZ MSO planes for each of the three simulation runs are shown in Fig. 9. Among other results, the authors reported that the Laboratoire Atmosphères, Milieux et Observations Spatiales Hybrid Simulations (LatHyS) show that the bow shock is closer to Mars under conditions of higher magnetosonic Mach number and increased solar wind dynamic pressure, consistent with previous observational studies. Additionally, both LatHyS results and MAVEN observations indicate a compression of the MPB along its flanks.

## Tail Structure and Physical Processes: Mars vs Venus

### Basic Picture: Draping and Variability with the IMF

The magnetotail of Venus is the result of the direct interaction between the magnetized solar wind and the planet’s highly conductive ionosphere. To first order, this interaction generates ionospheric induced currents that slow down and divert the solar wind plasma around Venus. Due to the frozen-in condition, the IMF piles-up and drapes around Venus (e.g. Luhmann 1986; Phillips and Russell 1987; Luhmann et al. 1991). As a result, the structure of Venus’s magnetotail is characterized by draped IMF lines. This leads to an induced magnetotail composed of two lobes of opposite polarity, separated by a magnetotail current sheet nominally located in the X-Z plane, in contrast to intrinsic planetary magnetospheres, where the tail current sheet is in the X-Y plane, as the dipole axis often lies primarily in the z-direction. Here, the X/Y/Z axes refer to the solar electric frame, where X is opposite to the solar wind flow, Y aligns with the component of IMF perpendicular to X, and Z completes the right-handed system. Therefore, the magnetotail lobes and current sheet orientation at Venus are strongly dependent on the IMF (e.g. Saunders and Russell 1986; Russell et al. 2006; Zhang et al. 2010; Dubinin et al. 2014; Dubinin and Fraenz 2015).

Similarly to Venus, the interaction between the solar wind and Mars’s ionosphere leads to the generation of induced currents that contribute to the formation of the Martian magnetosphere (e.g. Yeroshenko et al. 1990; Dubinin et al. 1993; Crider et al. 2004; Brain et al. 2006b; Dubinin et al. 2019; Azari et al. 2023; Zhang et al. 2022; Dubinin and Fraenz 2015). In addition, Mars also has crustal magnetic fields distributed across its surface, with the most intense sources located in the southern hemisphere near $180^{\circ}$ longitude (Acuña et al. 1998, 1999; Connerney et al. 2005). These remanent magnetic fields rotate with the planet, introducing and affecting various physical processes, and ultimately creating a highly dynamic environment around Mars (Crider et al. 2004; Nagy et al. 2004). As a result, the Martian magnetotail possesses a complex magnetic field topology where crustal magnetic fields (Acuña et al. 1998; Connerney et al. 2005) and draped IMF coexist (e.g. Crider et al. 2004; Yeroshenko et al. 1990; Romanelli et al. 2015; Azari et al. 2023; Zhang et al. 2022). This results in a hybrid magnetosphere that exhibits signatures present in both intrinsic and induced planetary magnetospheres (e.g. Luhmann et al. 2015; Dubinin et al. 2023; DiBraccio et al. 2018, 2022; Xu et al. 2023b).

**Fig. 10:**
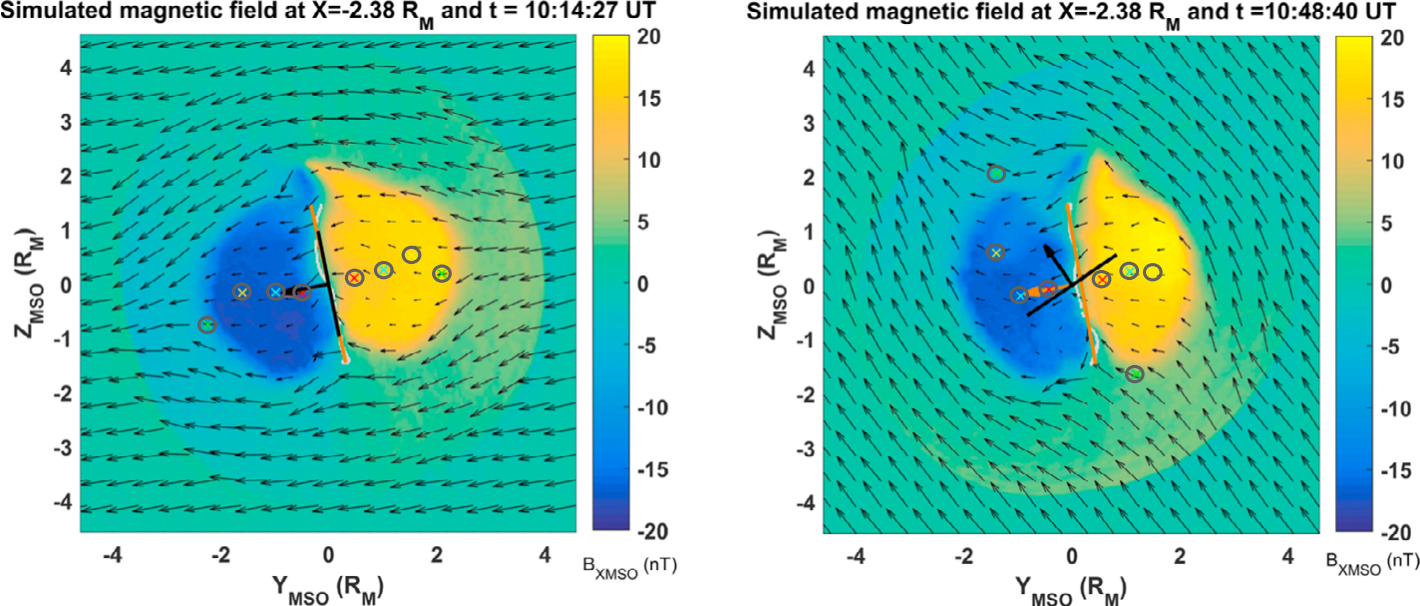
Two different states of the Martian magnetotail, based on a hybrid time-dependent numerical simulation. Both panels display the $\mathrm{Bx}_{\mathrm{MSO}}$ component (color-coded) at $X_{\mathrm{MSO}} = -2.38~\mathrm{R}_{M}$ and the magnetic field component perpendicular to the $X_{\mathrm{MSO}}$ axis (black arrows). The thick orange and black arrows represent the estimated normal to the neutral current sheet and the local mean IMF perpendicular component, respectively. Reproduced from Fig. 8 of Romanelli et al. (2018a). Copyright 2018 by AGU, reproduced with permission

The Venusian and Martian magnetospheres are affected by time-dependent processes. In particular, these environments are strongly influenced by temporal variability in the solar wind properties and solar radiation fluxes (e.g. Edberg et al. 2010a; Dubinin et al. 2011; Dong et al. 2015c; Halekas 2017). Temporal variability in the IMF causes variation in the orientation of the bow shock (both quasi-parallel and quasi-perpendicular regions), the magnetic pile-up boundary, and the magnetic field morphology within these planetary magnetospheres on different timescales (e.g. Modolo et al. 2012; Mazelle et al. 2004). IMF temporal variability also affects the location of the planetward and tailward lobes, the orientation of the magnetotail current sheet, and the location and extent of magnetotail asymmetries, among other effects (e.g. Saunders and Russell 1986; Slavin et al. 1989; Zhang et al. 2010; Dubinin and Fraenz 2015; Delva et al. 2017; Azari et al. 2023). The nature of the IMF variability also defines the magnetotail reconfiguration processes (e.g. Modolo et al. 2012). Romanelli et al. (2018a, 2019) studied the responses of the Martian magnetosphere to variability in the IMF clock angle, based on hybrid numerical simulations and MAVEN magnetic field and plasma observations (Modolo et al. 2016; Jakosky et al. 2015a). Figure 10 shows the simulated $\mathrm{Bx}_{\mathrm{MSO}}$ component at $X=-2.38~\mathrm{R}_{M}$, for two different states of the Martian magnetosphere derived from a time-dependent hybrid simulation. The left panel displays the magnetotail cross section, where the lobes and current sheet location and orientation are consistent with the draping of the IMF (e.g. Crider et al. 2004; Romanelli et al. 2015). For instance, the normal to the current sheet is approximately parallel to the IMF component that is perpendicular to the upstream solar wind velocity (shown by the black vectors upstream of the bow shock). In contrast, the right panel shows the same cross-section at a time where the magnetotail lobes have not adapted to the latest IMF configuration, after the clock angle change took place at approximately 10:47:30 UT. In particular, the normal to the current sheet (orange vector) is strongly tilted (${\sim} 60^{\circ}$) with respect to the IMF (black vector). The authors concluded that different regions inside the Martian magnetosphere adapt over different timescales to a given IMF clock angle change, e.g., ∼10 minutes for the magnetotail lobes ($X<-2.4~\mathrm{R}_{M}$).

Observations and numerical simulations of the Venusian magnetosphere suggest that the recovery timescales of the magnetotail are similar. Slavin et al. (2009) have shown that changes in the IMF orientation affect the magnetotail of Venus between the terminator plane and $3~\mathrm{R}_{V}$ downtail in about 8 minutes, based on simultaneous Venus Express and MESSENGER magnetic field measurements. Numerical simulation efforts for the observed solar wind conditions are in agreement with these results. Benna et al. (2009) performed four stationary MHD simulations and concluded that the magnetosphere of Venus adapts within a few minutes to changes in IMF. In addition, recent time-dependent MHD simulations suggest that the dayside magnetosphere reacts to IMF variability on timescales ranging from 10 seconds to 10 minutes, depending on the magnetospheric region (Xu et al. 2022a). The authors also concluded that the recovery time for the Venusian magnetotail, in response to an IMF rotation, is estimated to range from approximately 10 to 20 minutes. Despite these results, further analysis is needed to better understand the recovery processes and IMF transport into the Martian and Venusian magnetotails and how they vary with the solar wind and planetary properties.

### Crustal Magnetic Fields and the Hybrid Magnetosphere of Mars

**Fig. 11:**
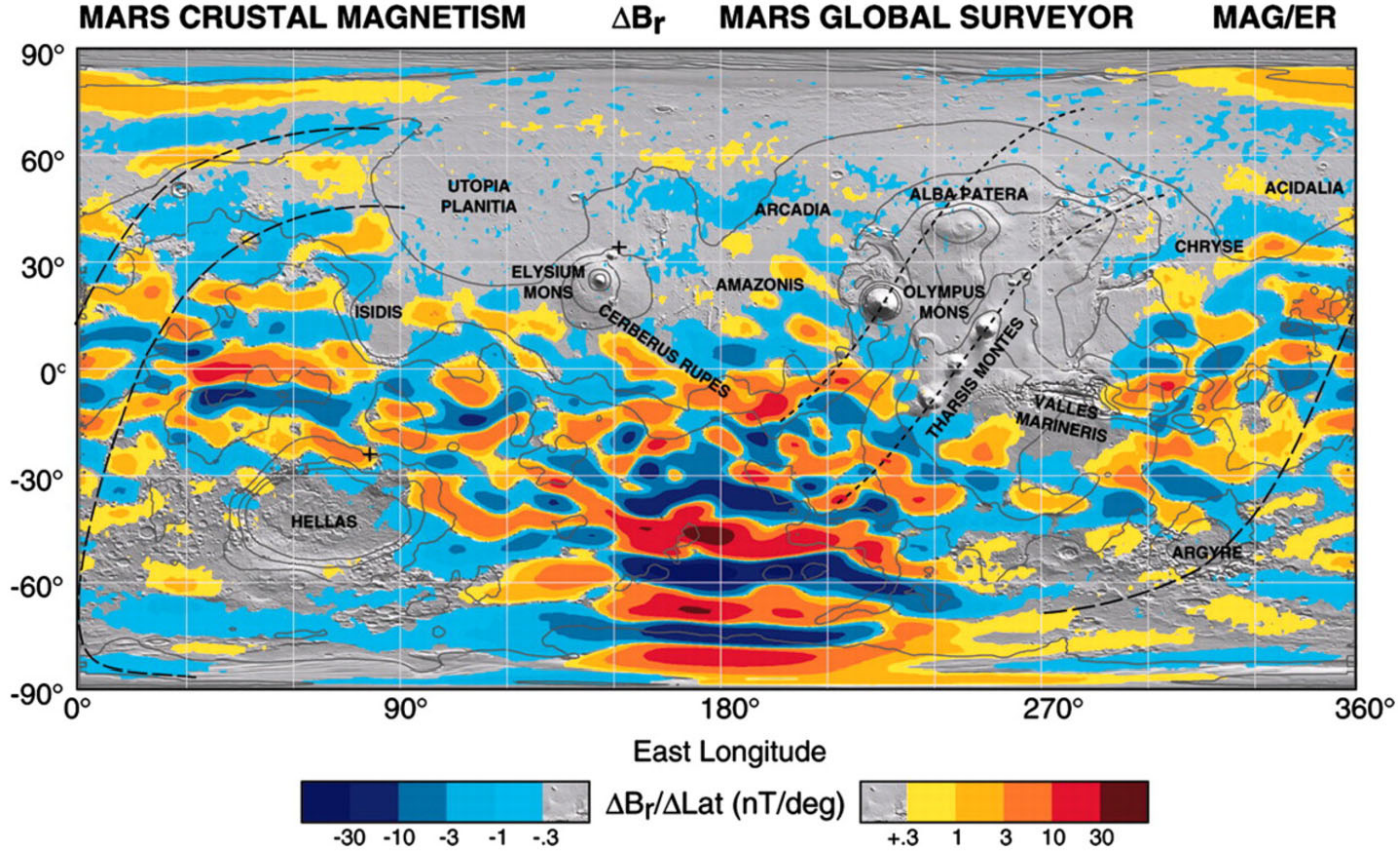
Map of the magnetic field of Mars observed by the MGS satellite at a nominal 400-km altitude. Each pixel is colored according to the median value of the filtered radial magnetic field component observed within the $1^{\circ }\times 1^{\circ}$ latitude/longitude range represented by the pixel. Reproduced from Fig. 1 of Connerney et al. (2005). Copyright 2005, The National Academy of Sciences

The discovery of the localized but intense crustal magnetic fields at Mars was one of the most important findings of the MGS mission (Acuña et al. 1998, 1999; Mitchell et al. 2001; Brain et al. 2003; Connerney et al. 2005). The distribution of these crustal field anomalies mimics the Mars topography to the first order, where most of the strongly magnetized crusts reside in the southern highlands and the weakly magnetized regions include giant basins, volcanic provinces, and the northern lowlands. This is demonstrated in Fig. 11, where $\Delta \mathrm{Br}/\Delta \mathrm{lat}$ is plotted in a longitude-latitude map, showing how the radial component of the crustal fields changes with latitude. The existence of crustal magnetism suggests that Mars once had a global magnetic field powered by a core dynamo but the characteristics of the past dynamo remain unknown and debated. Nonetheless, it is strongly tied to the planet’s evolution as well as the loss of its atmosphere. Crustal field models have been developed based on MGS measurements, such as Cain et al. (2003), Arkani-Hamed (2001), Langlais et al. (2004), Morschhauser et al. (2014), which also enable a deeper understanding of the Mars crustal fields and its interaction with the solar wind.

The MAVEN mission provided magnetic field vector measurements at altitudes below the MGS sampling limit of 400 km, down to 120–150 km altitude. This offers a valuable additional dataset for the modeling of the crustal fields at the surface. For example, Langlais et al. (2019) have improved their model resolution and small scales and well-defined features associated with geographical signatures show up in the new model. Additionally, surface magnetic field measurements by the InSight lander (Johnson et al. 2020) and the Tianwen-1 rover Zhurong (Du et al. 2023), provide further constraints on our understanding of the Martian crustal magnetism.

In terms of the solar-wind interaction, Mars’s crustal fields make this interaction very complicated. Before the MAVEN era, it was most commonly considered that Mars’s magnetosphere was predominantly induced-like, very much similar to Venus (e.g. Nagy et al. 2004; Bertucci et al. 2011), but the physical processes near strong crustal fields, such as magnetic reconnection (e.g. Halekas et al. 2009; Harada et al. 2018), auroral emission (e.g. Bertaux et al. 2005; Schneider et al. 2021) and associated auroral electron acceleration (e.g. Leblanc et al. 2006; Brain et al. 2006a; Halekas et al. 2008; Leblanc et al. 2008; Gérard et al. 2015; Soret et al. 2016; Shane et al. 2016; Xu et al. 2022c,b), could be similar to those at planets with intrinsic global dipole fields, such as Earth (e.g. Nagy et al. 2004). Enabled by MAVEN’s comprehensive plasma and field measurements, studies have shown that these localized crustal fields exert a global effect on the Mars magnetosphere, such as the tail twisting. Consequently, it is now widely accepted that Mars has a hybrid magnetosphere heavily influenced by these intrinsic crustal fields.

**Fig. 12:**
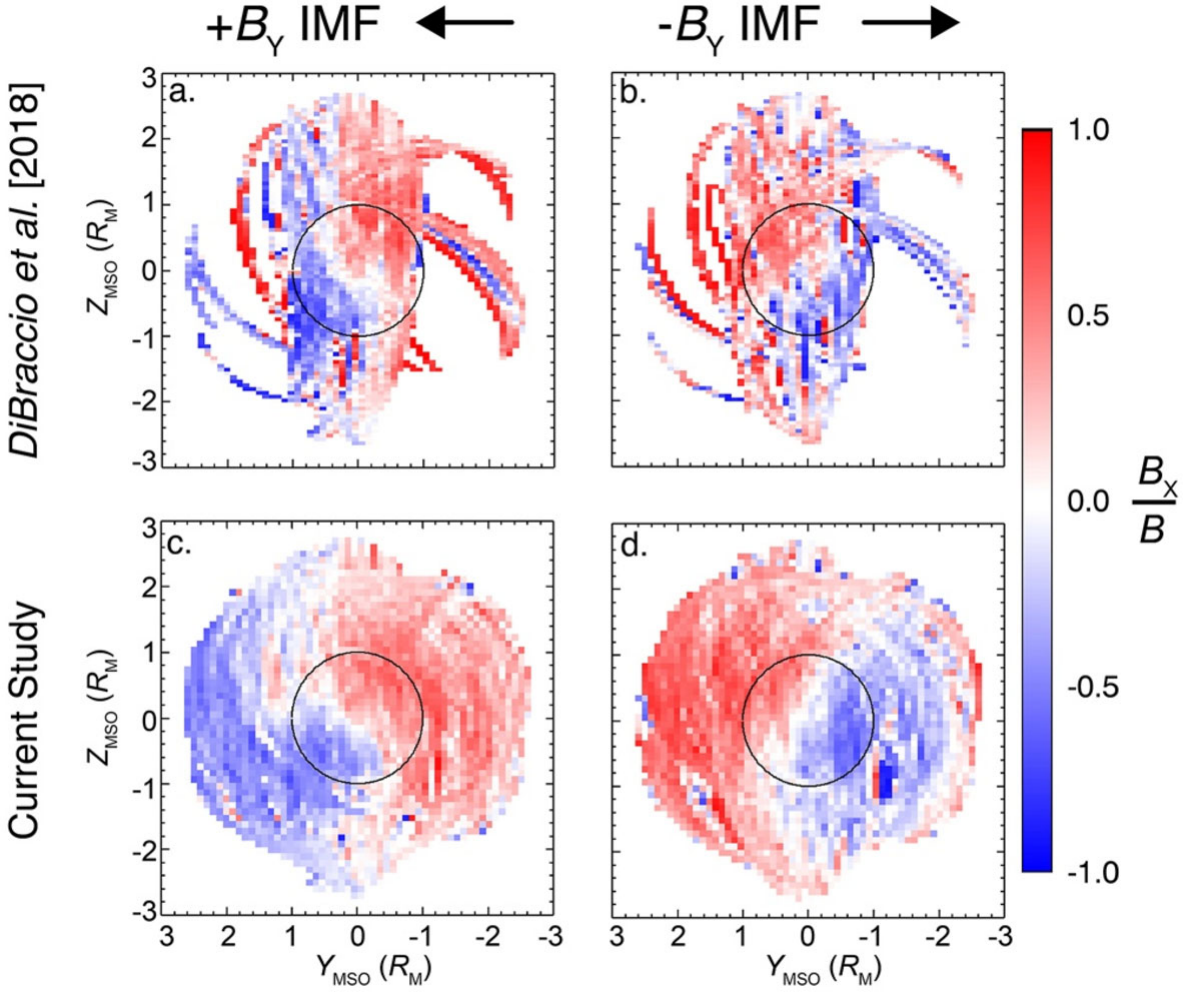
Magnetic maps of the normalized, average $B_{X}$ MSO component in the Martian magnetotail are shown for $+B_{Y}$ IMF (left column) and $-B_{Y}$ IMF (right column) cases. The maps are projections in the Y-Z MSO plane, viewed from the magnetotail toward Mars. The red bins ($+B_{X}/B$) correspond to the sunward tail lobes, while blue bins ($-B_{X}/B$) correspond to the antisunward tail lobes. Panels (a) and (b) show the results reported by DiBraccio et al. (2018), and panels (c) and (d) display updated maps by DiBraccio et al. (2022), associated with a larger statistical sampled (5940 selected orbits). Reproduced with permission from DiBraccio et al. (2022), copyright by the author(s)

Several recent studies reveal that the magnetotail of Mars displays features present in both induced and intrinsic magnetospheres, giving rise to a hybrid magnetosphere (e.g. Luhmann et al. 2015; DiBraccio et al. 2018, 2022; Curry et al. 2022; Dubinin et al. 2023; Xu et al. 2023b). A twist in the magnetotail current was found in the Martian magnetosphere using MAVEN observations (Dubinin et al. 2017; DiBraccio et al. 2018). The direction of the twist varies with the IMF dawn-dusk polarity, resembling the behavior observed in the terrestrial magnetosphere (e.g. Kaymaz et al. 1994; Russell et al. 1980a; Dubinin et al. 1980). Figure 12 displays average magnetic field maps of Bx/B as a function of Y and Z MSO coordinates. The magnetotail lobes twist counterclockwise (relative to the X MSO axis) for +By MSO IMF and clockwise for −By IMF, deviating from the nominal draping morphology expected in purely induced magnetospheres. This study also investigated some of the key factors affecting the observed magnetotail twist of Mars, i.e, crustal field location, Mars’s seasons, and downtail distance. In particular, it was found that Mars’s magnetotail can twist up to 60 degrees from the expected location based on IMF draping. Furthermore, the authors concluded that the degree of twisting in the Martian magnetotail varies with downtail distance and is greater for +By IMF orientation compared to −By IMF.

**Fig. 13:**
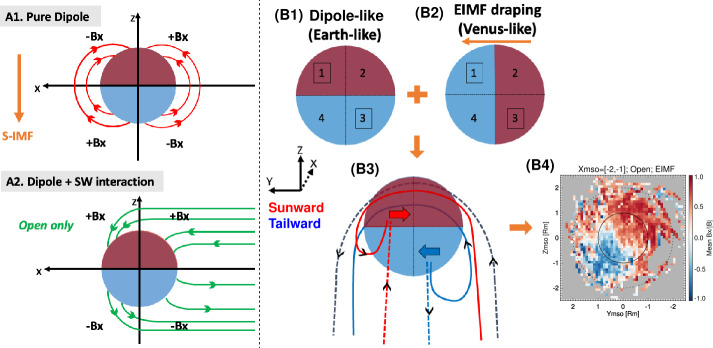
(A1-A2) Schematics show closed field lines from a southward-oriented dipole field (A1), which interacts with the IMF producing open field lines (A2). (B1-B4) Schematics show the tail field polarities (red for sunward and blue for tail-ward) from an intrinsic dipole-like field (B1) and the induced magnetotail comprised of draped IMFs (B2). (B3) Eastward IMF (dashed gray line) magnetically reconnects with open field lines (red and blue dashed lines) from quadrants 1 and 3, producing new open field lines (red and blue solid lines), overlaid over tail field polarities from the dipole-like field. (B4) The twisted tail configuration under the east IMF condition. Reproduced with permission from Xu et al. (2023b), copyright by the author(s)

Dubinin et al. (2023) revealed a dipole-like configuration of the model crustal field after averaging over many planetary rotations, which can magnetically reconnect with draped IMFs at locations of a larger shear between the two magnetic fields. The preferential reconnection sites give rise to a skewness in the geometry of the open magnetic fields that can explain a statistically steady twisting of the whole magnetosphere of Mars. In a similar effort, Xu et al. (2023b) used open and draped magnetic topology to isolate the intrinsic and induced components of Mars’s hybrid magnetosphere, separately. The induced component refers to the fact that the averaged magnetic fields for draped fields are mostly canceled when combining all (mostly east and west) upstream IMF conditions together. In contrast, the averaged magnetic fields for open fields over all upstream IMF conditions look dipole-like, with the northern lobe filled with sunward fields and the southern lobe filled with tailward fields. Xu et al. (2023b) suggested that the twisted tail configuration can be explained by the dipole-like lobes interacting with the typically induced tail lobes (mainly in the east-west configuration), as shown in Fig. 13. Overall, Mars’s twisted tail configuration reveals the hybrid nature of the Martian magnetosphere. These studies also suggest that the interaction between Mars and the solar wind is not just Venus-like, but also more Earth-like than previously thought. Up to date, there has been no report of a Venusian magnetotail twist, analogous to the one observed at Mars. Further studies are needed to better determine the structure of the Venusian magnetotail. There is an asymmetry found in the solar electric frame (He et al. 2021; Zhang et al. 2010) but is there a magnetotail twist in the Venusian magnetosphere, and if so, under which conditions it is clearly noticeable? Could crustal magnetic fields exist across the surface of Venus too, and should this be the case, what are their effects on the structure of the Venusian magnetotail?

### Magnetic Topology

One consequence of Mars’s crustal fields, interacting with the IMF is a dynamic and complicated magnetic topology, as a result of magnetic reconnection between the two fields. Magnetic topology is defined with respect to the Mars collisional atmosphere/ionosphere and classified into three types: closed with both footpoints of a field line embedded in the collisional atmosphere, open fields with one footpoint embedded in the collisional atmosphere and the other end connected back to the solar wind, and draped field lines not intersecting the atmosphere at all, but connected back to the solar wind on both ends. Magnetic topology is essential for understanding the Mars plasma environment as it guides the motions of electrons and low-energy ions (both are magnetized). For example, magnetic topology is important for characterizing energetic (order of eV to tens of keV) electron precipitation, causing heating (e.g. Fox and Dalgarno 1979; Sakai et al. 2016), ionization (e.g. Fillingim et al. 2007, 2010; Xu et al. 2016b; Cui et al. 2018; Adams et al. 2018), and auroral emission (e.g. Bertaux et al. 2005; Schneider et al. 2021; Lillis et al. 2022b). It is also important for characterizing the energization mechanisms of ions and low-energy ion escape (e.g. Ergun et al. 2015; Jakosky et al. 2018; Xu et al. 2018; Akbari et al. 2019; Collinson et al. 2019; Fang et al. 2008; Halekas et al. 2017; Cravens et al. 2017). Studies have also shown that the magnetotail and tail current sheet configuration is largely impacted by magnetic topology (e.g. Frahm et al. 2010; Luhmann et al. 2015; Xu et al. 2017b; Liemohn et al. 2017; Liemohn and Xu 2018; DiBraccio et al. 2018, 2022; Xu et al. 2020, 2023b).

**Fig. 14:**
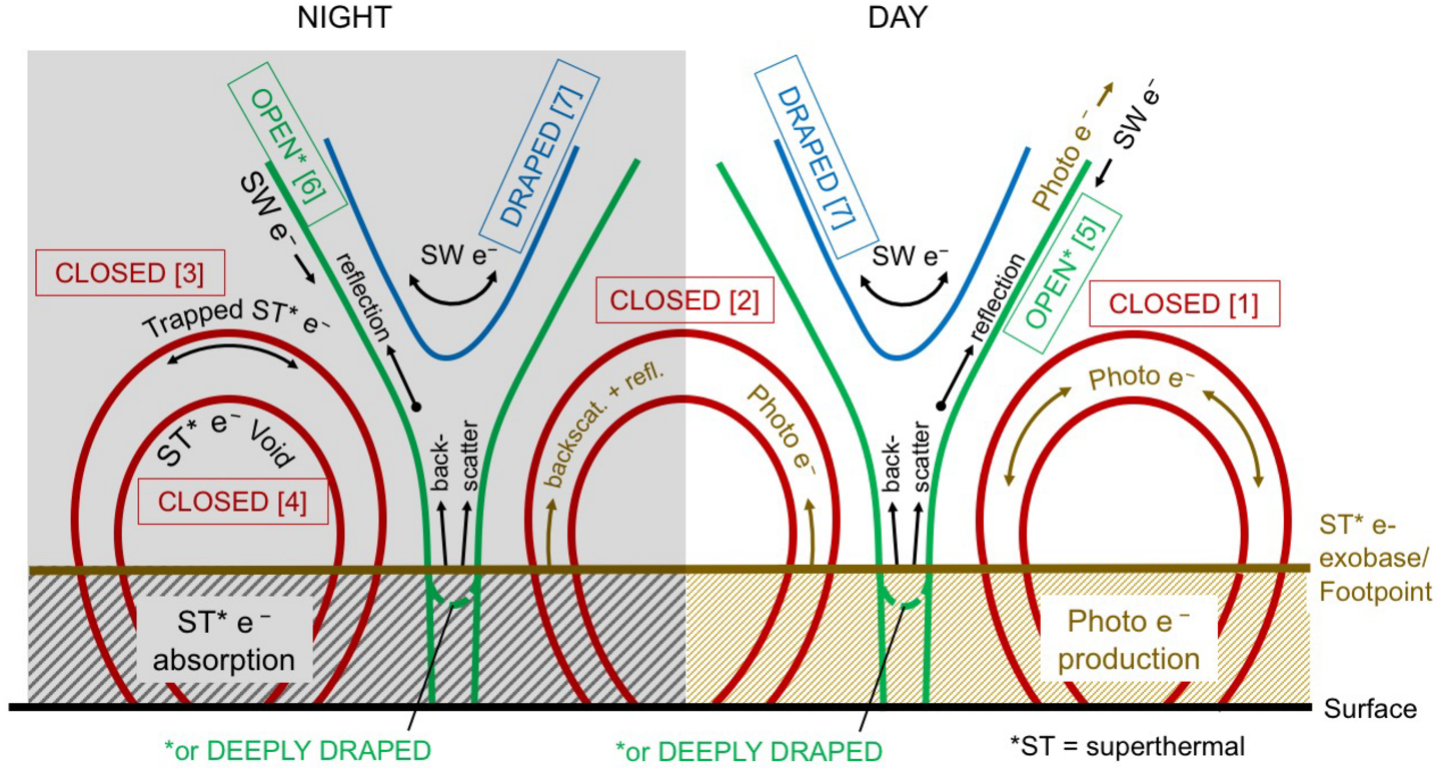
A schematic displaying the relationship of superthermal electrons and each magnetic topology at Mars. Red for closed, green for open or deeply draped (field line dips below the electron exobase but does not continue to the surface), blue for draped. Up to 7 topologies can be identified: 1: closed-to-day [C-D], 2: cross-terminator-closed [C-X], 3: closed-trapped [C-T], 4: voids [C-V], 5: open-to-day [O-D], 6: open-to-night [O-N], 7: draped [DP]. The left half, shaded in gray, is for the nightside and the right half for the dayside. Below the superthermal electron exobase, there is photoelectron production on the dayisde while the nightside atmosphere is an absorber for superthermal electrons. Photo e- is short for photoelectrons, SW e- for solar wind electrons. Reproduced from Fig. 2 of Xu et al. (2019). Copyright 2019 by AGU, reproduced with permission

Superthermal (∼1–1000 eV) electrons are good magnetic tracers. These electrons are generally magnetized and transport-dominated (over local production/loss processes) above the superthermal electron exobase (∼160 km altitude at Mars) and can travel thousands of kilometers within seconds, thus being used to infer the properties of plasma source/loss regions intersected by magnetic field lines at large distances from the spacecraft. The two main methods to infer magnetic topology include: (1) the presence of loss cones in the electron pitch angle distribution, i.e., very low electron fluxes near parallel and/or antiparallel directions, indicating atmospheric absorption and thus the intersection of the collisional atmosphere by a magnetic field line on one or both ends (e.g. Brain et al. 2007; Weber et al. 2017, 2019, 2020); (2) the presence of ionospheric photoelectrons (only produced in the dayside ionosphere as a product of photoionizing the atmosphere), identifiable by examining the electron energy distribution, in parallel and/or antiparallel directions, indicating the intersection of the dayside ionosphere by a magnetic field line on one or both ends (e.g. Xu et al. 2016b, 2017a, 2019). Xu et al. (2019) synthesized these two methods and provided the most accurate determination of magnetic topology with the MAVEN data. With the information of the footpoint(s) located in the dayside or nightside atmosphere, Xu et al. (2019) inferred up to 7 subtypes of magnetic topology at Mars, as illustrated in Fig. 14.

**Fig. 15:**
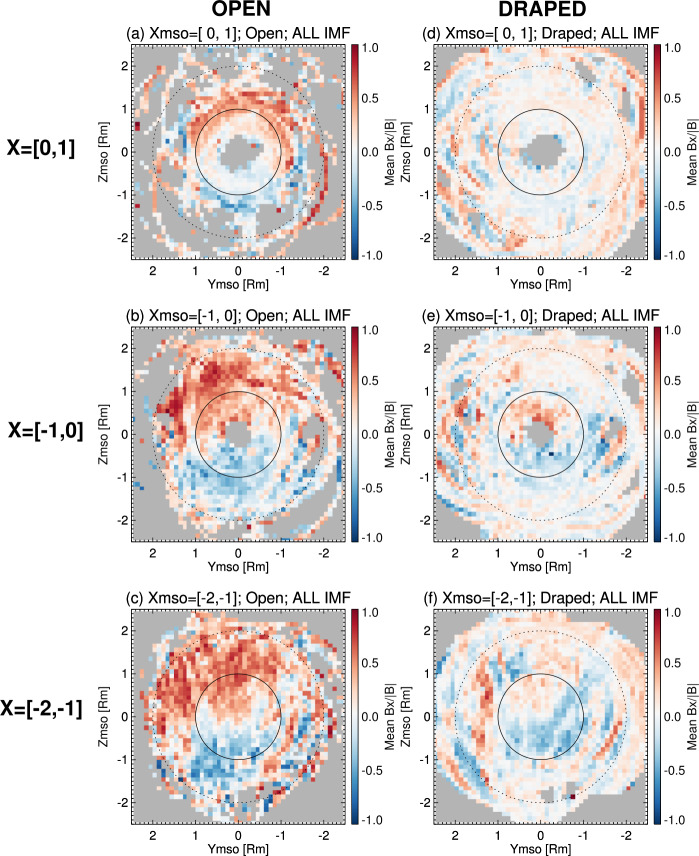
The averaged $B_{X}/|B|$ for open fields (a)-(c) and draped fields (d)-(f) in the MSO Y-Z plane for different $X_{\mathrm{MSO}}$ ranges, (a, d) $[0, 1]~\mathrm{R}_{M}$, (b, d) $[-1, 0]~\mathrm{R}_{M}$, and (c, f) $[-2, -1]~\mathrm{R}_{M}$, combining all IMF directions. Reproduced with permission from Xu et al. (2023b), copyright by the author(s)

Figure 15 displays the averaged tail polarity ($B_{x}/|B|$) over all upstream IMF conditions separated for open fields (a-c) and draped fields (d-f) at different tail distances (Xu et al. 2023b). It shows that the tail magnetic field is averaged to be near zero for draped fields when combining all IMF conditions, a feature of an induced magnetosphere. In contrast, the averaged tail polarity for open fields shows a north-sunward-south-tailward configuration resembling Earth’s magnetotail configuration, a persistent configuration throughout the tail region up to MAVEN’s apoapsis altitudes, revealing the intrinsic component of Mars’s magnetosphere. This example shows the importance of magnetic topology in understanding the nature of Mars’s complex magnetosphere.

**Fig. 16:**
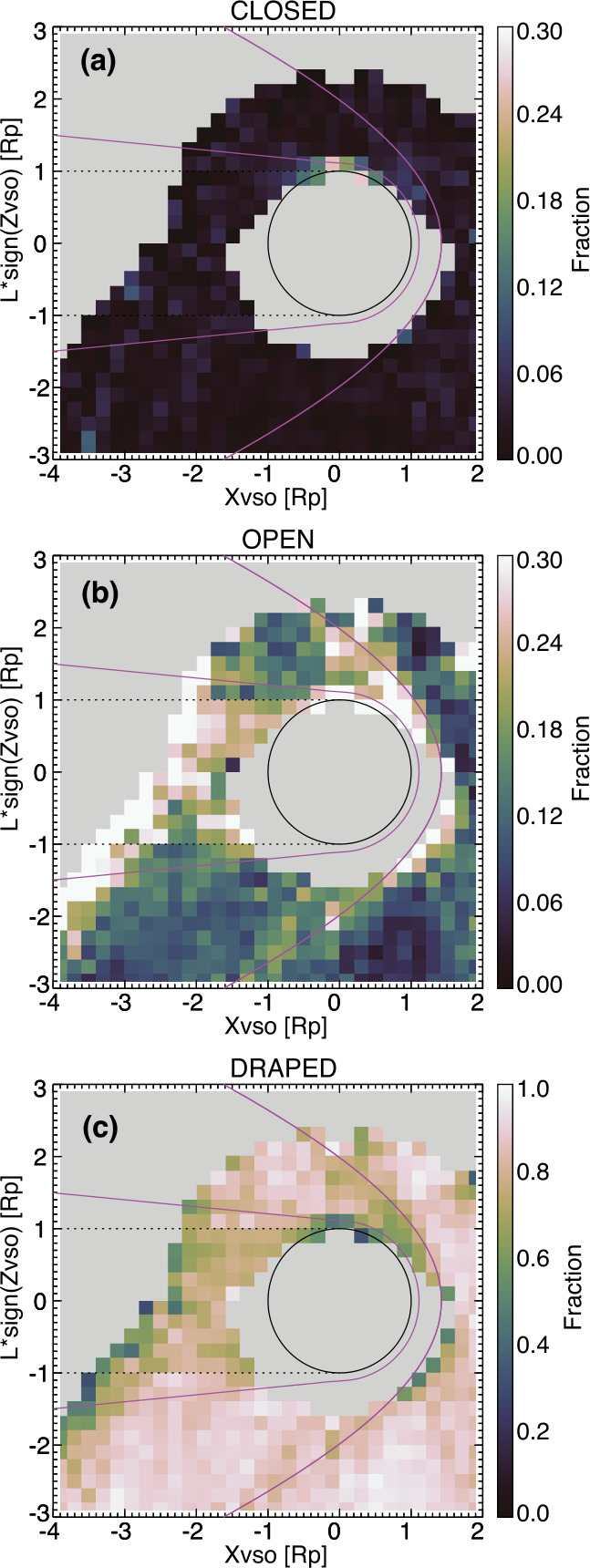
The occurrence rates of (a) closed, (b) open, and (c) draped magnetic field lines at Venus in the VSO cylindrical coordinates (with the cylindrical radial distance L signed by Zvso), inferred from the VEX electron and magnetic field observations. The magenta lines are the empirical bow shock and ion composition boundary from Martinecz et al. (2008). Note that the occurrence of the open topology near the bow shock in panel (b) is caused by misidentifications because of electron pitch angle anisotropy in the magnetosheath. The plot is inspired by the work of Xu et al. (2023a)

Despite Venus not having significant intrinsic magnetic fields (e.g. Phillips and Russell 1987; O’Rourke et al. 2019), the magnetic connectivity between the solar wind and Venus’s ionosphere is not as simple as expected. As magnetic topology at Mars is defined with respect to the collisional ionosphere rather than the surface, a similar definition of magnetic topology can be applied to Venus, which infers whether the IMF penetrates deep into its collisional atmosphere and differentiates whether there is a magnetic conduit between the Venusian ionosphere and the solar wind. Case examples in Xu et al. (2021) not only demonstrated the expected draped and open (most likely because of deep IMF penetration) topologies but also revealed the existence of cross-terminator closed field lines, a surprising magnetic topology at Venus that is not expected from a typical IMF draping geometry. Xu et al. (2023a) utilized a similar technique as Xu et al. (2019) and conducted a statistical analysis of the occurrence of various types of magnetic topology at Venus. This analysis revealed that closed and open topologies have high occurrence rates at low altitudes while the draped topology dominates other regions at Venus as shown in Fig. 16. It was also discussed in the same study that the open topology in the Venusian tail has an occurrence rate of 20%–30%, in contrast to 30%–50% at Mars. This suggests that IMF penetration alone could cause a significant fraction of the open topology in the Martian magnetotail but these two planets having different upstream drivers needs to be considered for a more rigorous comparison.

Xu et al. (2024), using the superthermal electron and magnetic field measurements from the 4th Venus flyby of the Parker Solar Probe spacecraft, revealed an even more rare occurrence, that is closed field lines in the magnetotail of Venus (at ${\sim} 2~V_{R}$) that are likely a result of magnetic reconnection of opposite-directed open field lines near the Venus magnetotail current sheet. This study also revealed a good correlation between the cold ion flow and open/closed topology for this tail flyby and ions on draped fields being more energetic. It suggests that, just like at Mars, the ion behavior in Venus’s magnetotail is well organized by magnetic topology, and that magnetic topology is one piece of key information for resolving ion escape mechanisms and thus the atmospheric evolution across various planetary environments.

The limitation of using superthermal electron observations to infer magnetic topology is that this technique is only informative of magnetic connectivity above the electron exobase. What is the true connectivity of open and closed field lines at Venus (and also Mars): simply a result of deeply penetrating IMFs or connected to the surface or interior? Furthermore, how does magnetic topology shape the ion escape at Mars and Venus, as some field lines have access to the main ionosphere and some do not, and which different ion acceleration forces are expected on different field lines?

### Flapping Motion of the Tails

A key feature highlighting the dynamic nature of magnetotails is the observation of multiple current sheet crossings during a single spacecraft orbit. This has been recently observed in the magnetotails of Venus and Mars, based on magnetic field and plasma data from Venus Express and MAVEN (e.g. Rong et al. 2015a; DiBraccio et al. 2017). These multiple crossings can be associated with various phenomena. As previously mentioned, changes in the IMF clock angle result in a rapid adaptation and rotation of the magnetotail lobes and current sheet. Additionally, changes in the external conditions, such as, the upstream solar wind dynamic pressure can result in steady flapping of the magnetotail current sheet. Furthermore, waves propagating along the current sheet cause a kink-like flapping, resulting in local magnetic field and plasma perturbations that the spacecraft can cross multiple times. Finally, in the case of Mars, open magnetic field lines can contribute to the formation of multiple current sheets in the Martian magnetotail (Luhmann et al. 2015).

**Fig. 17:**
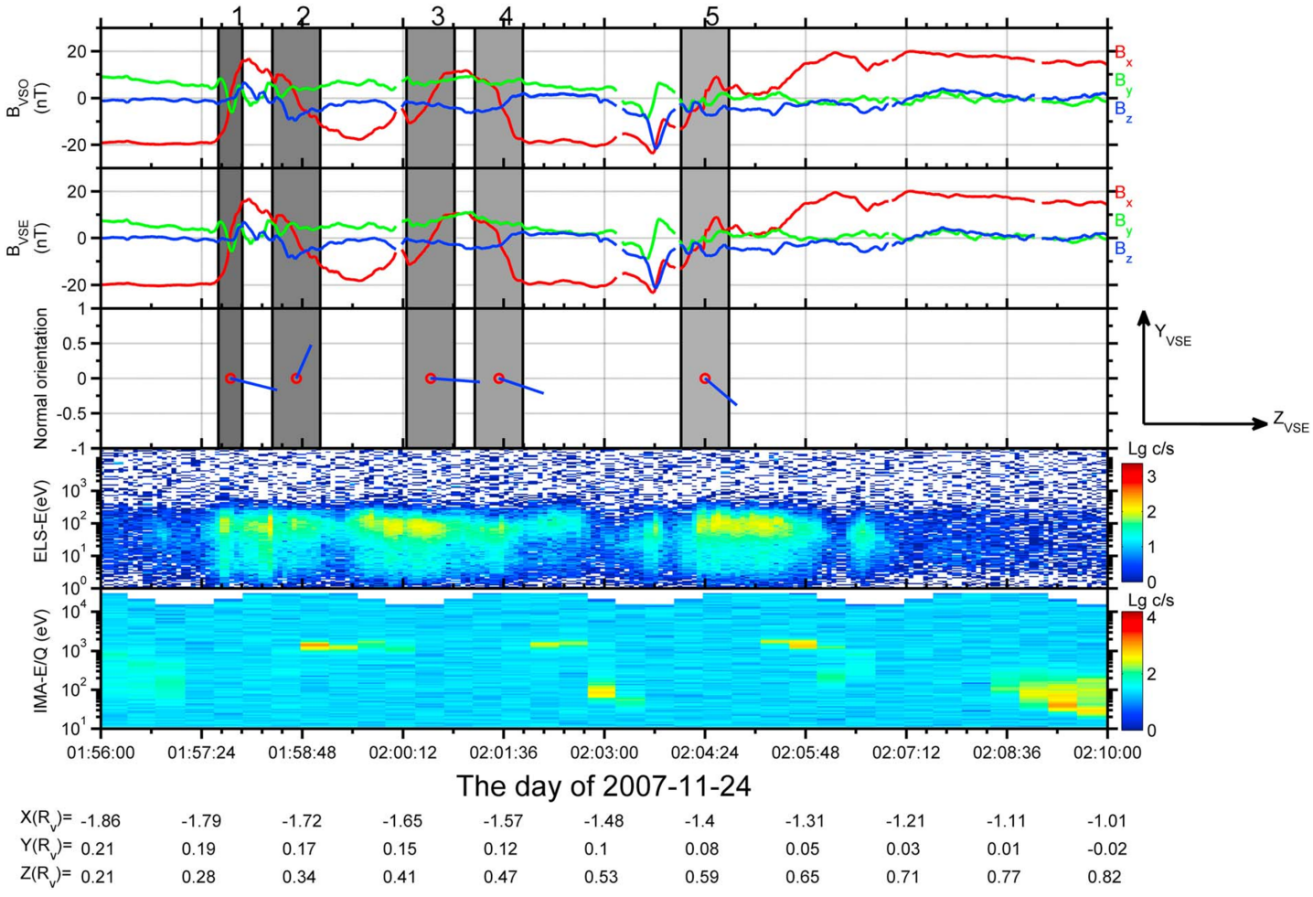
Venus Express magnetic field and plasma observations of tail current sheet flapping during 24 November 2007. The shaded grey areas correspond to intervals where MVA is performed to estimate the magnetotail current sheet normal. From top to bottom, magnetic field measurements in VSO and VSE (Venus-solar-electric) coordinates, the estimated orientation of the current sheet normal in the y-z VSE plane, and the energy spectrograms of electrons and ions, as a function of time. In the VSE coordinate system, the X-axis points to the Sun, the solar wind electric field aligns with the +Z direction, and the IMF lies in the XY plane with Y being positive. VEX position in VSE coordinates is shown at the bottom of the figure. Reproduced from Fig. 3 of Rong et al. (2015a). Copyright 2015 by AGU, reproduced with permission

The characterization of magnetotail flapping motion can be achieved using either single-spacecraft or multi-spacecraft observations (e.g. Rong et al. 2015a,b). Rong et al. (2015b) developed a technique using Minimum Variance Analysis (MVA) of magnetic field data from single-spacecraft observations (Sonnerup and Scheible 1998) to estimate magnetotail flapping properties and differentiate between various flapping motion modes, which was later applied to the Venusian magnetotail (Rong et al. 2015a). Figure 17 displays an example of multiple magnetotail current sheet crossings seen by Venus Express on November 2007. The average IMF was found to be nearly stationary based on measurements taken upstream of the Venusian bow shock along the inbound and outbound trajectory legs. Despite this, there is a notable correlation between increases in energetic ($\geq 100\text{ eV}$) electron flux (fourth panel) and changes in the Bx component (first panel), which are associated with multiple current sheet crossings during a single Venus Express orbit. MVA analysis of magnetometer (MAG) data, centered around each current sheet crossing, allows estimating its normal (third panel). The authors found that this normal can be significantly tilted in the plane perpendicular to the solar wind speed, as shown in the third panel. Additionally, the orientation of the normal can vary considerably from one crossing to another. Based on the statistical analysis of Venus Express MAG observations the authors concluded that the observed multiple current sheet crossings can be understood in terms of steady flapping and propagating kink-like flapping. In addition, the results suggest that sources of energy for the kink-like flapping mode are located near the magnetotail flanks.

**Fig. 18:**
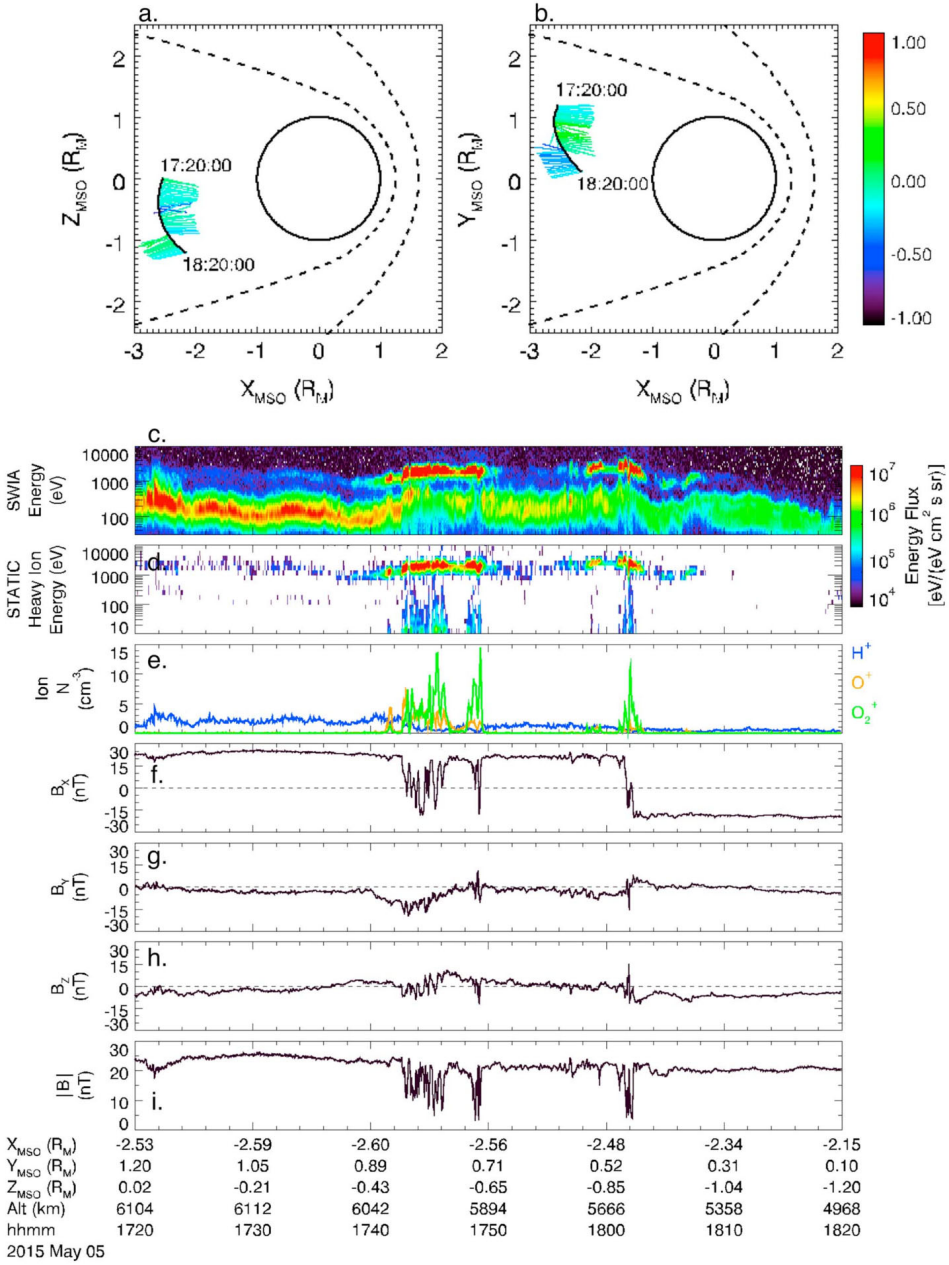
An interval of MAVEN’s orbit on 5 May 2015 is shown in (a) the meridional (X–Z MSO) plane and (b) the equatorial (X–Y MSO) plane. MAVEN plasma and magnetic field measurements: (c) SWIA ion energy; (d) STATIC energy of heavy ions with M/q ≥ 12; (e) densities of $\mathrm{H}^{+}$, $\mathrm{O}^{+}$, and $\mathrm{O}_{2}^{+}$; (f–i) magnetic field components, $B_{X}$, $B_{Y}$, and $B_{Z}$, in MSO coordinates and total field strength, |B|. Reproduced from Fig. 2 of DiBraccio et al. (2015). Copyright 2015 by AGU, reproduced with permission

The two types of flapping (kink-like and steady) are also observed in the Martian magnetotail. Specifically, DiBraccio et al. (2015) identified case studies where MAVEN detected several current sheet crossings during a single traversal of the tail, based on plasma and magnetic field data. Figure 18 displays an example of multiple current sheet crossings that occurred on May 5, 2015. In particular, it shows multiple changes in the polarity of the Bx component (panel f), which coincide with local minima in magnetic field strength (panel i) and planetary ions forming the current sheet (panels c-e), as detected by MAVEN MAG, Solar Wind Ion Analyzer (SWIA), and Suprathermal and Thermal Ion Composition (STATIC) instruments. These partial crossings suggest current sheet flapping. DiBraccio et al. (2017) applied the technique developed by Rong et al. (2015b) to conduct a statistical analysis of flapping in the Martian magnetotail. The authors found that steady flapping is more frequently observed than kink-like flapping. Similar to what was found in the Venusian magnetosphere, this study also suggests that the energy sources for plasma acceleration in the magnetotail current sheet are located near the tail flanks. However, unlike Venus’s magnetotail, most of the identified kink-like events are due to waves propagating in the direction opposite to the solar wind convection electric field, suggesting different energy sources. Additional studies investigating the energy sources driving magnetotail flapping at both Venus and Mars are needed.

### Waves in Induced Magnetotails

Within the collisionless environments of planetary magnetospheres, electromagnetic plasma waves and their interactions with charged particles (‘wave-particle interactions’) represent important mechanisms that facilitate energy and momentum transport through these systems. A plethora of work has studied the dayside electromagnetic plasma environment at Mars and Venus, in part because the most intense plasma wave activity is observed at and upstream of the bow shock, and within the magnetosheath (e.g. Scarf et al. 1980a; Russell et al. 1990; Dubinin and Fränz 2016; Brain et al. 2002; Strangeway 2004; Delva et al. 2011; Romanelli et al. 2024). Ruhunusiri et al. (2015) calculated transport ratios derived from MAVEN particle and field observations to show that Alfvén waves dominate in these regions, with little plasma wave activity observed in the magnetotail. MGS magnetometer observations analyzed by Espley et al. (2004) demonstrated that plasma waves, when present in the Martian magnetotail, are smaller in amplitude and typically linearly polarized, consisting of a mixture of wave modes. More recent analysis of magnetic and electric field power spectra by Fowler et al. (2017) demonstrated that lower frequency (<1 Hz) wave power is observed within the Martian magnetotail, albeit at wave powers 2–3 orders of magnitude smaller than in the dayside magnetosheath and bow shock regions. Higher frequency (>1 Hz) fluctuations were typically not observed in the magnetotail region.

A case study of MAVEN observations by Fowler et al. (2021) identified large amplitude ($\frac{dB}{B} \sim 10\mathrm{--}20\%$), compressional magnetosonic waves within the magnetotail and nightside ionosphere. These waves are generated on the dayside, upstream of the bow shock and are able to propagate across the draped magnetic field, around the planet and into the nightside. A comprehensive study to determine the occurrence rates of such events has not yet been carried out. Similar trends have been observed at Venus, with Alfvén waves dominating the wave mode within the dayside bow shock and magnetosheath, with significantly smaller wave occurrence rates observed on the nightside and in the magnetotail (Fränz et al. 2017; Dimmock et al. 2022). While large amplitude magnetosonic waves have been observed in the dayside magnetosphere at Venus (e.g. Fowler et al. 2024; Fränz et al. 2017), it is not yet clear whether analogous “nightside” magnetosonic waves exist. Similarly, large amplitude compressional waves have been observed in the dayside of Mars, where they energize the plasma likely via wave-particle interactions (Fowler et al. 2018; Collinson et al. 2018).

Various plasma waves are observed as part of the solar wind interaction with Mars and Venus. These waves have been shown to impact global scale processes. For example, recent global hybrid simulation results have demonstrated that ultra-low frequency (ULF) waves generated upstream of Venus and Mars modulate and enhance the oxygen ion escape rates, and the precipitation and planetary ion escape rates, respectively (Jarvinen et al. 2020, 2022). In contrast, even though different types of waves have been identified in the Venusian magnetotail (Hadid et al. 2021), relatively little study has been undertaken in the magnetotail regions. Wave generation processes, their impact on the magnetosphere including their potential role in the energization of precipitating particles, and any relevance to the energy budget of the nightside ionosphere are largely unconstrained.

### Current Systems

The capacity of the extended magnetotails of intrinsic magnetospheres to provide a storage of magnetic energy, persistent over a range of timescales, has long been known. In the most well studied example, the Earth’s magnetosphere, dayside reconnection and the subsequent transport and storage of magnetic flux in the magnetotail via the Dungey cycle is a major driver of the dynamics of the whole system. Recent reviews of the state of understanding of the terrestrial magnetotail can be found in Milan et al. (2017), Sergeev et al. (2012), Sharma et al. (2008). As Venus was initially more extensively studied with in-situ field and plasma measurements by PVO, much of the foundational work in understanding the current systems of induced magnetosphere was performed for that planet (Saunders and Russell 1986). While the basic structure of the induced magnetotails of Mars and Venus in the immediate vicinity of the planet can be said to be reasonably well understood, the role, if any, of induced magnetotails in mediating the rate at which energy flows through the induced magnetosphere and planetary system is only partially explored. This is due in part to the lack of available measurements in the extended or well-developed magnetotail regions at distances greater than a few planetary radii down tail, and indeed even to some extent in the near tail region.

The general structure of the Venus induced magnetosphere, extending into the tail, was elucidated from PVO measurements, including the principle dependence on the IMF orientation, and the presence of a tail current sheet (Saunders and Russell 1986). More recent work has suggested that other contributions to the magnetic environment may be persistent features of the induced magnetosphere, including apparently closed loops of field in the tail (Chai et al. 2016; He et al. 2021). At Mars, significant recent progress has been made using the extensive data sets available from Mars Express and MAVEN. In particular, MAVEN magnetometer data has been centrally used to explore the three-dimensional structure of the average magnetotail, exploiting the (near-) symmetry of the tail in the MSE (Mars-solar-electric) coordinate system. In the MSE coordinate system, the X-axis points to the Sun, the solar wind electric field aligns with the +Z direction, and the IMF lies in the XY plane with Y being positive. When combined with particle measurements, a comprehensive picture of the plasma flows and forces can be obtained (e.g. Halekas et al. 2017). Of primary consideration is the pile-up (“draping”) of magnetic flux into the dayside upper ionosphere, and the subsequent slowing of the passage of flux tubes through the Mars system compared to the ambient solar wind. The resulting tension developed in the field acts to accelerate mass-loaded ionospheric plasma toward the ambient solar wind velocity, through the $\vec{j}\times \vec{B}$ force (e.g. Dubinin et al. 1993, 2018; Lundin 2011), in the same overall structure initially exposed at Venus by Saunders and Russell (1986).

**Fig. 19:**
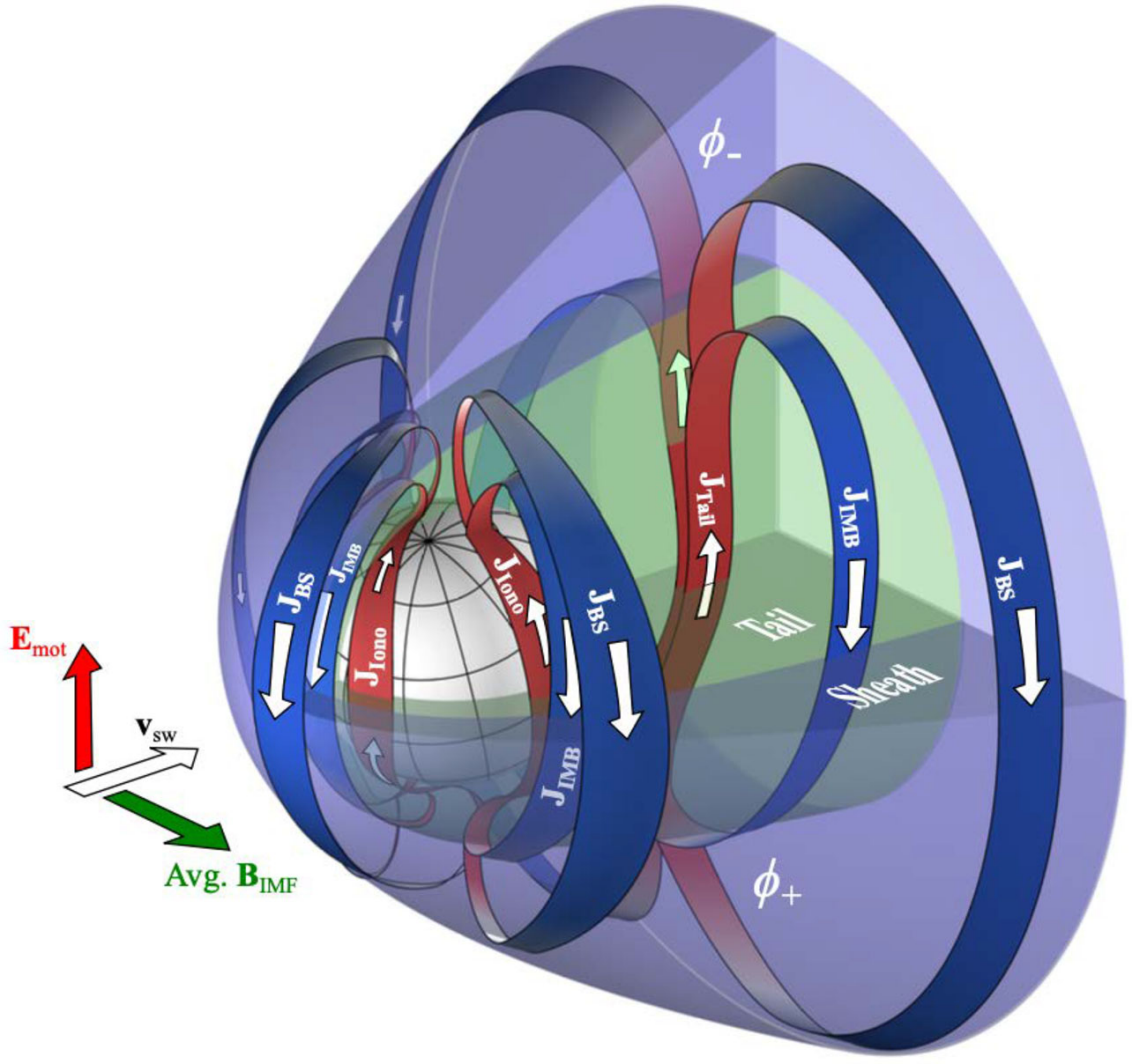
Illustration of the structure of induced currents in the Mars near space environment in the MSE frame. Currents are shown red or blue depending on the sense of energy conversion associated with them. Reproduced with permission from Ramstad et al. (2020), copyright by the author(s), under exclusive licence to Springer Nature

By determining the average magnetic field configuration around Mars in the MSE frame, Ramstad et al. (2020) have shown how the induced magnetotail can be understood as being due to analogues of the terrestrial magnetotail and magnetopause currents, the so-called “theta” current system, in which twin current loops (here fixed in MSE frame) generate the sunward and anti-sunward lobe magnetic fields. As shown in Fig. 19, in which the complete system is depicted, the tail current system, of which the tail current sheet (“$\mathrm{J}_{\mathrm{Tail}}$”) forms part, can in some sense be seen to be consistent with a projection down-tail of the ionospheric current system at the terminator. In Fig. 19, the bands of current depicted are shown blue where they are MHD “generators”, and red where they are “loads”, i.e. where they contribute to the conversion of kinetic into magnetic energy, and vice-versa. Plasma is energized and heated in the ionosphere and tail current sheets, while the magnetic field is deformed and/or enhanced from the ambient upstream values at the bow shock and IMB (Zhang et al. 2025). Smaller-scale but significant departures from a simplified, symmetric draping of the magnetic fields around Mars, have also been reported (Azari et al. 2023; Zhang et al. 2022) in recent analysis of MAVEN data, with the supposition that such asymmetries can be in part due to significant (anti-)sunward IMF component present in the nominal Parker-spiral direction. Additional twisting or deformation of the induced tail and its current systems from the most simple, symmetric structure that may be expected is likely present at Mars due to the highly asymmetric nature of the mass-loading and formation of the polar ion plume (e.g. Dong et al. 2015c; Ramstad et al. 2020). Simulation results are largely in line with observations in these respects (e.g. Wang et al. 2023b, 2024b; Modolo et al. 2016). Of particular note is the presence of additional current loops coupling the interior tail current sheet to the bow shock (Ramstad et al. 2020). The coupling of bow shock currents into the magnetotail current sheet (and indeed also dayside ionosphere) provides a further direct pathway for the exchange of energy from the solar wind to induced magnetospheres. Both measurements and modelling suggest that these currents can represent a significant source of the system energy budget (Wang et al. 2023b). Current systems in the ionosphere, on the other hand, seem to be driven by both the IMF from above and the neutral winds from below as shown by Gao et al. (2024). However, a more complete understanding of the ionospheric and atmospheric effects of these current systems remains to be developed, and will require examination of the lower reaches of these current systems (heating rates and the consequences of energy deposition being highly sensitive to the altitudes where these currents close, as discussed e.g. by Opgenoorth et al. 2010). The recent discovery of “sinuous” auroral forms in the Martian nightside by Lillis et al. (2024) is also tentatively connected to these tail magnetospheric current systems, although their apparently transient nature and variable alignment with respect to the upstream IMF remains to be understood in such case.

The $\vec{j}\times \vec{B}$ forces that are present in the draped Martian magnetotail can be studied in both simulations and in-situ measurements, via measurements of the magnetic field structure, and by assessing its direct effects — acceleration of (primarily) planetary ions toward the solar wind velocity vector (e.g. Halekas et al. 2017). On smaller spatial and temporal scales, more energetic plasma acceleration through reconnection in the tail current sheet has been reported (e.g. Eastwood et al. 2012; Harada et al. 2015, 2017). However, studies also suggest that smaller scale features within the induced magnetotail can often instead be attributed to ionospheric flux ropes, convected to their observed location with the ambient flow (e.g. Hara et al. 2017, 2022).

In the case of Venus, Titan and other bodies lacking significant intrinsic magnetic fields, much of what has been recently discovered at Mars is either already directly evidenced both in measurements and simulations, or is at least generally expected to also hold true given the nature of the other induced magnetospheres of the solar system. For example, while the spatial coverage afforded of the Venus system is not as complete as at Mars, the general pattern of the draped IMF field lines is well known, along with the field stresses in the magnetotail, all of which can only be understood as the result of coupled ionosphere-magnetosphere (and likely bow-shock) current systems commensurate with those seen at Mars. Likewise Titan, in the sub-magnetosonic regime.

Despite this progress, several open questions remain:

- Ionospheric currents coupling the induced magnetosphere to the ionosphere and upper atmosphere are expected to deposit energy in through Joule-heating. Is this a significant part of the thermospheric energy budget in the relevant locations?
- How do the strength of these currents vary with driving solar wind conditions, seasonal variations in ionospheric conductivity, etc.?
- Do Mars’s crustal fields modify these currents on either global or regional scales?
- Do these current systems have any causal relationship to sinuous aurora at Mars?
- Are there any fundamental differences in the sub-sonic regime at Titan when compared to Venus and Mars?
- How far down tail are the current systems and field stresses involved still able to contribute to the bulk acceleration of ionospheric plasma?
- Very little analysis has been possible thus far with accurate, contemporaneous measurements of the solar wind and IMF — are any of the observed features of these systems attributable instead to external factors, convolved with the generally biased spatial and seasonal coverage?

## Ion Escape and Plasma Flows: Mars vs Venus

### Ion Escape and Related Processes

The atmospheres of Mars and Venus are subject to a number of erosion processes; in this paper, we focus on ion escape as other escape processes, such as photochemical escape and Jeans escape, do not substantially impact the magnetotail structure and dynamics at Mars or Venus (Lammer et al. 2008; Gillmann et al. 2022; Jakosky et al. 2018).

**Fig. 20:**
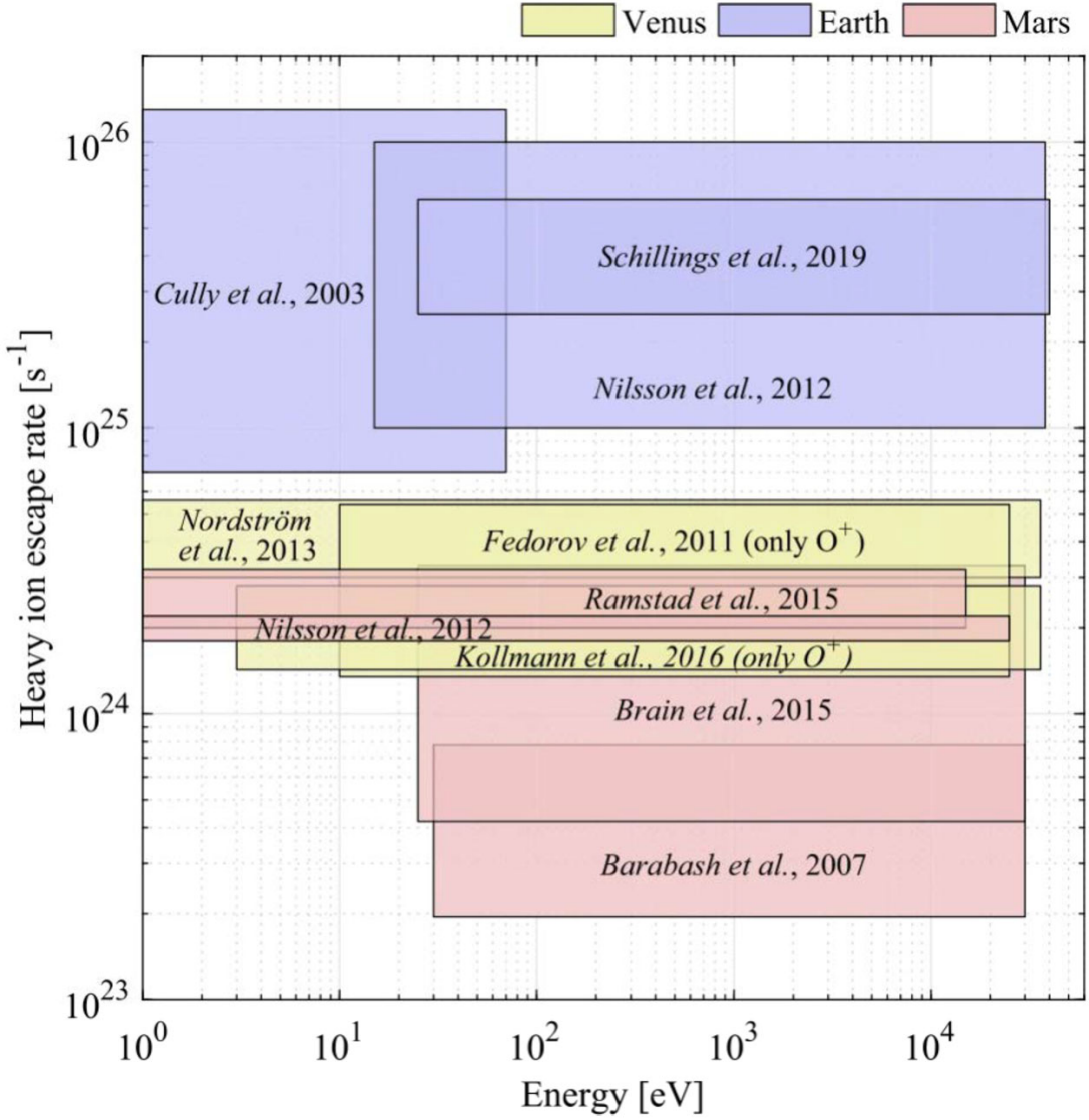
Observed $\mathrm{O}^{+}$ and heavier ion escape rates from Mars (red), Venus (yellow), and Earth (blue). The observations of these studies come from various altitudes, regions, and phases of the solar cycle. Reproduced with permission from Ramstad and Barabash (2021), copright by the author(s), under exclusive licence to Springer Nature

Ion escape has been studied by a number of missions at Mars and Venus, as described in Sect. 2. At Mars, Phobos 2, MEX, MAVEN and a small number of flybys have flown with instrumentation suites that have been able to observe escaping planetary ions (see Tables 1 and 2). At Venus, PVO, VEX and recent flybys by Parker Solar Probe, Solar Orbiter and BepiColombo have observed escaping planetary ions in the magnetotail. Ion escape rates from both planets span a considerable range due to variations in the environment caused by e.g., the solar cycle, seasons, crustal fields location, instrument limitations, orbital coverage, which have been previously summarized in Futaana et al. (2017), Ramstad and Barabash (2021), Dong et al. (2017), Jakosky et al. (2018), and are further elaborated upon in Sect. 5.3. Figure 20 provides a quantitative perspective on observed escape rates for the missions in orbit at Mars (red) or Venus (yellow), together with a comparison to the escape from the magnetized Earth (blue), which interestingly is significantly higher than that of both unmagnetised planets.

The ion loss at Venus and Mars is considered to be strongly affected by the difference in planetary mass, i.e. escape energy, leading to Mars being considered as having a source-limited escape, while Venus has an energy-limited escape (Ramstad and Barabash 2021). One argument for this could be the almost order of magnitude difference in $\mathrm{O}^{+}$ escape rates (i.e. ${\sim} 10^{24}$ $\mathrm{O}^{+}/\mathrm{s}$ at Mars and $10^{24}$–$10^{25}$ $\mathrm{O}^{+}$/s at Venus). However, it should be noted that neither VEX nor PVO could discern the mass of different atmospheric constituents and thus the derived loss rates are for “heavy ions” (i.e., $\mathrm{O}^{+}$, $\mathrm{O}_{2}^{+}$ and $\mathrm{CO}_{2}^{+}$) vs. “light” species (Fedorov et al. 2011; Persson et al. 2018; Nordström et al. 2013; Brace et al. 1987).

**Fig. 21:**
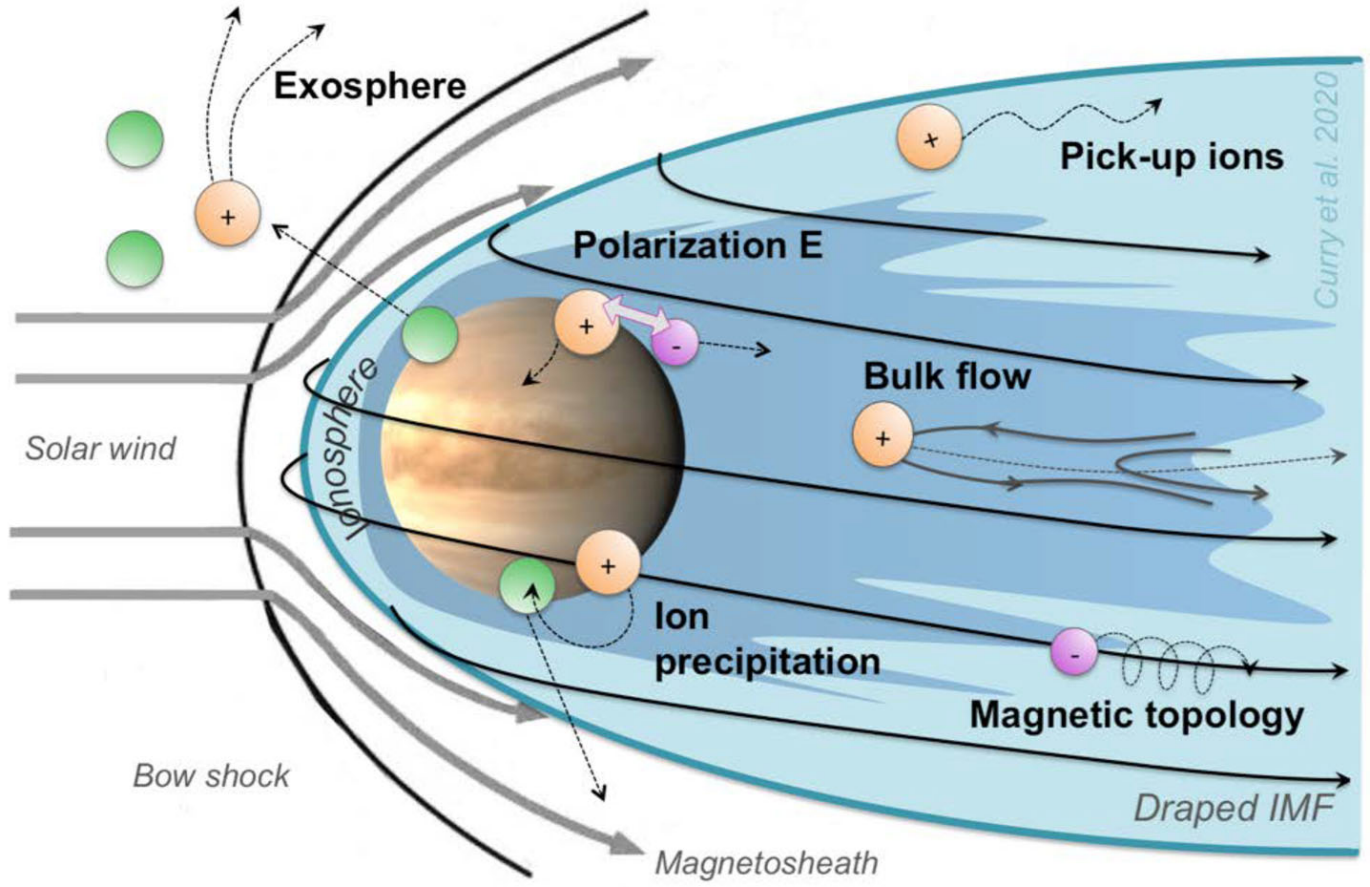
The physical processes responsible for ion escape within induced magnetotails

The physical processes responsible for ion escape are highly relevant to how mass and energy are transferred within induced magnetotails, as illustrated in Fig. 21. At Mars, cold ion escape has been observed by combined ion and radar observations from MEX (Fränz et al. 2015). MAVEN observations have provided super-cold low energy observations of escaping ions (≤5 eV). Given that the escape energy for $\mathrm{O}^{+}$ at Mars is only about 2 eV, these observations highlight the importance of magnetic topology and of the pressure gradient term in Ohm’s law (Curry et al. 2022). On the other hand, VEX and PVO were not able to measure planetary ions below about 25 eV and thus how cold ions escape at Venus, which has a much larger gravitational well, remains an open question. Similarly, at the other end of the energy spectrum, the polar plume was first observed at Mars with MAVEN, which presents a strong escape channel for ions at Mars (Dong et al. 2015c). VEX also observed a similar plume at Venus, but due to the deeper gravitational well and the higher IMF strength at Venus the fan has a significantly more compact shape than at Mars (Curry et al. 2014; McEnulty 2010). Nevertheless, the ion pickup of the Venusian ion plume in the magnetosheath of Venus was shown to contribute with around 30% of the total ion escape at Venus for the “heavier” ion species (Masunaga et al. 2019).

Observations of ion fluxes in the magnetotails of Venus and Mars have indicated that processes thought to only occur at magnetized planets are present also in induced magnetospheres, such as magnetic reconnection. At Mars, magnetic reconnection events indicate a strong link to the crustal magnetic field locations (Harada et al. 2018; Cravens et al. 2020; Lin et al. 2024). However, at Venus there is not yet any evidence for crustal magnetic fields, although evidence for magnetic reconnection occurring in the magnetotail has been presented (Zhang et al. 2012; Gao et al. 2021). In addition, at Venus there are large fluxes of both “heavy” and “light” ion species flowing back toward the planet in the magnetotail, the so called “return flows”, which decrease the total escape rates at Venus, in particular during low solar EUV radiation flux periods (Kollmann et al. 2016; Persson et al. 2018, 2020; Masunaga et al. 2019). The presence of return flows that affect the total escape at Mars has not yet been observed, indicating that the difference in planetary mass may play a part. Additionally, simulations suggest that sunward oxygen ion fluxes at Mars are small and do not significantly affect the total ion loss (Dubinin et al. 2024). Other escape structures present in the magnetotails of Venus and Mars are further elaborated in Sect. 5.4.

**Fig. 22:**
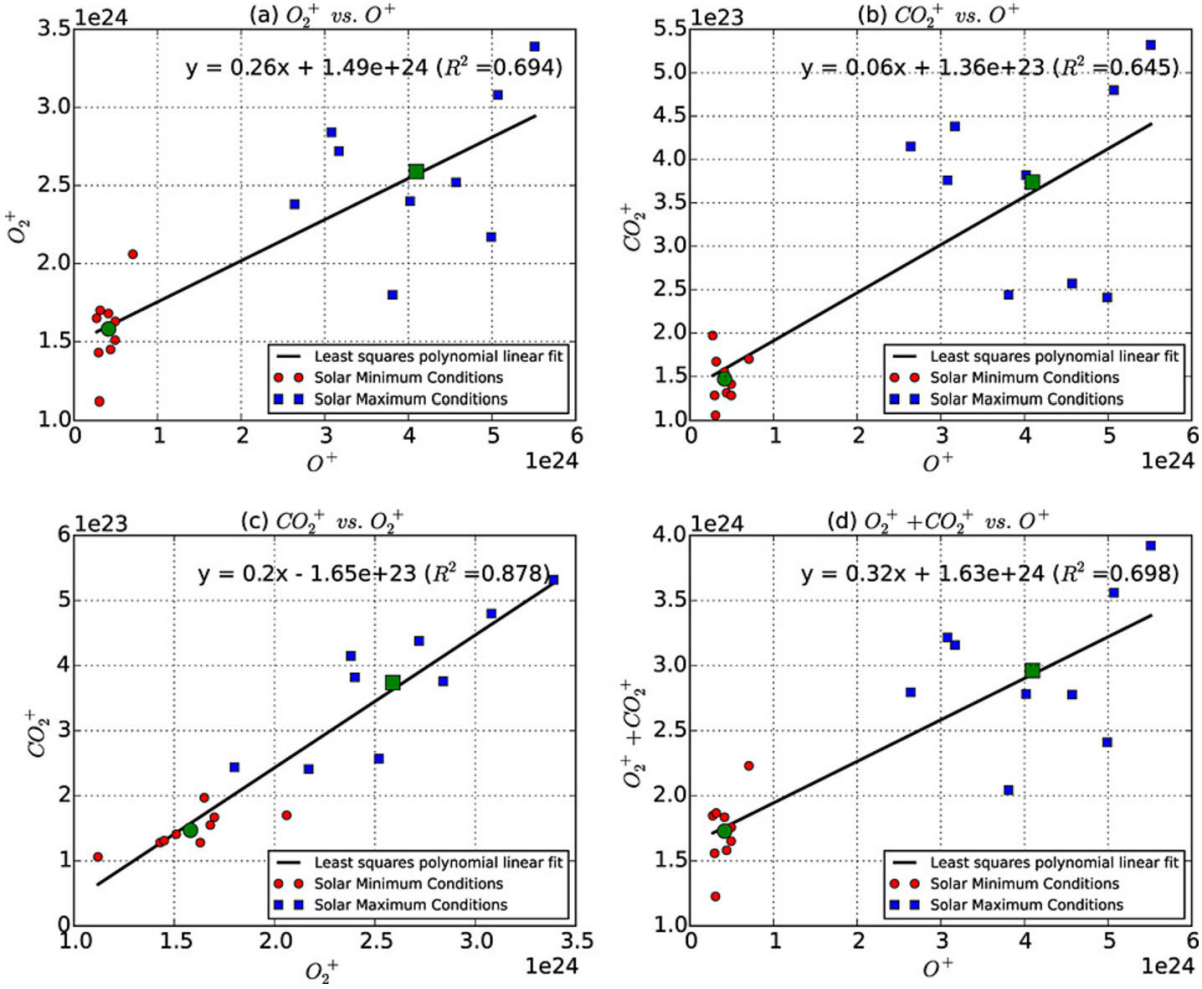
Least squares fit of the simulation results of ion escape rates at Mars for solar minimum and solar maximum conditions, shown as red circles and blue squares, respectively. The corresponding mean values are depicted in green. Reproduced from Fig. 6 of Dong et al. (2015a). Copyright 2015 by AGU, reproduced with permission

In addition to observations, both MHD and hybrid models have served as important tools for predicting dynamics such as Mars’s twisted tail and polar plumes as well as ion escape rates (Modolo et al. 2005; Ma et al. 2014; Curry et al. 2014; Dong et al. 2015a; Modolo et al. 2016). In particular, when observations are either lacking or the sample size is too small, models that capture the solar wind interaction at Mars and Venus are critical until additional assets can make observations with sufficient resolution. Dong et al. (2015a) was able to simulate a number of permutations of solar EUV, solar wind, seasonal and solar cycle to provide a more quantitative approach to estimate ion escape for different ion species. In Fig. 22 the escape rates of various ion species during solar minimum and maximum conditions are shown (Dong et al. 2015a). Ledvina et al. (2008) provided a much longer discussion on applicable models to weakly magnetized bodies as well as their limitations. While models can continue to provide excellent global perspectives of both the Martian and Venusian systems and their ion loss, observations of extreme conditions -to broaden the parameter space- will be necessary to validate the physics of these models.

### Comparative Scaling and Energetics

A series of studies have estimated the systematic ion escape response to variations in upstream solar wind and EUV conditions at Mars and Venus. Over time, these studies collectively revealed opposite scaling trends at the two planets, despite similar typical rates. Specifically, intense solar wind increases the tailward ion escape rate from Venus (Persson et al. 2020; Masunaga et al. 2019), yet escape through Mars’s induced magnetotail is largely insensitive to solar wind variations (Dubinin et al. 2017; Ramstad et al. 2017b, 2018). Conversely, intense solar EUV reduces the ion escape rate from Venus (Kollmann et al. 2016; Masunaga et al. 2019), while enhancing the Martian ion escape rate (Ramstad et al. 2018; Dong et al. 2023).

The reason for this apparent interplanetary incongruence can be understood by analyzing the dynamics and energetics of the ion flows. At Venus, the reduction in the escape rate with EUV arises from increasingly larger fractions of outflowing ions returning to the atmosphere, i.e. as return flows (Kollmann et al. 2016). At Mars, on the other hand, increasing EUV reduces the energy of the outflowing ions, yet escape rates increase (Ramstad et al. 2017b). Martian ion energetics in response to solar wind conditons also show that the energy flux associated with intense solar wind conditions accelerates already escaping ions to higher energies, though yields no strong effects on the ion escape rate. It is likely that return flows play some role in the escape process at Mars (Zhang et al. 2022), although a truly comparable study is yet to be completed. As noted by Ramstad and Barabash (2021), these qualitative differences indicate that the escape process is limited by intrinsically different factors when operating at each planet, respectively.

### The Impact of Space Weather on Ion Escape

The induced magnetospheres of Mars and Venus are created through the interaction between the solar wind and the planet, and it is therefore natural that space weather variations influence the properties of these magnetospheres, also in terms of atmospheric escape. As the escape to a large extent occurs through the tail (Barabash et al. 2007a), the tail will be affected by the space weather conditions. Changing upstream conditions mean a change to the energy and momentum provided to the system. Additionally, since the induced magnetosphere itself reacts and reforms due the upstream conditions, the efficiency of the transfer of energy and momentum from the solar wind to Mars/Venus will change. For instance, the size of the bow shock and magnetic pileup boundary expands and contracts with upstream conditions (Edberg et al. 2008, 2009a; Signoles et al. 2023), and therefore the effective area of the interaction between the solar wind and the planet will also change. The strength of the piled up magnetic field around the planet will vary and so will the convective electric field, leading to changing conditions for any potential escape down through the tail.

Space weather could here conceptually mean any variation to the solar wind and IMF properties. These variations can either be smaller changes such as day-to-day fluctuations, or more significant changes such as impacts of corotating interaction regions (CIRs) or coronal mass ejections (CMEs). For the latter, a number of studies have been carried out in the past decades, with somewhat mixed results as will be described below.

Using one year of MEX measurements, Lundin et al. (2008) found that the escape rate of Mars’s ionosphere increases with increased solar wind forcing (dynamic pressure). Nilsson et al. (2010, 2011, 2012) subsequently expanded on that study and found a positive correlation between the escape flux and the solar wind flux (as well as with solar EUV flux). However, using the same instrument as these MEX-based studies, but with an expanded data set, Ramstad et al. (2015, 2017a) reached an opposite conclusion: the escape flux decreased with increasing solar wind flux. For the case of Venus, Persson et al. (2020) used Venus Express measurements to show that the escape rate of ions from Venus increased with increased solar wind flux, but mainly so for solar minimum conditions when the EUV flux is low. The ratio between escaping oxygen and hydrogen ions also change from solar maximum to solar minimum conditions (Persson et al. 2018). However, later simulations did not reveal any significant increase in escape flux when the dynamic pressure increases (Katrougkalou et al. 2024). They instead suggested that the ion temperature was more important than the dynamic pressure.

In addition to these statistical studies there have been a number of case studies on space weather events and their impact on Mars and Venus escape rates. Dubinin et al. (2009) observed a CIR impact on Mars using MEX data, which significantly increased the ASPERA-3 observed flux, by a factor of 10. Futaana et al. (2008) observed significantly increased escape fluxes of planetary ions from both Mars and Venus when a burst of solar energetic particles impacted both planets. Luhmann et al. (2007) and Luhmann et al. (2008) studied a few CMEs at Venus and presented VEX measurements indicating that the planetary ion escape flux could be increased by a factor of at least 10 during some events. However, Ramstad et al. (2017b) studied another CME impact on Mars and found no increase in escape rate but rather an increase in the energy of the escaping particles by a factor of 10–20. Edberg et al. (2010b) and Edberg et al. (2011b) performed statistical studies of CIR and CME impacts on Mars and Venus, and found that the escape increased by a factor of 2.5 and 1.9 on average, respectively, during these events. Jakosky et al. (2015a), using MAVEN observations, reported increased ion escape rates, up to a factor of 10, caused by an ICME event that arrived at Mars in March 2015. Mayyasi et al. (2018) and Lee et al. (2018) estimated an enhancement of hydrogen and ion escape rates (to a factor of 5 and ∼2, respectively), caused by a particular intense ICME event, which occurred in September 2017 and was observed arriving at Mars with MAVEN, once again demonstrating that the planet’s upper atmosphere and ionosphere are significantly affected during such events, leading to increased escape rates. Most event studies hence seem to suggest a positive correlation between escape flux and space weather in terms of increased dynamic pressure or solar wind flux, with most simulations thereof also supporting this (e.g. Dong et al. 2018; Zhang et al. 2023; Li et al. 2023).

However, from single events and single-spacecraft measurements it is challenging to fully understand the response of Mars and Venus to space weather events. It may very well be that depending on the properties of the individual event the system responds differently. Dedicated two-spacecraft measurements are needed to more accurately answer outstanding questions on how the escape through the tail varies with upstream drivers. During space weather events, several factors might change simultaneously such that it is challenging to determine which factor is actually important for changing the escape rate. Outstanding questions remain on the relative contribution of different escape mechanisms, and how the escape rates change for different ion species. Which processes are increased and which are suppressed during space weather events? There is, for instance, a lack of measurements and studies regarding the importance of wave–particle interactions, which might also vary with changes in the solar wind. Ramstad et al. (2015) pointed out that in the case of CIR and CME impacts it may be that the system responds differently to the compression region and the rarefaction region of each event, such that timing is crucial when studying these impacts. Measurements at one location (or along one spacecraft track) might not give the same outcome as when on the opposite side of the planet, for instance due to the direction of the convective electric field, the relative location of the crustal magnetic fields (for Mars), or any shear flows in the solar wind. It also remains to be determined how long the system takes to return to pre-storm conditions. Solar minimum/maximum conditions can also affect how susceptible the system is to rapid changes in the upstream conditions. It is therefore important not to draw too strong conclusions from individual events from single-spacecraft measurements. There are also open questions on how and why Mars and Venus differ in their response to space weather.

### Bulk Escape via Coherent Plasma Structures

In addition to the escape of individual ions accelerated by, for example, the motional electric field (see Sect. 5.3), ions have also been observed to escape in somewhat discrete “blobs” of plasma, referred to as bulk escape. These blobs are typically characterized as plasma dominated by cold, heavy mass species of ionospheric origin, at relatively large densities up to ${\sim} 1000~\mathrm{cm}^{-3}$. Bulk escape has been observed at Venus and Mars, driven by a variety of processes. The PVO mission revealed decades ago the existence of plasma structures of ionospheric origin above the Venusian ionopause, known as plasma clouds, crossing the terminator region, traveling further downstream, and escaping the planet (e.g. Brace et al. 1982; Russell et al. 1982; Ong et al. 1991). Plasma clouds were observed at SZAs of up to $135^{\circ}$, outside the optical shadow of the planet, reaching altitudes of 2500 km above the terminator (Brace et al. 1982). Due to orbital restrictions that prevented a wide coverage of the deep nightside early in the mission, no plasma clouds were observed by PVO in the wake region in the early PVO studies. The typical densities of plasma clouds were of the order of $10^{3}\text{ cm}^{-3}$ with an average dimension of 1200 km along the orbit and an occurrence rate of 20% among the examined nightside orbits (Brace et al. 1982). Their distribution was suggestive of being a global phenomenon, as plasma clouds were observed by PVO both at dawn and dusk and at both northern and southern hemispheres of Venus at high latitudes (Brace et al. 1982). Estimations about accelerating mechanisms and their speed, as well as the eventual ionospheric loss via escaping plasma clouds demonstrated the significance of their contribution to the total atmospheric loss and hence the need for further investigation (Russell et al. 1982).

Russell et al. (1982) examined in detail such a plasma cloud seen in PVO’s orbit 601, utilizing additional magnetic field and plasma wave measurements. They found that the plasma cloud was associated with enhanced plasma wave activity similar to what was seen at the ionopause during the same orbit, and that in the center of the plasma cloud there was a reversal of the Bx component of the magnetic field (Russell et al. 1982; Ong et al. 1991). A decrease of 20 nT in the magnetic field magnitude was also observed, from which an estimation about the plasma thermal velocity was inferred, relating the origin of the plasma cloud to the ionosphere (Russell et al. 1982; Ong et al. 1991). Moreover, even though the plasma inside the cloud was hotter than the ionospheric plasma for the corresponding densities, its electron temperature was comparable to the values measured near the ionopause (Russell et al. 1982). Analysis of the magnetic configuration of the plasma cloud confirmed the escaping nature of them, as it was shown that they can accelerate to speeds greater than their escape velocity (Russell et al. 1982).

A study by Ong et al. (1991), based on the first 700 PVO orbits, further established three features (originally reported by Russell et al. (1982)), as the identifying signatures of plasma clouds: i) enhanced electron density above the ionopause, ii) enhancement in the plasma wave electric field intensity and iii) Bx reversal. Nonetheless, said features were not collectively seen at every plasma cloud detection. Only in 21% of the identified plasma clouds were all three signatures observed, whereas two or more signatures were detected in 67% of the cases. Consequently, the primary property for plasma cloud identification remained the enhanced electron density above the ionopause, and there were cloud identifications with only this feature present. Enhanced plasma wave activity and Bx reversals without being accompanied by electron density enhancements were also seen in the PVO orbits, and they were either correlated with ionopause crossings, or attributed to possible plasma clouds. The lack of electron density enhancements when magnetic and electric field features are seen -if they are indeed related to plasma clouds- could be attributed to either too low electron density to be detected by the Langmuir probes, or to plasma clouds being located at different altitudes but still connected to draped magnetic field lines (Ong et al. 1991). Nevertheless, through measuring the IMF orientation changes for each orbit, Ong et al. (1991) showed that there was a correlation between IMF rotations and plasma clouds, and that greater than average IMF rotations could be an indicator of plasma cloud formation.

At Mars, plasma structures resembling the plasma clouds seen at Venus were reported by Brain et al. (2010b), where the authors using MGS observations found detached magnetic structures containing ionospheric plasma downstream from regions of strong crustal magnetic fields. The crustal magnetic field lines were stretched due to their interaction with the solar wind downstream of crustal magnetic fields, where they could possibly get detached due to magnetic reconnection. The signatures of the detached magnetic structures were magnetic field strength enhancements, smooth rotations typical of flux ropes, and electron energy distributions indicative of ionospheric plasma. Furthermore, the angular distribution of the electrons appeared more isotropic near the center of the flux rope, compared to the surrounding regions, suggestive of electrons interacting with closed field lines forming detached plasma structures. The reconnection and detachment of the magnetic structures were additionally corroborated by MGS observations of current sheets between flux ropes and the corresponding crustal fields on the dayside, as well as by simulations of the solar wind - Mars interaction (Brain et al. 2010a; Harnett 2009). Finally, estimations based on the frequency of strong (∼120 nT) flux ropes from southern crustal fields seen in MGS data exhibited that the contribution of bulk removal of ionospheric plasma via detached magnetic structures could reach 5–10% of total ion escape (Brain et al. 2010b).

More recently, observations made by MAVEN demonstrated that heavy planetary ions can escape in discrete, coherent structures (“clouds”), driven by momentum transfer from shocked solar wind protons to the heavy ions (Halekas et al. 2016). This process, termed by the authors as a “snowplow effect”, is not the same as the magnetic reconnection driven formation of plasma clouds reported by Brain et al. (2010b): snowplow driven plasma clouds do not demonstrate significant twisting of the magnetic field, enhancements in plasma density precede enhancements in magnetic field, and the structures do not exhibit characteristics of force-free flux ropes. It was estimated that snowplow driven clouds may contribute 10–20% of the current ion escape rate from Mars. It is unclear if these bulk escape mechanisms are active at Venus: the apparent lack of crustal magnetic fields would suggest the preclusion of the process reported by Brain et al. (2010b); a lack of plasma measurements capable of resolving ion and electron distributions at suitably high time cadence at Venus, preclude conclusive identification there of the process reported by Halekas et al. (2016).

Low-altitude plasma clouds exhibiting different characteristics than the high-altitude clouds reported by Halekas et al. (2016), were detected by Zhang et al. (2021). The authors found plasma clouds at ∼600 km that originated from the low-altitude ionosphere (∼120 km). The magnetic field enhancements and twists, accompanying the low-altitude clouds, were possibly the result of different mechanisms than the high-altitude cases. Low-altitude clouds consisted of both planetary and solar wind plasma, with planetary ions of different masses travelling at the same velocity along open magnetic field lines, in contrast to the high-altitude plasma clouds in Halekas et al. (2016) that consisted of only planetary ions and were connected to draped field lines (Zhang et al. 2021).

MARSIS, the radar on board MEX, as well observed plasma structures at high altitudes in the Martian tail. During a special MARSIS campaign, where the radar sampled the nightside of Mars at altitudes up to 3500 km and SZAs that reached 180 degrees, plasma structures were consistently seen at the terminator region both at high and low altitudes with comparable densities. The densities near the optical shadow at 2500–3000 km were typically $50\mathrm{--}100~\mathrm{cm}^{-3}$ with occasional enhancements up to $400\mathrm{--}500~\mathrm{cm}^{-3}$. The presence of structures in the deep nightside magnetotail was more variable, where plasma structures do not appear to occur consistently at any given location. For SZAs of 160–180 degrees and altitudes between ∼600–1500 km the observed electron densities were of the order of $50\mathrm{--}100~\mathrm{cm}^{-3}$ (Stergiopoulou et al. 2020).

Several studies have identified the presence of the Kelvin-Helmholtz (KH) instability active along the Mars induced magnetosphere boundary (e.g. Ruhunusiri et al. 2016; Poh et al. 2021; Wang et al. 2022). The instability mixes solar wind protons and heavy planetary ions across the boundary, providing energy and momentum to the latter. The resulting KH vortices are characterized by bipolar-like sawtooth magnetic perturbations, and the co-existence of magnetosheath and planetary plasma. Magnetohydrodynamic simulations of the instability show that the resulting clouds of heavy ions can escape at rates that may contribute up to almost half of the current day ion escape rate at Mars (Wang et al. 2023a). Limitations in plasma observations at Venus (in particular, a lack of measured ion distributions at appropriate sampling cadence) prevent comparative detailed studies of the KH instability there (Pope et al. 2009), but a plethora of kinetic, MHD and hybrid simulations have demonstrated that it is likely also active and capable of producing similar clouds of escaping magnetosheath and planetary plasma (e.g. Wolff et al. 1980; Thomas and Winske 1991; Terada et al. 2002; Dang et al. 2022).

The typical structure of the magnetotail of Venus, as described by Brace et al. (1987) from PVO measurements, resembled filamentary cometary tails, with one central tail ray accompanied by one or more on each side. The tail rays could stretch out for thousands of kilometers, and had plasma densities of $50\mathrm{--}500~\mathrm{cm}^{-3}$. At the boundaries of the Venusian tail rays, signatures of magnetic field rotations suggested the presence of current sheets and the plasma pressure in the tail rays was found to be balanced by the magnetic pressure of the surrounding regions (Brace et al. 1987). The size of the tail rays, which was decreasing with altitude, was estimated ranging between 1000 km and 3000 km, whereas the width of the filaments was of the order of tens of km (Brace et al. 1987). Superthermal $\mathrm{O}^{+}$ ions with a velocity adequate for escape were observed at altitudes of 2000–2500 km, and the total $\mathrm{O}^{+}$ escape through the filamentary tail structure was estimated to $5\times 10^{25} \text{ s}^{-1}$ (Brace et al. 1987). Persson et al. (2018) showed, however, that the average escape rates of $\mathrm{O}^{+}$ range from 2 to ∼3$\times 10^{24}\text{ s}^{-1}$. Further modeling of the Venusian tail structure, inspired from the PVO observations, showed that there might be no special physical mechanism responsible for their formation and they could simply result from ion pickup by the convection electric field near the ionopause (Luhmann 1993).

**Fig. 23:**
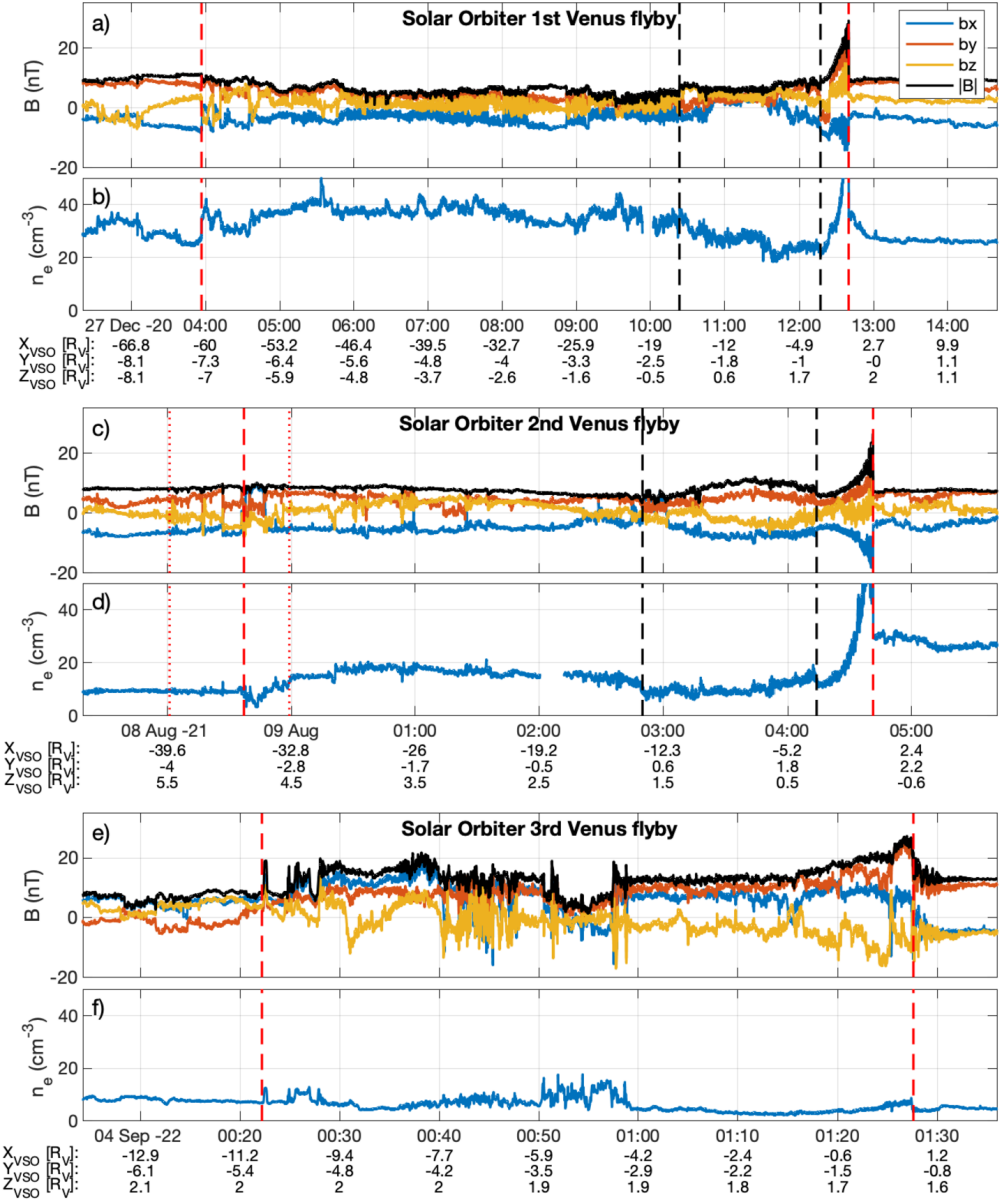
Magnetic field (B) and electron density ($\mathrm{n}_{e}$) measurements in the Venusian tail during the first three Solar Orbiter Venus flybys. Reproduced from Fig. 2 of Edberg et al. (2024a). Copyright 2024 by AGU, reproduced with permission

Owing to the PVO mission, the tail ray structure of the near tail up to a few Venus radii downstream of the planet was revealed. What happens in the far magnetotail and the length a tail ray can reach is not yet entirely clear. However, sporadic observations of the far Venusian magnetotail indicate the presence of planetary plasma at large distances from the planet. The Charge, Element and Isotope Analysis System (CELIAS) mass spectrometer experiment (Hovestadt et al. 1995) on board the Solar and Heliospheric Observatory (SOHO) (Domingo et al. 1995) spacecraft, in June 1996 detected $\mathrm{O}^{+}$ and $\mathrm{C}^{+}$ ions of densities $2.4\mathrm{--}4.4 \times 10^{3}\text{ cm}^{-2}\,\mathrm{s}^{-1}$, in the Venus wake at three occasions, at ${\sim} 4.5 \times 10^{7}\text{ km}$ downstream the planet (Grünwaldt et al. 1997). The energy distribution of the ions observed by SOHO was similar to Venusian tail-ray ions indicating the same origin (Grünwaldt et al. 1997). More recent measurements of spacecraft potential during the Solar Orbiter Venus flybys have also revealed electron density variability and enhancements, that particularly during the first Venus flyby reached values of ${\geqslant} 40~\mathrm{cm}^{-3}$ far down the tail at distances up to ${\sim} 50~\mathrm{R}_{V}$ from the planet, as it is demonstrated in Fig. 23, where magnetic field (B) and electron density ($\mathrm{n}_{e}$) measurements from the first three Solar Orbiter Venus flybys are shown (Edberg et al. 2024a; Stergiopoulou et al. 2023).

In addition, bursty fluxes of escaping heavy ions associated with the magnetotail current sheet have been observed at Mars and Venus by MEX and VEX missions (Dubinin et al. 2012). These bursts have periods of 1–2 minutes and the periodic structuring arises from the flapping motion of the tail current sheets. Large amplitude waves propagating along the current sheet surface, and magnetic reconnection in the tail, were postulated to drive these features. Bursty ion escape fluxes were also spotted at Mars with MAVEN (Dubinin et al. 2021). Fluxes of atomic and molecular oxygen ions, enhanced up to a factor of 100, were observed in the tail and near the terminator.

The tail-ray structure of the nightside of Venus, describing the pathways of the eventual ion escape first seen by PVO, was also investigated via hybrid simulations, demonstrating how the ambipolar electric field, formed by the electron pressure gradient, is the driver for the formation of tail rays (Brecht and Ledvina 2021). The ion densities seen in their simulation results agreed with the PVO observations made decades before. The simulation results revealed the formation of one tail-ray structure the location of which was offset from the midnight direction. Through additional analysis the authors discovered that the IMF orientation is one of the factors controlling the location of the tail ray. After combining results from PVO, VEX, a hybrid model and PSP Venus flybys, a revised structure for the magnetotail of Venus was suggested that consisted of only one tail ray, which reaches at least 7800 km altitude downstream Venus (Collinson et al. 2021). PSP during its fourth Venus flyby crossed the planet’s tail between 5364 and 7810 km altitude, evidently passing through the tail ray, and measured densities of ${\sim} 47~\mathrm{cm}^{-3}$ of cold planetary plasma (Collinson et al. 2021). The plasma and magnetic field measurements from PSP Venus flyby generally agree with PVO observations reported by Brace et al. (1987). Collinson et al. (2021) taking into account PSP data and the results of the hybrid simulation by Brecht and Ledvina (2021) suggested the existence of one central tail ray in the nightside of Venus, arguing that previous observations of several tail rays resulted from multiple crossings of a single highly dynamic tail ray. Moreover, bursty escape fluxes reported in Dubinin et al. (2012) could also be describing tail rays, where the variability is explained by the flapping of the current sheet.

A comparison of the structures present in the Martian induced magnetotail with what PVO had seen travelling in Venus’s wake, was made possible after Phobos 2 provided plasma and magnetic field measurements from the optical shadow of Mars (Dubinin et al. 1991). PVO and Phobos 2 data showed that Mars and Venus magnetotails look similar i.e. two tail lobes with a central current sheet. However, unlike the PVO observations, plasma structures or tail rays on either side of the central current sheet, were only occasionally detected at Mars (Dubinin et al. 1991). A tail-ray structure has not been identified at Mars by the more recent missions such as MEX and MAVEN, although there have been detections of high altitude plasma structures as described above (e.g. Brain et al. 2010b; Halekas et al. 2016; Stergiopoulou et al. 2020) and of escaping bursty fluxes (Dubinin et al. 2012, 2021). Neither did hybrid simulations find a tail ray in the wake of Mars, possibly due to the peak ambipolar electric field being five orders of magnitude smaller than the corresponding value at Venus (Brecht and Ledvina 2021). However, tail rays are included in hybrid simulations when they coincide with the tail plasma sheet (Boesswetter et al. 2009; Wang et al. 2024a). In addition, the maximum value of the electric field of the hybrid simulation for Venus occurs below 400 km, in a region where the plasma density is high, whereas the peak electric field at the Mars case was found at higher altitudes and lower plasma densities (Brecht and Ledvina 2021).

The discovery of escaping structures, leaving the planets via magnetotail paths, brings about additional questions concerning their nature and features. For example, even though the plasma clouds appear as they are detached, we cannot distinguish, utilizing past and current measurements as they are all single point measurements, whether they are truly disconnected from their surrounding environment. Furthermore, there are no observations at the moment that could help us definitively tell apart structures such as clouds and tail rays, if they are indeed separate phenomena and not simply different manifestations of the same structure. In addition, the response of said structures to changing upstream conditions and space weather events has not been thoroughly investigated due to the lack of two-point simultaneous measurements. Finally, plasma instabilities and kinetic scale processes, which may drive the formation of the observed structures at the nightside of Venus, have not yet been described, as existing ion measurements made by PVO and VEX did not measure the full ion distribution functions at adequate sampling cadence.

## Other Induced Magnetotails and Datasets

### Titan

**Fig. 24:**
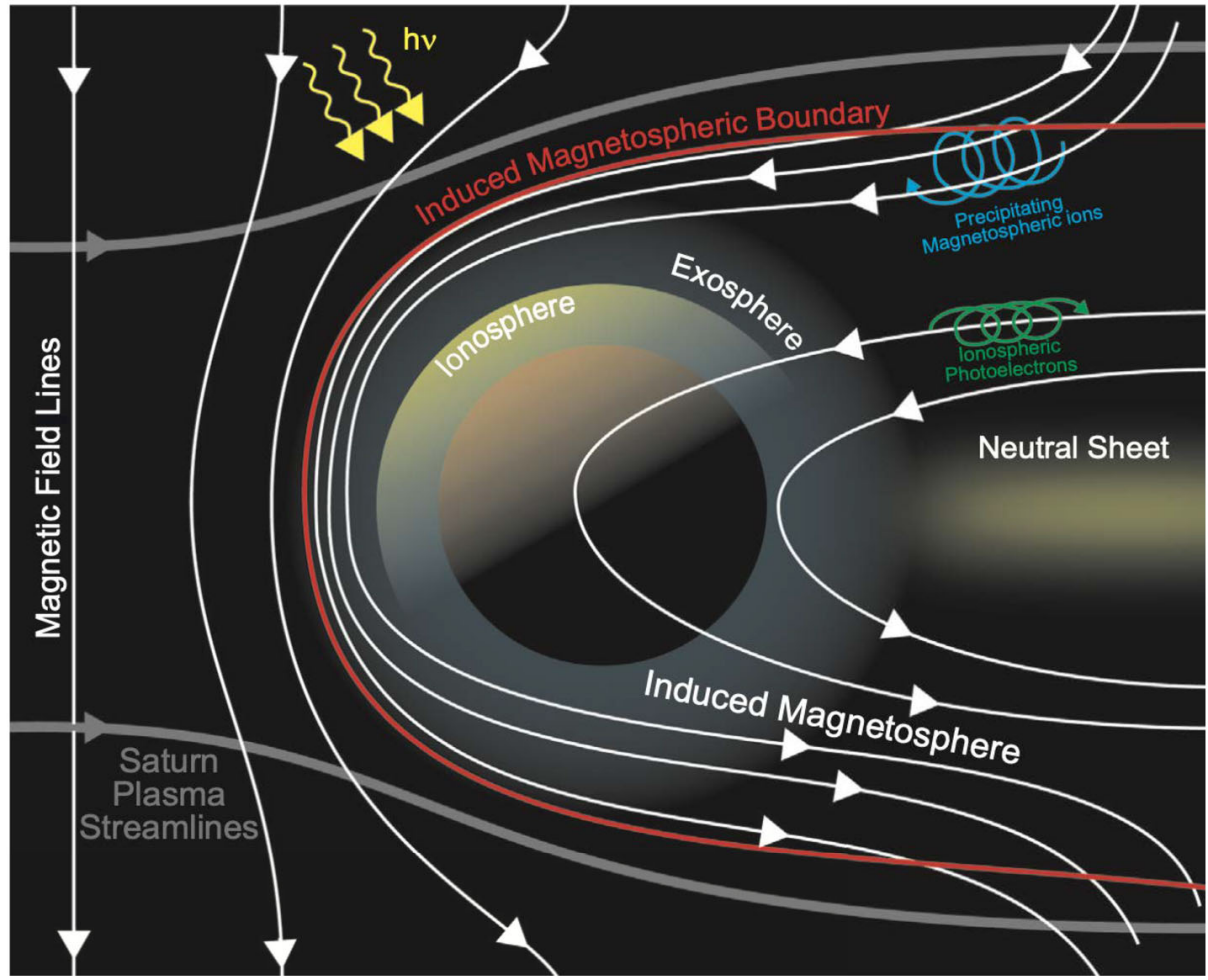
Schematic view of Titan’s plasma environment, with some of the key regions, boundaries and physical processes indicated. Reproduced from Fig. 26.1 of Bertucci (2021). Copyright 2021 by AGU, reproduced with permission

Titan is another well-studied body in the solar system with another type of magnetotail. Even though Titan is not at the focus in this paper, it is interesting to compare how different unmagnetized bodies can interact with the ambient plasma in markedly different ways, resulting in magnetotails with distinct characeristics. The Cassini spacecraft (Matson et al. 2002) performed 126 close flybys of Titan from 2004 until 2017, and collected measurements of the plasma and magnetic field environment on almost all passes. Titan is somewhat smaller than Mars ($\mathrm{R}_{T} = 2575\text{ km}$ vs. $\mathrm{R}_{M} = 3390\text{ km}$) but has a dense and very extended ionosphere (ionospheric peak at ∼1200 km compared to ∼140 at Mars and Venus) leading to a relatively larger plasma environment and magnetotail. A schematic view of Titan’s plasma environment is shown in Fig. 24. Titan is different from Mars and Venus in that the upstream plasma is not the solar wind but rather the co-rotating plasma within Saturn’s magnetosphere.

Because Titan is submerged in Saturn’s magnetosphere, the angle between the incident plasma flow and the Sun’s direction changes with the local time; therefore, the plasma wake and nightside wake at Titan are decoupled. Depending on the orbital phase of Titan, this decoupling introduces dayside-nightside asymmetry in the ionization rates with the solar UV dominating the dayside (Ågren et al. 2009; Vigren et al. 2013; Shebanits et al. 2017), and impinging energetic magnetospheric particles on the nightside (Cravens et al. 2009; Vigren et al. 2015). Furthermore, because Saturn’s plasma sheet at Titan’s orbit is bent due to the oblique pressure exerted by the solar wind, (Arridge et al. 2008; Bertucci et al. 2009) Titan is periodically (with the period of Saturn’s rotation) exposed to the high or low plasma β regions of Saturn’s magnetosphere, i.e., magnetospheric lobes and plasma sheet, respectively (Simon et al. 2010a,b; Arridge et al. 2011; Kabanovic et al. 2017). When Saturn’s magnetosphere is compressed due to increased solar wind pressure, Titan can be exposed to a perturbed magnetosheath plasma and even the solar wind (Bertucci et al. 2008; Wei et al. 2011; Edberg et al. 2013; Bertucci et al. 2015; Feyerabend et al. 2016; Burne et al. 2023). Thus, Titan’s plasma interaction pattern and upstream plasma parameters change depending on the relative location of Titan to Saturn’s plasma sheet. These processes lead to the complex physics of the Titan magnetotail.

An intrinsic magnetic field has not been detected at Titan and, if it exists, is rather weak (Backes et al. 2005). Therefore the incident plasma, similar to Mars and Venus, interacts almost directly with the ionosphere (Bertucci et al. 2011). The proton gyroradius in the upstream region is smaller than Titan’s radius. At the same time, the oxygen and water group ions have larger gyroradius (Ledvina et al. 2005). The interaction of Saturn’s magnetospheric plasma with Titan’s ionosphere is thus hybrid, where both kinetic effects (e.g., plasma collisions and instabilities) and large-scale dynamics (e.g., plasma flows, electromagnetic field environment) are important. While the solar wind flow is often super-Alfvénic and supermagnetosonic, Saturn’s magnetospheric plasma at Titan’s orbit corotates with submagnetosonic velocities. As the average upstream region of Titan is submagnetosonic (but super-Alfvénic) (Arridge et al. 2011), a fast magnetosonic mode is not excited and a bow shock is not formed. Slow magnetosonic and Alfvén waves are excited instead. However, when Titan was found in the solar wind, a bow shock was observed as a part of the deformed Saturn bow shock (Omidi et al. 2017).

The incident plasma flow is mass-loaded on the ramside of Titan, slowing down and deflecting around Titan. The magnetic field is frozen-in into the plasma flow and thus bent around Titan and stretched downstream. However, compared to Mars and Venus, these bent-around magnetic field lines do not create an extended region of confined anti-parallel field lines separated by the cross-tail current sheet. Instead, two separated magnetotail lobes are observed in the far-tail region and a Mars/Venus-type magnetotail structure in the near-tail region. The degree of confinement depends on the ambient plasma, β, ionospheric conductivity, and the Alfvén Mach number, mediating the strength of plasma interaction (Simon et al. 2015; Saur 2021). The discussed magnetotail of Titan is reminiscent of the so-called Alfvén wings and is a distinct feature of a subalfvenic moon-plasma interaction. The separation of the magnetotail lobes is referred to as a ‘split’ signature and is unique to Titan (Bertucci et al. 2007; Wei et al. 2007; Coates et al. 2012). During the TA, TB, and T3 flybys of the Cassini spacecraft, Backes et al. (2005) analyzed the structure of Titan’s magnetotail and compared observations with the hybrid simulation. They found that the magnetotail is confined to a narrow region. The analysis of plasma measurements in distant tail flybys (T9, T63 and T75) by Coates et al. (2012) showed that the split signature manifests itself in the form of filaments filled with escaping ions and electrons, originating from Titan’s ionosphere. The asymmetry in the electron escape rate was explained by the magnetic connection of filaments to the sunlit and nightside ionosphere of Titan by using multi-species hybrid simulations (Modolo et al. 2007; Kallio et al. 2007; Feyerabend et al. 2015). The summary of mid-range magnetotail flybys from TA to T82 by Simon et al. (2014) has concluded that the transition between the northern and southern magnetotail lobes occurs at a distance of ${\leq} 2.5~\mathrm{R}_{T}$ (1 Titan radii $\mathrm{R}_{T} = 2574.7\text{ km}$). Kim et al. (2024) looked at the last five flybys (T122–T126) and found that some of the observed parameters of the magnetic field are consistent with Alfvén wings. In addition, Kim et al. (2024) summarized all the available Cassini measurements of the magnetic field and mapped the cross-section of Titan’s magnetotail. In the cross-section, the magnetotail was confined to a region of approximately ${\sim }4~\mathrm{R}_{T}$.

The detailed physical processes in the induced magnetotail of Titan were investigated during the Voyager 1 and Cassini flybys. The induced magnetotail of Titan was first detected in a Voyager 1 flyby (Ness et al. 1982; Hartle et al. 1982). The analysis of electric field fluctuations revealed the presence of an upper-hybrid emission line and broadband electrostatic noise (Gurnett et al. 1982). The upper-hybrid resonance frequency is a natural wave mode in magnetized plasmas and is used to determine local electron density. The broadband electrostatic noise was related to the active generation of pick-up ions and had signatures of dayside-nightside asymmetry. Later with Cassini, the studies focused on the outflow processes and plasma acceleration mechanisms. In general, escape mechanisms can be thermal, e.g., sputtering, hydrodynamic escape, and non-thermal, e.g., pick-up by the upstream electromagnetic fields, ambipolar electric field (the comprehensive review of these processes can be found in Johnson et al. (2010), Wahlund et al. (2014)). It was found that ionospheric photoelectrons escape along the magnetic field lines down to the magnetotail. When escaping, photoelectrons drive an ambipolar electric field which causes the pressure-driven escape, similar to the polar wind mechanism at Earth (Coates et al. 2007, 2015). The outflow rate of plasma through the magnetotail of Titan was estimated in several studies and was found to vary significantly depending on the ion species (Coates et al. 2015). The bending of the magnetic field lines in Titan’s magnetotail causes the magnetic tension forces to arise. This force exerts $\vec{j}\times \vec{B}$ on plasma and accelerates plasma tailward, leading to an escape (Israelevich and Ershkovich 2014; Romanelli et al. 2014). In Edberg et al. (2011a), such escape processes were proposed to be responsible for a continuous outflow observed in the tail through plasma measurements. The candidates responsible for the outflow are magnetic pumping, ambipolar electric field and/or conversion of Alfvén waves into dispersive Alfvén waves. In reality, it is hard to determine the exact escape and acceleration mechanisms, but the discussion above gives us a grasp of the possible processes in Titan’s magnetotail. Another difference between Titan and Mars and Venus is the presence of heavy negative ions in Titan’s ionosphere and their possible impact on the plasma escape rates, and general plasma interactions (Ledvina and Brecht 2012).

Despite the significant progress in our understanding of Titan’s magnetotail structure, the physical processes governing the dynamics of plasma in the magnetotail are under-explored. The energy conversion processes in Titan’s magnetotail, such as magnetic reconnection and plasma instabilities, might significantly contribute to the energy dissipation and overall dynamics of the magnetotail. The variability of Titan’s magnetotail with the solar cycle, orbital phase, and upstream magnetospheric conditions (e.g., magnetic field strength, solar EUV flux, upstream energy flux) is not yet explored due to the limitations of Cassini’s measurements but can be a subject of computer simulations. Finally, currents in the magnetotail and their contribution to the global current system, and coupling via electromagnetic fields of Titan’s plasma environment (i.e., ionosphere, induced magnetosphere) with Saturn’s magnetosphere, should also be discussed in the future.

### Comets

Comets are different from moons and terrestrial planets in several ways, which leads to their plasma tail structures being also rather different. The atmosphere (coma) of a comet is created when the ice in the nucleus heats up by the Sun and sublimates. This process is strongly dependent on the heliocentric distance and the intensity of the sunlight, such that comets in their elliptical orbits can have their coma grow in size by many orders of magnitude from aphelion (where they might have no coma at all) to perihelion. While planets like Mars and Venus have atmospheres bound by gravity, comets are small enough that any material that sublimates and outgasses, or otherwise leaves the surface, expands freely into space. This means that cometary plasma environments can grow to become some of the largest celestial plasma environments in the solar system when they reach the inner solar system (Edberg et al. 2024b).

**Fig. 25:**
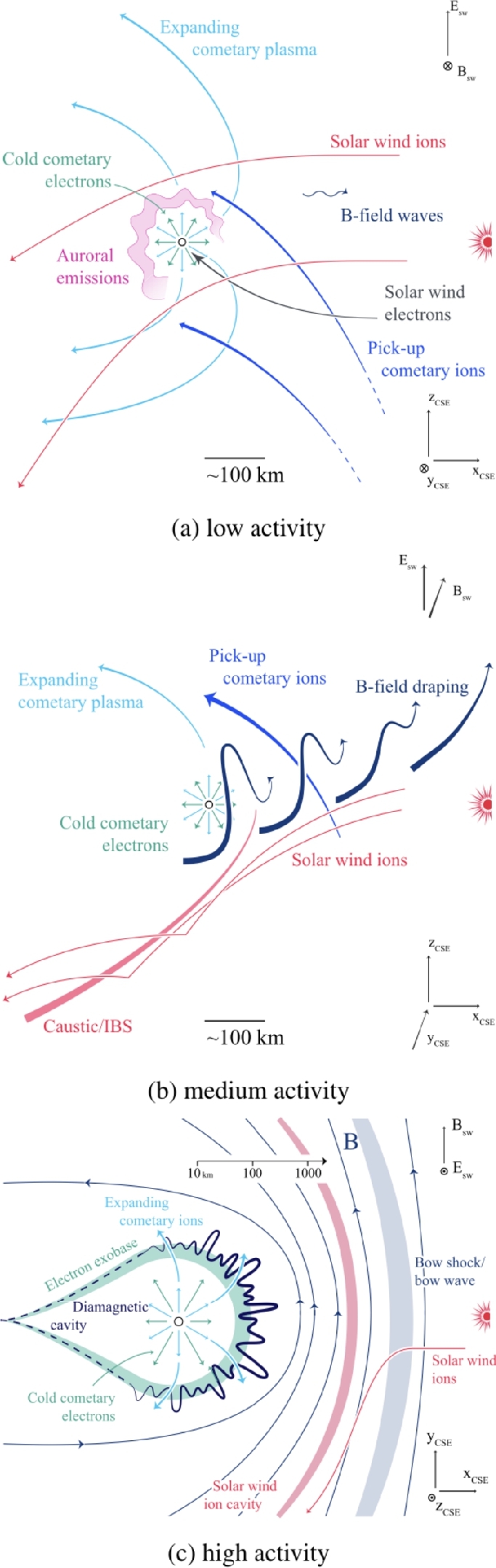
Comet plasma environment at different activity levels, going from a low outgassing case in (a) to medium activity in panel (b), and finally high activity in panel (c). Different regions and boundaries emerge as the activity increases, and different physical processes gain in importance. Reproduced with permission from Goetz et al. (2022), copyright by the author(s)

Each comet is different and largely defined by their individual outgassing rate. At 1 AU, the outgassing rate of comets are typically in the range $10^{24}$–$10^{30}$ molecules/s (Goetz et al. 2022), but can in extreme cases be outside of this interval. Weakly active comets (outgassing closer to $10^{24}$ at 1 AU), or comets far from the Sun, develop rather weak tails characterised by low plasma density, and are governed by kinetic effects (Behar et al. 2017), while more active comets, such as comet 1P/Halley, gradually transition into the MHD regime (e.g Rubin et al. 2014). A schematic view of how a comet transitions from a low activity to a high activity comet was presented by Goetz et al. (2022), shown here in Fig. 25. As a comet interacts with the solar wind, various boundaries and regions form (Mandt et al. 2016), where the outermost one is the bow shock, that only begins to form when the outgassing rate has reached some threshold (Gunell et al. 2018).

Comets typically carry two tails: one plasma tail composed of ionised particles directed approximately in the anti-sunward direction, controlled by the solar wind and IMF, and one dust tail composed of dust grains, which is roughly trailing the comet, i.e. in the opposite direction of the comet velocity vector, but often in a slightly bent way since it is affected by the Sun’s radiation pressure on the dust grains combined with the trajectory of the comet.

While several spacecraft have visited comets, only two have performed in situ measurements of their tail regions. The very first comet mission, the International Cometary Explorer (ICE, Ogilvie et al. (1977)), performed a flyby of comet 21P/Giacobini-Zinner in 1985 and could observe the central current sheet at a distance of about 7800 km from the nucleus (Slavin et al. 1986). The second spacecraft was Rosetta (Taylor et al. 2017), which moved around comet 67P in semi-bound orbits typically within 100 km’s in the terminator region, but made one excursion to a few hundred kilometers in the downtail/flank direction (Volwerk et al. 2018). At this time, comet 67P was at 2.7 AU from the Sun and rather inactive. The cometary ionospheric plasma density close to the nucleus (at least within 1500 km) was found to decrease linearly (Edberg et al. 2022). Behar et al. (2018) similarly found that the cometary ion motion was close to radial during the entire excursion, out to a few hundred km from the nucleus. The governing forces at this time were suggested to be a combination of electric field arising from the ion motion, the electron pressure gradient and the magnetic field bending. Furthermore, no classic bi-lobe tail structure was observed in the visited region. The main magnetic field direction was rather found to be more across the tail direction, perhaps due to an asymmetric ion-pick up (Volwerk et al. 2018). The magnetic observations were at times dominated by high-amplitude waves. Cometary magnetotails are hence rather unexplored, and expected to be quite different depending on which comet is considered.

Only a handful of comets have been observed in situ, thus many questions remain on how their tails begin to form when far from the Sun and how they evolve when very close to the Sun. Few measurements overall have been conducted in the far tail region of comets which makes this a more or less unexplored region. Which boundaries are present for comets with high outgassing rates and which are present for low outgassing rate comets? What physical process in the tail are at play when so-called tail disconnection events occur? How do the various plasma boundaries evolve with heliocentric distance/outgassing rate, especially when they eventually grow to sizes which, in theory, could even reach the Sun?

### Exoplanets

Within the studies of exoplanets, the majority of efforts have been concerned with the studies of magnetized planet interactions with the local stellar environment. This is, primarily, owing to the possibility of the remote detection of such an interaction at radio frequencies (e.g. Zarka 2007). However, in this rapidly-evolving domain, recent observations combined with modelling studies, indicate the possibility of detection of extended escaping planetary atmospheres, and hence modification of these escaping plasma structures in and through the magnetospheric tail is now an observable property of some systems (e.g the discussion provided in McCann et al. 2019). Given the observational biases present in the catalog of known exoplanetary analogues to the terrestrial planets, there is some reason to suspect that due to their likely tidally-locked and slow rotation, active dynamos may not be common features of these systems. It might therefore be suspected that an induced magnetospheric interaction is more typical for these objects (Canet et al. 2024). Indeed, even larger close-in exoplanets of the hot-jupiter class may not posses significant intrinsic fields and therefore exhibit an induced magnetospheric interaction (Erkaev et al. 2017). Furthermore, observational constraints generally favour the detection of terrestrial exoplanets around lower-mass stars such as M-dwarfs, which are typically highly variably in regards to their flaring activity and consequently exposing these planets to wider extremes of driving conditions (Airapetian et al. 2020). Thus, it is imperative to progress in our understanding of the induced magnetotails present in the solar system that can be systematically surveyed through in-situ and remote observations, under varied driving conditions, to fully constrain theories and approaches used in the analysis of analogues observations beyond the solar system.

## Open Questions and Future Needs

Investigating induced magnetospheres allows us to sample and analyze different parameter regimes to magnetized planets that are not necessarily accessible at Earth. Additionally, unmagnetized planets are highly dynamic, driven by the solar wind interaction, meaning they contain a rich and diverse set of physical processes. Numerous successful plasma missions have probed Mars and Venus and revealed their different – compared to the other Solar System planets – induced magnetospheres and the physical processes therein, highlighting the importance and necessity of planetary plasma missions. However, induced magnetotails are often regarded as less important in controlling a planet’s interaction with its near-space environment, especially when compared to the extended energy-storing tails of intrinsic magnetospheres. This review has sought to demonstrate that consideration of formation, dynamics and variability of induced magnetotails is needed in order to fully understand the near-space environments of un- or weakly-magnetized planets. In this section, we briefly summarize the limits of our current understanding on the topic, and address the measurement needs that remain. In Table 3, a summary of the remaining open questions described in each section, as well as suggestions on ways to address them is presented.

**Table 3:**
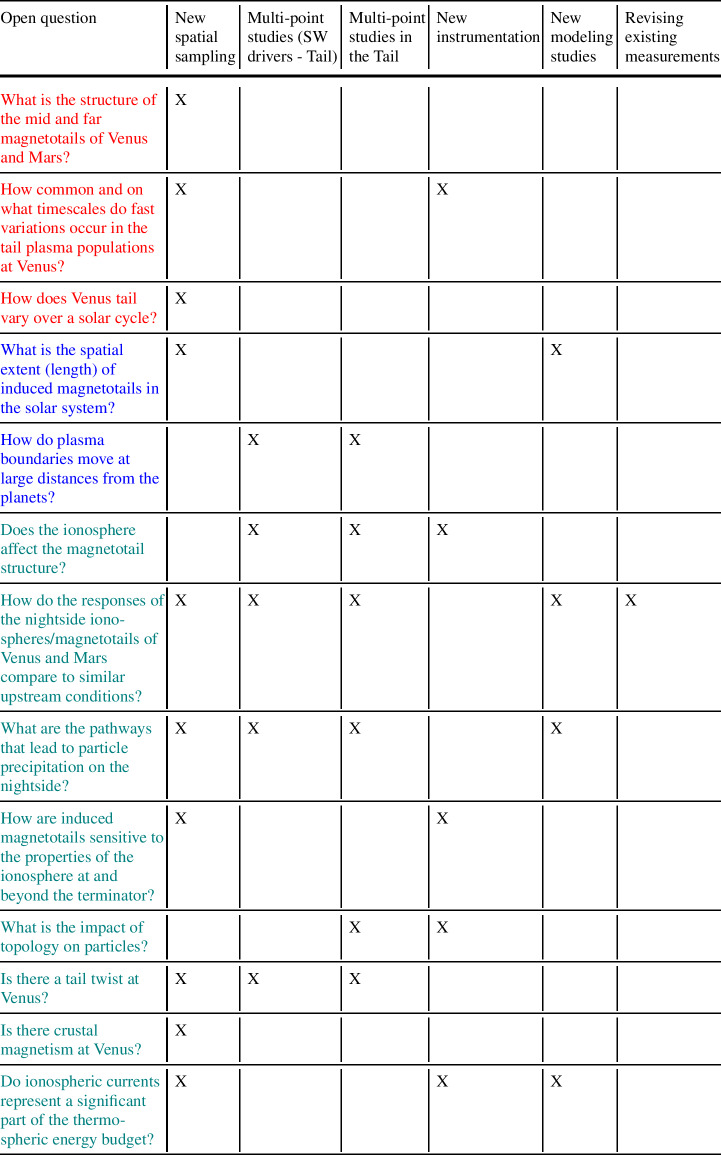

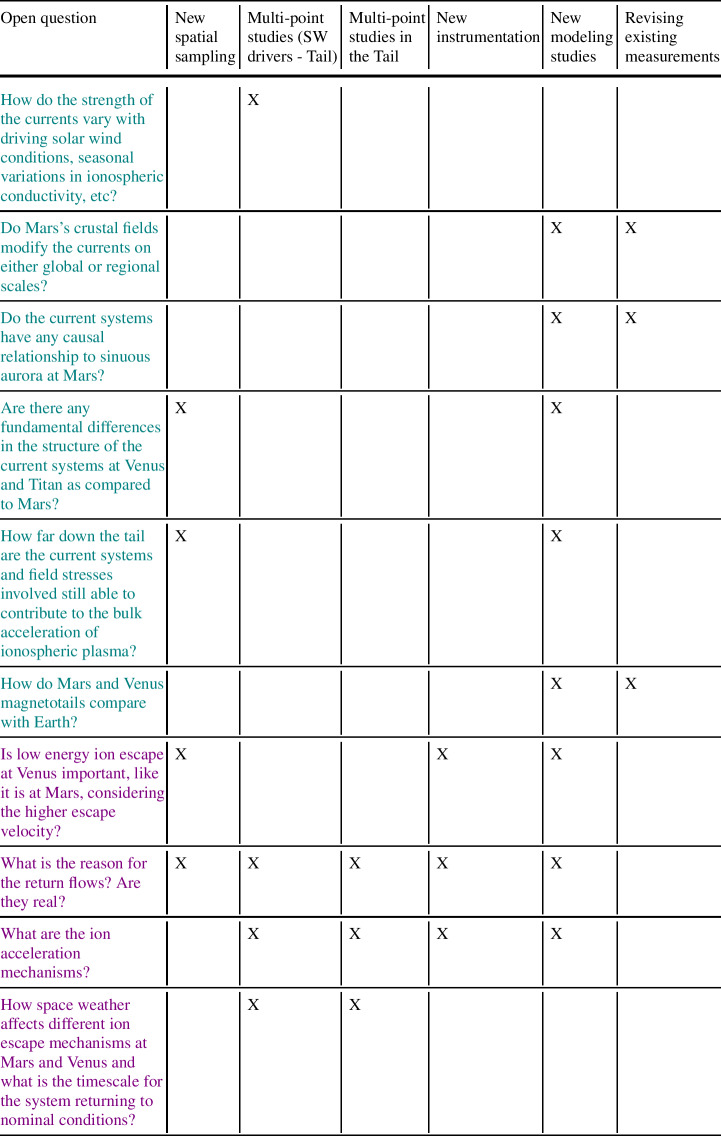

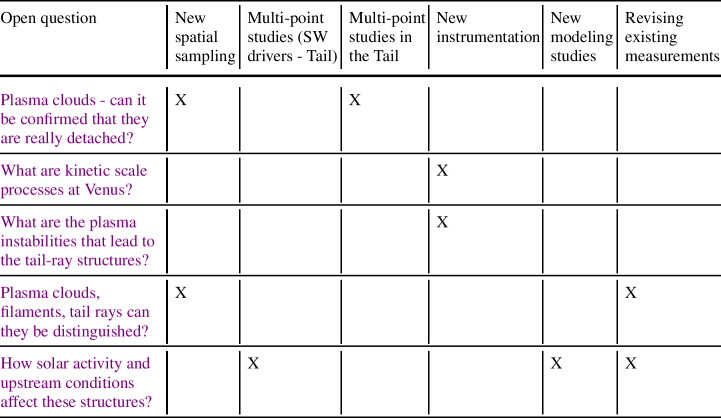
Open questions/topics summarized and ways that they could be addressed. Each section’s questions are depicted in a different color: , , ,

Both observations and simulations have proven to be effective tools for demonstrating the details and differences in the plasma boundaries between unmagnetized or weakly magnetized planets and those with intrinsic magnetospheres. Thanks to planetary plasma missions and tail flybys, the tail structure of Mars and Venus started to become clearer and the comparison between the two planets has become feasible. Ion escape rates have been estimated, and ion acceleration mechanisms and bulk escape for the two planets as well as the impact of space weather on them have been detected and described. However, they are still not fully understood -we lack dedicated multi-point measurements at both planets that would monitor the upstream solar wind, and response of the magnetosphere, in real time. We know that induced magnetospheres are formed not only around Mars and Venus, but also around other celestial bodies with gravitational bound atmospheres, such as Titan, presumably exoplanets, and even comets. A comparison between different Solar System bodies, besides the better understanding of the Solar System, can contribute to analyzing the plasma environment of exoplanets. Currently, there are no plasma missions orbiting Venus. Future planned ones (DAVINCI - Garvin et al. 2022, VERITAS - Smrekar et al. 2022, Envision - Bruzzone et al. 2020) will be focused on surface physics and will not probe the plasma environment and the interaction with the solar wind. Yet, there are several spacecraft at Mars, operated by different space agencies, carrying plasma and fields instruments, while future missions include ESCAPADE (launched in 2025 - Lillis et al. 2022a) and MMX (due for launch in 2026 - Campagnola et al. 2018). Additionally, Dragonfly, a NASA mission that will travel to Titan, is expected to be launched in 2028, although there will be no plasma instrumentation onboard (Lorenz et al. 2018).

Nevertheless, the majority of the remaining open questions about the plasma environment in the induced magnetotails of Mars and Venus would require multi-point observations and hence coordinated multi-spacecraft missions. Thus far, no coordinated campaigns between missions coming from different space agencies have been conducted, the orbits of the different spacecraft are not usually complementary, and the various missions consist of different instrument suites.

Despite the progress of the last decades, there are still unexplored regions such as the low altitudes and low latitudes at Venus, and the mid and far magnetotails of Venus and Mars. As PVO covered only low latitudes and VEX sampled only the north pole, full ionospheric coverage and full solar cycle coverage of both ionosphere and magnetotail are needed. Future Venus missions should also aim to acquire full energy spectra of ion escape in its induced magnetotail, and high time resolution ion spectrometer measurements in the near-to-mid magnetotail. MAVEN, which carried modern plasma instrumentation capable of measuring escaping ions down to low escape energies, has shown the importance of acquiring measurements near this lower limit. Furthermore, Venus missions should consist of comprehensive plasma instrumentation that will be capable of resolving ion and electron kinetic scale physics, which requires measurements of ion and electron distribution functions at time cadences <4 s, since plenty of processes occur at this time scale, as it has been shown by MAVEN. The real limitation in that approach will actually lie on the telemetry bandwidth i.e. how much data can be sent back to Earth. Thus, increased capability in telemetry transmission will be also needed to support such future missions. As it has been stated before, the induced magnetotails of Mars and Venus are highly dynamic and responsive to upstream drivers. Consequently, the next goals for the two planets should be i) instantaneous upstream and magnetotail measurements, and ii) two-point measurements in their tails, both of which are feasible via two- or multi- spacecraft missions.

Measurements in the extended distant magnetotails are rare throughout the solar system, owing to orbital constraints. These poorly explored regions can represent high scientific value targets of opportunity for interplanetary missions during cruise phases, e.g. when using gravitational assist maneuvers, such as Solar Orbiter’s and Parker Solar Probe’s Venus tail encounters. Several open questions remain regarding the plasma boundaries of the magnetotails of Mars and Venus; to what distance from the planet the induced magnetotails extend and how does this compare to Earth’s magnetotail? What are the characteristics of boundary motion, including the velocity and variability of these plasma boundaries over time and what are the drivers of said variability? To address these issues new multi-point observations are necessary combined with plasma boundaries simulations.

How does the nightside ionosphere at Venus respond to changes compared to Mars? MEX and MAVEN have demonstrated the dynamic behavior of the Martian nightside, driven by magnetotail physics. However, our understanding of it is limited due to the lack of dedicated multi-point measurements. We know even less about the Venusian nightside and magnetotail as there is less data available compared to Mars. Mars Express and MAVEN have paved the way for studies on this topic, while other missions, for example the Emirates Mars Mission (Amiri et al. 2022), have provided compelling information on the auroras. By comparison there is limited data from Venus, although the presence of structured high altitude ionization has been found. What are the pathways that lead to particle precipitation on the nightside? It is clear that particle precipitation into the Martian ionosphere is responsible for creating some types of aurora as well as lower altitude ionospheric layers. Nevertheless the exact pathways for these particles to enter into the ionosphere and the mechanisms responsible remain unclear. Is there evidence for a change in the structure of the tail with distance from the planet? We have limited observations at large distances downstream in the magnetotails of Mars and Venus, certainly at distances greater than 10 planetary radii. Numerical simulations provide a hint of the structure but with the relative lack of observations, these simulations are difficult to confirm.

How does the ionosphere and the temporal solar wind and IMF variability affect an induced or hybrid magnetotail? Are there any similarities to Earth? For example, MAVEN observations revealed the presence of a magnetotail twist at Mars, the direction of which depends on the IMF dawn-dusk polarity, indicating that the solar wind - Mars interaction is more Earth-like than previously thought. Thus far, there has not been any report of a Venusian magnetotail twist. Further studies are needed to definitively determine whether there exists a magnetotail twist in the Venusian magnetosphere and if there is any equivalent phenomenon to crustal magnetization of Mars.

Magnetic topology has been inferred from superthermal electron spectra and magnetic field measurements for both Mars and Venus. Nevertheless, key questions remain concerning the influence of magnetic topology on ion escape: how do the forces that drive ion energization and subsequent escape to space vary over different magnetic topologies, and how are these topologies (and the ionospheric source regions for escape that they can access) impacted by upstream conditions? Moreover, electromagnetic waves in the magnetotails of Mars and Venus have received relatively low attention. We do not know much about their generation processes, their impact on the magnetotail, and energy transfer processes to the nightside ionosphere.

The large-scale structure of the magnetic fields of induced magnetospheres was inferred from PVO observations at Venus (Saunders and Russell 1986). The availability of a large set of observations throughout near-Mars space allowed the direct determination of the associated current systems. However, open questions remain about their nature. Ionospheric currents that couple the induced magnetosphere to the ionosphere and upper atmosphere are expected to deposit energy through Joule heating. To what extent does this contribute to the thermospheric energy budget in key regions? How does the strength of these currents vary with solar wind driving conditions, seasonal variations in ionospheric conductivity, and other influencing factors? To what degree are these currents modified by Mars’s crustal magnetic fields, either on global or regional scales? Is there a causal connection between these current systems and the sinuous auroral structures observed at Mars? Are there fundamental differences in the structure and behavior of such current systems at Venus and Titan when compared to Mars? And finally, how do these systems compare to similar ionospheric current systems observed at Earth. To find answers to all the above questions, multi-point plasma measurements are necessary.

Ion escape has been extensively investigated at both Mars and Venus. There have been estimates of heavy ion escape rates and they have been compared with those at Earth. The physical processes on which ion escape depends have also been studied. However, there are still unresolved issues concerning ion escape at unmagnetized planets. Is low energy ion escape, which is important at Mars, crucial at Venus too, considering the higher escape velocity? What causes the observed return flows at Venus? What are the ion acceleration mechanisms that allow ions to surpass the escape velocity at Venus and what is the real effect of the solar wind and space weather on atmospheric evolution? Which are the primary atmospheric constituents that escape through different mechanisms? For example, carbon and helium is of interest at Venus, whereas at Mars the contradiction between oxygen and hydrogen escape is most intriguing. A full characterization of all potential escape channels for the atmospheric evolution, i.e. ion escape in magnetotail/magnetosheath, sputtering, photochemical escape, thermal escape etc., as some of the pathways are not fully explored, is needed for both planets to provide the full picture to the atmospheric evolution.

Past observations have demonstrated that the magnetotail is an important region where bulk escape of ionospheric plasma can occur in the form of coherent plasma structures, perhaps contributing 10 s of percent to the current day ion escape rates of Venus and Mars. However, limited orbital coverage and limitations with instrumentation mean that much is still unknown about both the characteristics and driving processes of these structures at Venus. The escaping plasma structures appear as regions of elevated electron density surrounded by essentially absence of plasma, which gives the impression of them being detached, but in reality this may only be due to the limited view from the spacecraft’s trajectory. Existing measurements cannot conclusively determine the true spatial extent of these structures, or even confirm that they are truly disconnected from the ionosphere. The lack of such knowledge makes it difficult to conclusively identify these various structures (plasma clouds, tail rays, filaments) as separate phenomena, as opposed to different manifestations of the same structure. Are these tail structures separate phenomena and what are the distinguishing features between them? To confirm whether certain plasma structures are detached at the moment of their detection, and to determine their paths, as well as where and how the detachment occurs, multi-point measurements throughout the magnetosphere are necessary. In addition, new focused magnetotail observations combined with existing data will help in better defining and distinguishing the observed plasma structures.

The response of the Venusian tail structure and characteristics, as a function of upstream solar wind and space weather conditions, is poorly constrained; while recent global hybrid simulations have shown the magnetotail structure to be highly responsive to these drivers, the lack of dedicated multi-point measurements at Venus makes it difficult to confirm these predictions. Finally, plasma instabilities and kinetic scale processes, which may drive the formation of these magnetotail structures, cannot be observed with necessary fidelity by current measurements, and their roles remain unknown to us. More recent ‘observations of opportunity’ (i.e. SolO, PSP, BepiColombo flybys) have revealed tantalizing hints at what a modern, comprehensive plasma mission may reveal at Venus, but dedicated multi-point orbiter missions targeting the two planets are required to fully unravel the complexities and evolution of these escaping structures.

The research on the induced magnetotails of other bodies in the Solar System, such as Titan, has been relatively limited so far, with several open questions yet to be addressed. What are the exact mechanisms of the non-thermal escape and their relative contribution to the total escape? What are the plasma processes governing energy conversion in Titan’s magnetotail? How does Titan’s magnetotail couples electromagnetically to Saturn’s magnetosphere and how important its role is in the global dynamics of Saturn’s magnetosphere? What are the main drivers of spatio-temporal variability in Titan’s magnetotail? Many questions about Titan’s magnetotail remain unanswered due to the lack of observations and limited coverage of the region. Cassini made 126 flybys, and only a fraction of these flybys occurred in the magnetotail, both far-, mid- and near regions. There are also constraints imposed by the plasma instruments onboard Cassini that suffered from multiple sources of interference and perturbations. Titan dedicated future missions, with improved plasma (high cadence ion and electron distribution functions spanning thermal to suprathermal energy ranges) and 3D electromagnetic field (3D electric and magnetic field components from DC up to AC frequencies) measurements are required to address the remaining questions.

For comets, we look forward to the launch of the ESA Comet Interceptor mission (Jones et al. 2024) by 2029. This mission will be the first to fly by a dynamically new comet (target yet to be identified) and with three spacecraft simultaneously, at different distances from the nucleus. Comet Interceptor is equipped with a suite of plasma and fields instruments on the main spacecraft, while the two minor probes both carry magnetometers and one of them also a plasma instrument. These measurements will shed new light on the global interaction between the solar wind and the comet, allowing us to assess the momentum and energy flow through the plasma environment. Where possible, comparative studies should continue to be made across all distant tail encounters with solar system objects, be they attached to cometary, or induced or intrinsic planetary magnetospheres.
